# Rhodium-Promoted C–H Bond Activation of Quinoline, Methylquinolines, and Related Mono-Substituted Quinolines

**DOI:** 10.1021/acs.organomet.2c00270

**Published:** 2022-08-11

**Authors:** Laura A. de las Heras, Miguel A. Esteruelas, Montserrat Oliván, Enrique Oñate

**Affiliations:** Departamento de Química Inorgánica - Instituto de Síntesis Química y Catálisis Homogénea (ISQCH)—Centro de Innovación en Química Avanzada (ORFEO-CINQA), Universidad de Zaragoza—CSIC, 50009 Zaragoza, Spain

## Abstract

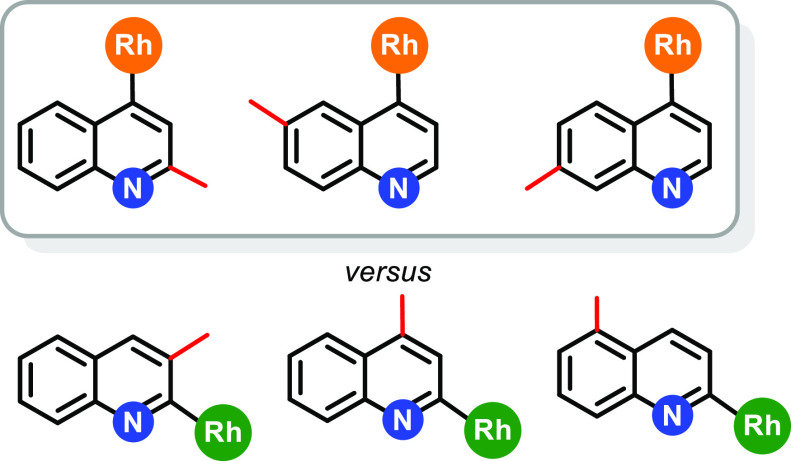

The C–H bond activation of methylquinolines, quinoline, 3-methoxyquinoline, and 3-(trifluoromethyl)quinoline promoted by the square-planar rhodium(I) complex RhH{κ^3^-P,O,P-[xant(P^i^Pr_2_)_2_]} [**1**; xant(P^i^Pr_2_)_2_ = 9,9-dimethyl-4,5-bis(diisopropylphosphino)xanthene] has been systematically studied. Results reveal that the activation of the heteroring is preferred over the activation of the carbocycle, and the activated position depends upon the position of the substituent in the substrate. Thus, 3-, 4-, and 5-methylquinoline reacts with **1** to quantitatively form square-planar rhodium(I)-(2-quinolinyl) derivatives, whereas 2-, 6-, and 7-methylquinoline quantitatively leads to rhodium(I)-(4-quinolinyl) species. By contrast, quinoline and 8-methylquinoline afford mixtures of the respective rhodium(I)-(2-quinolinyl) and -(4-quinolinyl) complexes. 3-Methoxyquinoline displays the same behavior as that of 3-methylquinoline, while 3-(trifluoromethyl)quinoline yields a mixture of rhodium(I)-(2-quinolinyl), -(4-quinolinyl), -(6-quinolinyl), and -(7-quinolinyl) isomers.

## Introduction

Direct C–H bond functionalization is a powerful and environmentally sustainable procedure, which converts simple compounds into valuable molecules, the C–H rupture being the key step in the processes of this class.^1^ The strength of the different C–H bonds of an organic substrate is in a narrow range, as proven by their dissociation energies; the range is particularly narrow when the entire molecule is aromatic. Transition metal complexes display ability to modify in a different manner the activation energy of the rupture of the diverse aromatic C–H bonds, as a function of their position at the aromatic ring and of the electronic and steric properties of the substituents of such a ring. Accordingly, to selectively perform the cleavage of a particular C–H bond, a metal species is usually needed.^2^ It is well established that the first step for a metal-promoted C–H bond rupture is the formation of a σ-intermediate L_*n*_M(η^2^-HC), which subsequently evolves by heterolytic or homolytic cleavage of the coordinated C–H bond. In this way, the activation energy of the rupture process is dependent on the stability of the σ-intermediate and the dissociation energy of the coordinated C–H bond. High chemo- and regioselectivities have been achieved by the kinetic governing of the rupture through the fine tuning of the electron density on the metal center and the design of the free space around the metal core, by choosing the ligands of the coordination sphere.^3^ From a thermodynamic point of view, strengths of the possible M–C bonds in the product dominate the selectivity of the cleavage.^4^

A quinoline core is present in many natural products and as a unit is a remarkable scaffold for the assembly of new pharmacological and agrochemical entities, given the diverse biological activity of this heterocycle and its derivatives.^5^ Transition-metal-catalyzed C–H functionalization of quinolines is currently receiving great attention as an environmentally sustainable alternative to the classical functionalization procedures, which generate more wastes.^6^ Functionalization of the C2 position is well established with a variety of transition-metal catalysts.^7^ The functionalization of C3,^8^ C4,^9^ and C8^10^ positions has been also achieved, although it is always a real challenge. On the other hand, selective functionalization of C5–C7 positions requires the help of an assistant on some ligand of the metal coordination sphere of the catalyst and/or at an adjacent position of the substrate.^11^ The rationalization of these findings is certainly difficult, to a large extent due to the scarce number of systematic studies performed on the elemental reaction of metal-promoted C–H bond activation of quinolines. Such a stoichiometric process has been rarely observed at C2 and C8 positions. The activation of the C–H bond at the 2 position is facilitated by the proximity of the nitrogen atom, which many times acts as a coordination assistant,^12^ whereas the rupture of the C–H bond at the 8 position takes place thanks to the formation of five-membered metalacycles involving also the nitrogen.^13^

**Scheme 1:**
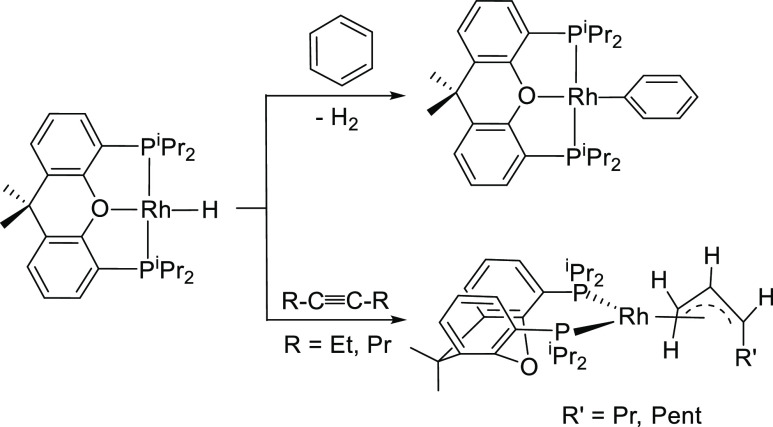
C(sp^2^)–H and C(sp^3^)–H Bond Activation of Arenes and Bis(alkyl)alkynes

Rhodium catalysts are among the most utilized for the functionalization of quinolines, in particular, to build C–C, C–N, and C–halogen bonds.^14^ The square-planar monohydride complex RhH{κ^3^-P,O,P-[xant(P^i^Pr_2_)_2_]} [xant(P^i^Pr_2_)_2_ = 9,9-dimethyl-4,5-bis(diisopropylphosphino)xanthene] is a rare stable member of the family of late-transition metal unsaturated monohydride complexes, which was initially prepared in only moderate yield.^15^ In spite of this unfortunate handicap, its chemistry was studied in some recent years, proving to promote the activation of a wide range of σ-bonds,^16^ including C(sp^2^)–H bonds of arenes^17^ and C(sp^3^)–H bonds of bis(alkyl)alkynes^18^ to afford aryl and allyl derivatives (Scheme 1). In agreement with such ability, it was observed to be an efficient catalyst for C–H bond functionalization reactions such as the borylation of arenes^17^ and the dehydrogenative borylation of bis(alkyl)alkynes.^18^ Furthermore, it catalyzes the hydroboration of alkynes,^19^ the deuteration of boranes and hydrides of group 14 elements,^20^ the ammonia borane dehydrogenation,^21^ and the dehydropolymerization of amine-boranes.^22^ Two years ago, we improved significantly its synthesis, reducing the reaction time and increasing the yield, which now reaches 90%.^20^ The interest in the rhodium catalysts in the C–H functionalization of quinolines, the enhancement of yield in the preparation of the monohydride RhH{κ^3^-P,O,P-[xant(P^i^Pr_2_)_2_]}, and its promising previous chemical behavior prompted us to employ this complex to perform a systematic study on the C–H bond activation of quinoline, its monomethyl-substituted counterparts, and related substituted quinolines.

This paper reports the C–H bond activation of quinoline and substituted quinolines promoted by RhH{κ^3^-P,O,P-[xant(P^i^Pr_2_)_2_]}, demonstrates that the activated position depends upon the position of the substituent and its nature, presents the selective formation of the first-metal-(4-quinolinyl) derivatives resulting from the direct C–H bond activation of a quinoline-type molecule, and reveals that the activation at 6 and 7 positions is also possible without the help of a coordination assistant.

## Results and Discussion

### C–H Bond Activation of Quinoline and Methylquinolines

**Scheme 2:**
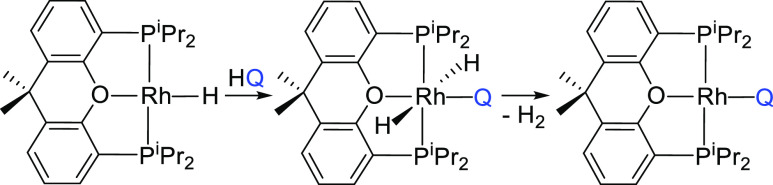
C–H Bond Activation of Quinoline and Methylquinolines

In addition to quinoline, the substrates used were 2-, 3-, 4-, 5-, 6-, 7-, and 8-methylquinoline. The study was performed in *n*-octane, at 80 °C, using 1:1 molar ratios of rhodium/heterocycle. Reactions were followed by ^31^P{^1^H} NMR spectroscopy for 48–72 h, until quantitative transformation of rhodium(I)-hydride RhH{κ^3^-P,O,P-[xant(P^i^Pr_2_)_2_]} (**1**) into the corresponding rhodium(I)-quinolinyl reaction products. Such species, which are the first square-planar transition metal complexes bearing a quinolinyl ligand, result from the direct C–H bond activation of the substrate (HQ) and the subsequent fast elimination of molecular hydrogen (Scheme 2). Although the trans-disposition of the hydride ligands appears to be favored in the rhodium(III)-dihydride intermediates similarly generated from **1** in related processes, the reductive elimination of molecular hydrogen rapidly occurs in all the cases because the hemilabile character of the oxygen atom of the ether-diphosphine allows the necessary three-center transition state.^16a,17^ The activated position depends on the position of the methyl substituent, with three different patterns of behavior being observed: selective activation of the C−H bond at position 2, selective activation of the C−H bond at position 4, and C−H bond activation competition between positions 2 and 4.

#### Selective Activation of the C–H Bond at Position 2

**Scheme 3:**
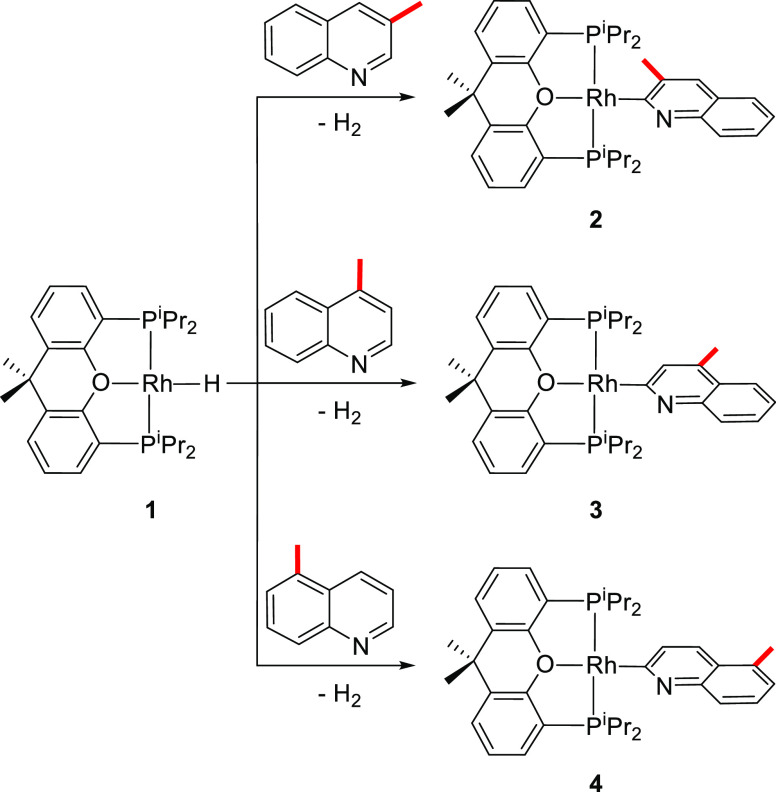
C–H Bond Activation of Position 2

A methyl group at 3, 4, and 5 positions directs the C–H bond activation of the mono-substituted heterocycle to the 2 position. Thus, treatment of **1** with 3-, 4-, and 5-methylquinoline quantitatively leads to the rhodium(I)-(2-quinolinyl) derivatives **2–4** (Scheme 3), which were isolated as yellow solids in moderate yields, about 40%, due to their high solubility in alkanes including pentane.

**Figure 1:**
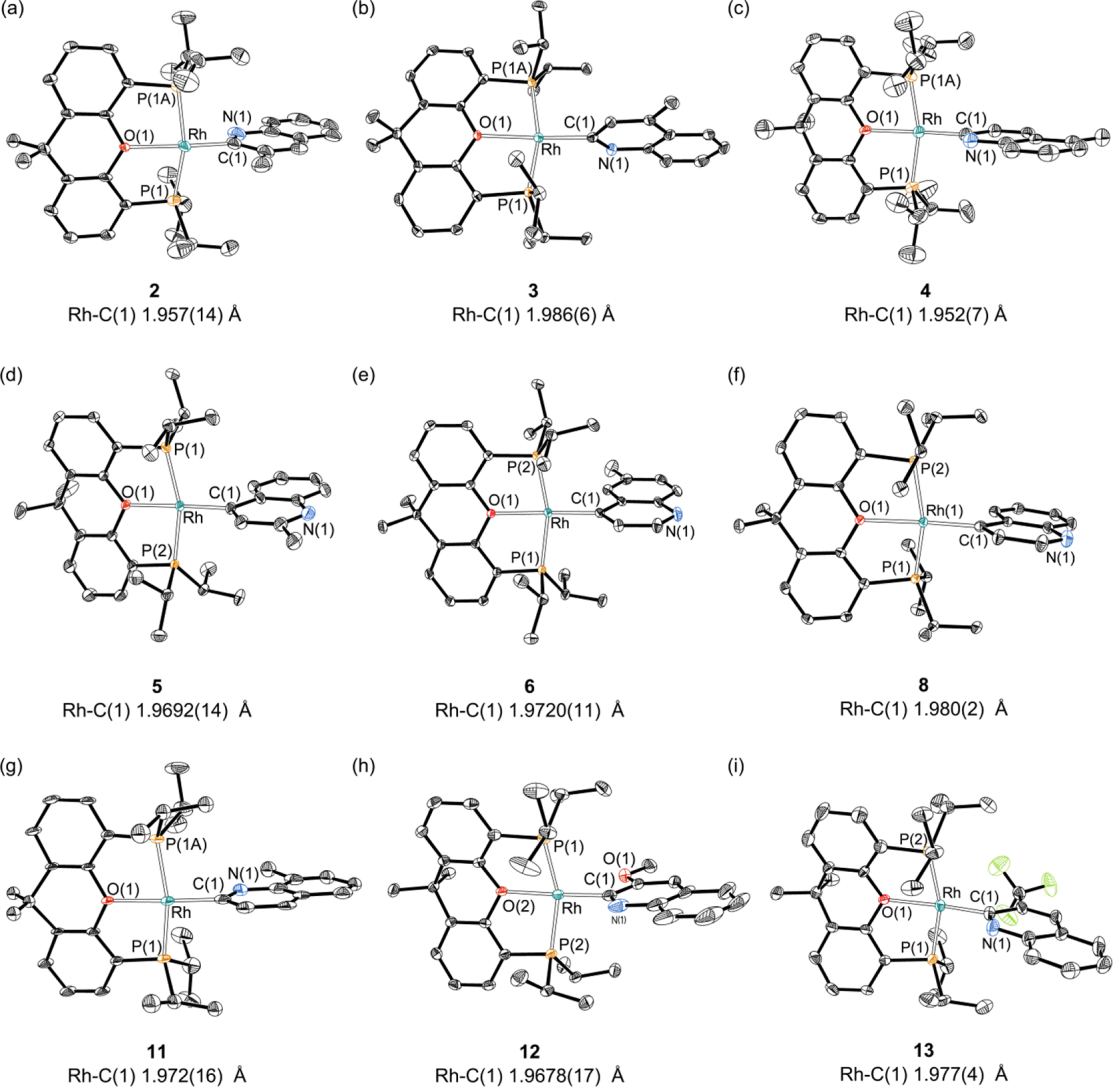
Molecular diagram of complexes **2–6**, **8**, and **11–13** [ellipsoids shown at 50% probability, except for **11** (30%)]. All hydrogen atoms are omitted for clarity.

The three compounds were characterized by X-ray diffraction analysis. Figure 1a–c gives views of the structures, which demonstrate the activation of the C–H bond at the 2 position and the square-planar environment of the rhodium atom with the ether-diphosphine mer-coordinated and the quinolinyl group trans-disposed to the oxygen atom. The Rh–C bond lengths are similar, being in the range 1.957(14)–1.986(6) Å. It should be noted that unlike the previous cases involving activation of the C–H bond at position 2,^12f^ coordination of the N atom is not observed despite the formally unsaturated character of the metal center. This is not entirely surprising given the ability of d^8^ ions to form square-planar species of 16 valence electrons. The NMR spectra are consistent with the structures (Figures S2–S10). Aromatic hydrogen atoms give rise to the patterns expected for the corresponding substitutions of the heterocycles, in the ^1^H spectra of the respective complexes (Figures S3, S6, and S9). The resonance due to the metalated carbon atom is observed as a doublet of triplets (^1^*J*_C–Rh_ ≈ 43 Hz, ^2^*J*_C–P_ ≈ 11 Hz), between 197 and 200 ppm, in the ^13^C{^1^H} spectra, whereas the ^31^P{^1^H} spectra display a doublet (^1^*J*_P–Rh_ ≈ 185 Hz), close to 37 ppm, in agreement with the equivalence of the P^i^Pr_2_ arms of the diphosphine.

Particularly noteworthy is the behavior of 3-methylquinoline. Such a substrate undergoes C–H bond activation in the heteroring in a sterically hindered position, adjacent to the methyl group, which makes it difficult for the C–H bond to approach the metal center. At first glance, this is surprising because the steric congestion around the carbocycle positions is significantly lower. On the other hand, it suggests that the activation of the heteroring positions is strongly favored with regard to those of the carbocycle for electronic reasons. The exclusivity in selection between positions 2 and 4 is also a remarkable finding as both are equally hampered by the methyl substituent.

#### Selective Activation of the C–H Bond at Position 4

**Scheme 4:**
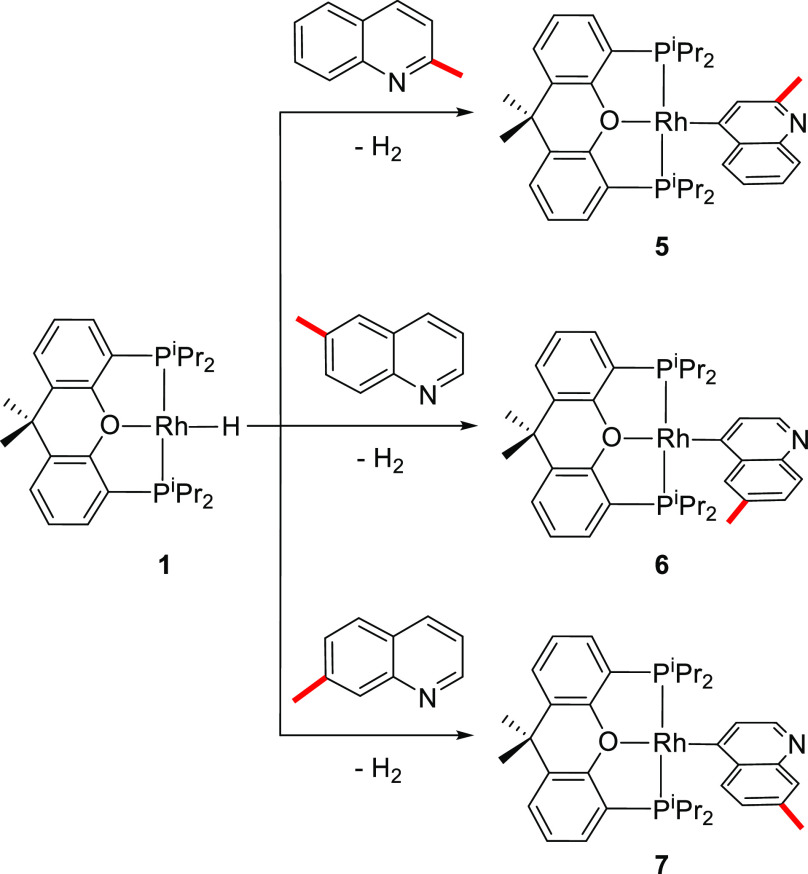
C–H Bond Activation of Position 4

A methyl group at 2, 6, and 7 positions of the mono-substituted quinoline directs the C–H bond activation to the 4 position, in contrast to the activations shown in Scheme 3. Thus, treatment of **1** with 2-, 6-, and 7-methylquinoline quantitatively affords the rhodium(I)-(4-quinolinyl) derivatives **5–7** (Scheme 4), which were isolated as yellow solids with yields from moderate to high (33–64%).

The formation of the first transition metal organometallic compounds resulting from the direct rupture of a C–H bond at the 4 position of a quinoline-type molecule was confirmed by the X-ray structures of **5** (Figure 1d) and **6** (Figure 1e). Both structures resemble those of **2–4** with Rh–C distances of 1.9692(14) (**5**) and 1.9720(11) (**6**) Å. The NMR spectra of **5–7** also support the activation of the aromatic C–H bond at the 4 position of the quinoline, mainly the ^1^H spectra (Figures S12, S15, and S18). Although the ^13^C{^1^H} and ^31^P{^1^H} spectra of these compounds and those of **2–4** are similar, there are between them enough differences to also convert these spectra into a characteristic feature of the activated position (Table 1). The ^13^C{^1^H} spectra of **5–7** display the doublet of triplets (^1^*J*_C–Rh_ = 41–44 Hz, ^2^*J*_C–P_ = 10–12 Hz) due to the metalated carbon atom of the heterocycle at about 187 ppm, shifted around 10 ppm toward a higher field with regard to the complexes **2–4**. In contrast, in the ^31^P{^1^H} spectra, the doublet generated by the equivalent P^i^Pr_2_ arms of the diphosphine appears at about 38 ppm, slightly shifted to a lower field, with values for the P–Rh coupling constant close to 171 Hz, which are about 14 Hz smaller than those observed in **2–4**.

**Table 1:** Selected Spectroscopic Data of Complexes **2–11**^a^

	^31^P{^1^H} NMR		^13^C{^1^H} NMR		
compound	δ	^1^*J*_P–Rh_	δ_Rh–C_	^1^*J*_C–Rh_	^2^*J*_C–P_
**2**	36.9 (d)	185.3	200.6 (dt)	44.5	11.4
**3**	37.4 (d)	185.3	197.9 (dt)	43.1	10.0
**4**	37.6 (d)	185.1	197.1 (dt)	43.9	11.1
**5**	38.3 (d)	171.7	187.0 (dt)	43.1	10.5
**6**	38.4 (d)	171.2	186.5 (dt)	41.9	12.2
**7**	38.3 (d)	171.2	187.5 (dt)	42.7	12.0
**8**	38.5 (d)	171.1	187.9 (dt)	43.0	11.7
**9**	37.7 (d)	184.7	196.0^b^		
**10**	38.1 (d)	171.8	188.3 (dt)	41.5	12.5
**11**	37.3 (d)	184.8	197.4 (dt)	43.1	10.9

a NMR spectra registered in benzene-*d*_6_.

b NMR spectrum registered in *n*-octane. Multiplicity not resolved.

Activation of the heteroring again was expected after observing the previous activations. However, the exclusive selection of the 4 position surprised us, given the activation of 3-methylquinoline at the 2 position and because the C–H bond at the 4 position is sterically more congested than those at 2 and 3 positions. One more time, steric reasons do not seem to be determinant in the C–H activation of these substrates. On the contrary, the substituent located in any position of both rings has a marked electronic repercussion in the activation of the C–H bonds of the heteroring; the activated C–H bond depends on the position of the methyl substituent, although the activation of the C–H bond in the 3 position seems hard to reach.

#### C–H Bond Activation Competition between Positions 2 and 4

**Scheme 5:**
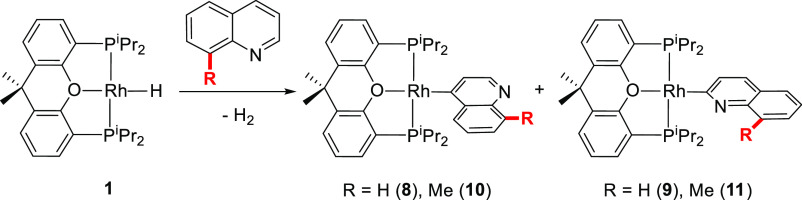
Competitive C–H Bond Activations of Positions 4 and 2

In contrast to the previously mentioned heterocycles, quinoline and 8-methylquinoline undergo the rupture of the C–H bonds at 2 and 4 positions in a competitive manner, with the activation of the C–H bond at the 3 position being definitely elusive (Scheme 5). Treatment of **1** with quinoline under the standard conditions of the study gives rise to the formation of a yellow solid (51% yield) and an orange solution. The yellow solid was characterized as the rhodium(I)-(4-quinolinyl) derivative **8**, by X-ray diffraction analysis. Figure 1f shows its structure [Rh–C = 1.980(2) Å]. The solution contains a mixture of **8** and its 2-quinolinyl isomer **9** in a 3:7 molar ratio. The presence of the latter is strongly supported by the NMR spectra of the mixture (Figures S23–S26). The ^1^H spectrum shows the expected pattern for a Rh substitution at the 2 position of the heterocycle (Figure S24), whereas the ^13^C{^1^H} spectrum contains a resonance at 196 ppm, and the ^31^P{^1^H} spectrum displays a doublet (^1^*J*_P–Rh_ = 184.7 Hz) at 37.7 ppm, in agreement with **2–4**. The reaction of **1** with 8-methylquinoline leads to a 1:1 mixture of the 4-quinolinyl derivative **10** and its 2-quinolinyl isomer **11**. Orange single crystals of the latter, suitable for X-ray analysis, were isolated from the mixture. Figure 1g gives a view of the structure [Rh–C = 1.972(16) Å]. Complex **10** was fully characterized by NMR spectroscopy. The spectra show characteristic features of Rh(I)-(4-quinolinyl) complexes (Figures S27–S31). In accordance with **5–7**, the ^13^C{^1^H} spectrum contains a doublet of triplets (^1^*J*_C–Rh_ = 41.5 Hz, ^2^*J*_C–P_ = 12.5 Hz) at 188.3 ppm, whereas the ^31^P{^1^H} spectrum displays a doublet (^1^*J*_P–Rh_ = 171.8 Hz) at 38.1 ppm.

The formation of **8–11** confirms that the C–H bond activation of quinoline can be selectively directed to the 2 or 4 position by the introduction of a methyl substituent at the appropriate place on the heterocycle, with the exception of the 8 position.

### C–H Bond Activation of Related Mono-Substituted Quinolines

Having established that a methyl group, located at any position of the quinoline rings, directs the C–H bond activation of the corresponding heterocycle to 2 or 4 positions and upon observing a scarce steric influence of the methyl group on the process, in contrast to the C–H bond activation of aromatic hydrocarbons,^17,23^ we decided to study the influence of markedly different substituents from an electronic point of view. We selected methoxide as a strongly electron-donating group, trifluoromethyl as a notable electron-withdrawing substituent, and the 3 position on being adjacent to both previously activated bonds.

**Scheme 6:**
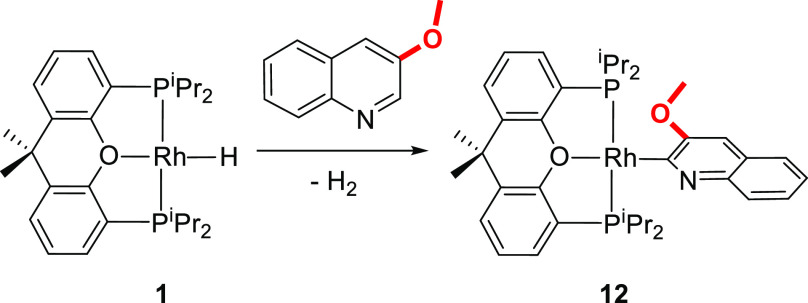
C–H Bond Activation of 3-Methoxyquinoline

The methoxide group does not change the rate or the selectivity of the activation with regard to methyl. Treatment of **1** with 3-methoxyquinoline quantitatively leads to a 2-quinolinyl derivative, **12** (Scheme 6), after 72 h as 3-methylquinoline. Complex **12** was isolated as an orange solid and characterized by X-ray diffraction analysis. Figure 1h shows a view of the structure. The Rh–C bond length of 1.9678(17) Å is statistically identical to that of the 3-methyl counterpart. The NMR spectra agree well with those of the 2-quinolinyl derivatives **2–4**, **9,** and **11** (Figures S32–S34). The ^13^C{^1^H} spectrum contains a doublet of triplets (^1^*J*_C–Rh_ = 45.1 Hz, ^2^*J*_C–P_ = 11.2 Hz) in the expected region, 192.9 ppm, whereas the ^31^P{^1^H} spectrum shows a doublet at 38.6 ppm with a P–Rh coupling constant displaying a value of 183.0 Hz, which lies in the same range as those of **2–4**, **9**, and **11**.

**Scheme 7:**
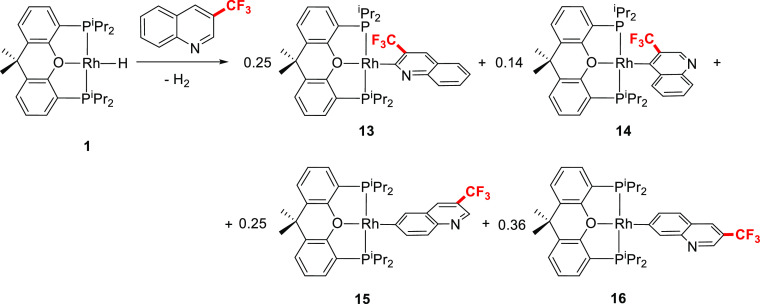
C–H Bond Activation of 3-(Trifluoromethyl)quinoline

The trifluoromethyl group introduces very noticeable changes. It slows down the rate of the reaction and dramatically reduces the selectivity, promoting the activations of the C–H bonds at 2, 4, 6, and 7 positions (Scheme 7). Thus, the treatment of **1** with 3-(trifluoromethyl)quinoline gives a mixture of derivatives **13** (2-quinolinyl, 25%), **14** (4-quinolinyl, 14%), **15** (6-quinolinyl, 25%), and **16** (7-quinolinyl, 36%), after 5 days, according to the ^31^P{^1^H} NMR spectrum of the resulting solution in *n*-octane (Figure S35). The presence of **13** and **14** in the mixture is supported by the values of the P–Rh coupling constants obtained from the respective doublets at 35.3 and 37.5 ppm, of 183.9 and 174.6 Hz, which, respectively, lie in the range of the coupling constants observed in the previous 2-quinolinyl and 4-quinolinyl complexes (see Table 1). The evaporation of *n*-octane affords a reaction crude that when subsequently treated with pentane, yields a red solid only formed by **15** and **16** in a 1:2.5 molar ratio. Such a solid allowed us to obtain fine ^1^H, (^1^H,^1^H)–correlated spectroscopy (COSY), nuclear Overhauser effect spectroscopy (NOESY), (^1^H,^13^C)-heteronuclear single quantum coherence (HSQC), and (^1^H,^13^C)-heteronuclear multiple bond correlation (HMBC) NMR spectra to fully characterize both **15** and **16** (see Figures S36–S42). A noticeable feature of **15** is a doublet (^1^*J*_P–Rh_ = 172.8 Hz) at 37.8 ppm in the ^31^P{^1^H} spectrum, whereas remarkable resonances of **16** are a doublet (^1^*J*_P–Rh_ = 173.7 Hz) at 38.0 ppm in the ^31^P{^1^H} spectrum and a doublet of triplets (^1^*J*_C–Rh_ = 40.4 Hz, ^2^*J*_C–P_ = 12.5 Hz) at 179.7 ppm in the ^13^C{^1^H} spectrum. The formation of the 2-quinolinyl derivative **13**, the 6-quinolinyl complex **15**, and the 7-quinolinyl compound **16**, as a result of the C–H bond activation of 3-(trifluoromethyl)quinoline, was furthermore confirmed by orange and red crystals obtained from the original solution in *n*-octane. X-ray diffraction of an orange single crystal provided the structure of **13** (Figure 1i), which showed a Rh–C distance of 1.977(4) Å. The X-ray diffraction analysis of a red crystal revealed that it was a pseudo-merohedral twin resulting from the co-crystallization of **15** and **16**, in the same unit cell, in an approximately 1:2 proportion. Although the results of the analysis are not enough accurate to permit a deep discussion of the structural parameters, they confirm the bond sequence in both **15** and **16** and therefore their formation.

The behavior of 3-(trifluoromethyl)quinoline points out that the introduction of an electron-withdrawing substituent at the heteroring allows the direct activation of the C–H bond at the elusive positions 6 and 7 of the heterocycle, although the selectivity of the activation is significantly reduced.

## Concluding Remarks

This study reveals that complex RhH{κ^3^-P,O,P-[xant(P^i^Pr_2_)_2_]} promotes the C–H bond activation of quinoline and mono-substituted quinolines, to form square-planar rhodium(I)-quinolinyl derivatives. Activation of the heteroring is preferred over activation of the carbocycle. Such selectivity could be related to some contribution of an anionic carbene resonance form to the rhodium–quinolinyl bond. The chemical shifts of the resonance corresponding to the metalated carbon, in a relatively low field, in the ^13^C{^1^H} NMR spectra seem to point out in this direction. Within the heteroring, the 3 position is always elusive, while it is possible to select between activation of 2 and 4 positions by the introduction of a methyl substituent at the appropriate place on the heterocycle, with the exception of the 8 position. The presence of an electron-withdrawing substituent such as trifluoromethyl at the 3 position of the heteroring allows us to activate the carbocycle at 6 and 7 positions, although the selectivity of the activation suffers a significant decrease.

In summary, the first systematic study on the transition metal-promoted C–H bond activation of substituted quinolines yielded clear patterns of behavior, which allow us to control the selectivity of the activation and to observe direct C–H bond activations of unprecedented positions.

## Experimental Section

### General Information

All reactions were carried out with exclusion of air using Schlenk-tube techniques or in a drybox. Instrumental methods and X-ray details are given in the Supporting Information. In the NMR spectra (Figures S2–S42), the chemical shifts (in ppm) are referenced to residual solvent peaks (^1^H, ^13^C{^1^H}) or external 85% H_3_PO_4_ (^31^P{^1^H}), while *J* and *N* (*N* = *J*_P–H_ + *J*_P′–H_ for ^1^H and *N* = *J*_P–C_ + *J*_P′–C_ for ^13^C{^1^H}) are given in hertz. RhH{κ^3^-P,O,P-[xant(P^i^Pr_2_)_2_]} (**1**)^20^ was prepared according to the reported procedure.

### Reaction of 1 with 3-Methylquinoline: Preparation of Rh(κ^1^-C^2^-Quinolinyl-3-Me){κ^3^-P,O,P-[xant(P^i^Pr_2_)_2_]} (**2**)

A solution of **1** (200 mg, 0.37 mmol) in *n*-octane (3 mL) was treated with 3-methylquinoline (49 μL, 0.37 mmol), and the resulting mixture was stirred at 80 °C for 48 h. After this time, the solution was evaporated to dryness to afford a brown residue. Addition of pentane (4 mL) afforded an orange solid that was washed with pentane (2 × 2 mL) and dried in vacuo. Yield: 67 mg (26%). The reaction is quantitative, but the isolated yield is low due to its high solubility in pentane. The solid is extremely sensitive to air, preventing us from getting its elemental analysis. HRMS (electrospray, *m*/*z*): calcd for C_37_H_49_NOP_2_Rh [M + H]^+^, 688.2339; found, 688.2325. IR (cm^–1^): ν(C=N) 1585 (m), ν(C–O–C) 1150 (m). ^1^H NMR (300.13 MHz, benzene-*d*_6_, 298 K): δ 8.25 (d, ^3^*J*_H–H_ = 8.3, 1H, CH C-ring qn), 7.63 (d, ^3^*J*_H–H_ = 8.0, 1H, CH C-ring qn), 7.49 (m, 1H, CH C-ring qn), 7.33 (s, 1H, CH N-ring qn), 7.30–7.19 (m, 3H, 2H CH-arom POP + 1H CH C-ring qn), 7.07 (dd, ^3^*J*_H–H_ = 7.7, ^4^*J*_H–H_ = 1.5, 2H, CH-arom POP), 6.87 (t, ^3^*J*_H–H_ = 7.5, 2H, CH-arom POP), 3.32 (s, 3H, CH_3_ qn), 2.52 (m, 2H, PC*H*(CH_3_)_2_), 2.20 (m, 2H, PC*H*(CH_3_)_2_), 1.13 (s, 6H, CH_3_), 1.36–0.91 (m, 24H, PCH(C*H*_3_)_2_). ^13^C{^1^H}-apt NMR (75.48 MHz, benzene-*d*_6_, 298 K): δ 200.6 (dt, ^1^*J*_C–Rh_ = 44.5, ^2^*J*_C–P_ = 11.4, Rh–C qn), 157.0 (vt, *N* = 16.0, C-arom POP), 147.1 (d, ^3^*J*_C–Rh_ = 3.6, C qn), 138.0 (s, *C*–CH_3_ qn), 131.8 (s, C-arom POP), 131.0 (s, CH-arom POP), 128.7 (s, CH C-ring qn), 128.1 (s, CH C-ring qn, inferred from the HSQC spectrum), 127.4 (s, CH C-ring qn), 126.9 (s, CH-arom POP), 126.5 (s, CH C-ring qn), 125.7 (vt, *N* = 16.5, C-arom POP), 125.1 (s, C qn), 124.7 (d, ^2^*J*_C–Rh_ = 8.9, CH N-ring qn), 124.2 (s, CH-arom POP), 121.4 (s, CH C-ring qn), 34.5 (s, *C*(CH_3_)_2_), 31.5 (s, C(*C*H_3_)_2_), 25.8 (m, P*C*H(CH_3_)_2_), 26.0 (s, CH_3_ qn), 25.1 (m, P*C*H(CH_3_)_2_), 19.0 (vt, *N* = 9.7, PCH(*C*H_3_)_2_), 18.6, 18.3, 18.1 (all s, PCH(*C*H_3_)_2_). ^31^P{^1^H} NMR (121.49 MHz, benzene-*d*_6_, 298 K): δ 36.9 (d, ^1^*J*_Rh–P_ = 185.3).

### Reaction of 1 with 4-Methylquinoline: Preparation of Rh(κ^1^-C^2^-Quinolinyl-4-Me){κ^3^-P,O,P-[xant(P^i^Pr_2_)_2_]} (**3**)

A solution of **1** (200 mg, 0.37 mmol) in *n*-octane (3 mL) was treated with 4-methylquinoline (49 μL, 0.37 mmol), and the resulting mixture was stirred at 80 °C for 48 h. After this time, the solution was evaporated to dryness to afford a brown residue. Addition of pentane (4 mL) afforded an orange solid that was washed with pentane (2 × 2 mL) and dried in vacuo. Yield: 96 mg (38%). The reaction is quantitative, but the isolated yield is low due to its high solubility in pentane. Anal. Calcd. for C_37_H_48_NOP_2_Rh: C, 64.63; H, 7.04; N, 2.04. Found; 64.84; H, 6.98; N, 2.03. HRMS (electrospray, *m*/*z*): calcd for C_37_H_49_NOP_2_Rh [M + H]^+^, 688.2339; found, 688.2343. IR (cm^–1^): ν(C=N) 1576 (m), ν(C–O–C) 1194 (m). ^1^H NMR (300.13 MHz, benzene-*d*_6_, 298 K): δ 8.35 (d, ^3^*J*_H–H_ = 8.3, 1H, CH C-ring qn), 8.01 (s, 1H, CH N-ring qn), 7.80 (d, ^3^*J*_H–H_ = 9.1, 1H, CH C-ring qn), 7.5 (m, 1H, CH C-ring qn), 7.3 (m, 2H, CH-arom POP), 7.20 (m, 1H, CH C-ring qn), 7.05 (dd, ^3^*J*_H–H_ = 7.7, ^4^*J*_H–H_ = 1.5, 2H, CH-arom POP), 6.86 (t, ^3^*J*_H–H_ = 7.7, 2H, CH-arom POP), 2.45 (m, 4H, PC*H*(CH_3_)_2_), 2.42 (s, 3H, CH_3_ qn), 1.24 (s, 6H, CH_3_), 1.22 (dvt, ^3^*J*_H–H_ = 7.4, *N* = 14.5, 24H, PCH(C*H*_3_)_2_). ^13^C{^1^H}-apt NMR (75.48 MHz, benzene-*d*_6_, 298 K): δ 197.9 (dt, ^1^*J*_C–Rh_ = 43.1, ^2^*J*_C–P_ = 10.0, Rh–C qn), 156.2 (vt, *N* = 15.8, C-arom POP), 148.6 (d, ^3^*J*_C–Rh_ = 3.8, C qn), 135.6 (s, CH N-ring qn), 131.3 (s, CH-arom POP), 131.1 (vt, *N* = 5.2, C-arom POP), 130.4 (s, C qn), 128.7 (s, CH C-ring qn), 127.5 (s, CH-arom POP), 127.3 (s, CH C-ring qn), 126.0 (vt, *N* = 16.2, C-arom POP), 125.8 (s, *C*–CH_3_ qn), 124.3 (s, CH C-ring qn), 124.1 (s, CH-arom POP), 121.3 (s, CH C-ring qn), 34.2 (s, *C*(CH_3_)_2_), 32.4 (s, C(*C*H_3_)_2_), 25.8 (dvt, ^2^*J*_C–Rh_ = 2.5, *N* = 18.1, P*C*H(CH_3_)_2_), 19.5 (vt, *N* = 9.0, PCH(*C*H_3_)_2_), 18.9 (s, PCH(*C*H_3_)_2_), 18.3 (s, CH_3_ qn). ^31^P{^1^H} NMR (121.49 MHz, benzene-*d*_6_, 298 K): δ 37.4 (d, ^1^*J*_Rh–P_ = 185.3).

### Reaction of 1 with 5-Methylquinoline: Preparation of Rh(κ^1^-C^2^-Quinolinyl-5-Me){κ^3^-P,O,P-[xant(P^i^Pr_2_)_2_]} (**4**)

A solution of **1** (200 mg, 0.37 mmol) in *n*-octane (3 mL) was treated with 5-methylquinoline (49 μL, 0.37 mmol), and the resulting mixture was stirred at 80 °C for 48 h. After this time, the solution was evaporated to dryness to afford a brown residue. Addition of pentane (4 mL) afforded an orange solid that was washed with pentane (2 × 2 mL) and dried in vacuo. Yield: 95 mg (38%). The reaction is quantitative, but the isolated yield is low due to its high solubility in pentane. Anal. Calcd. for C_37_H_48_NOP_2_Rh: C, 64.63; H, 7.04; N, 2.04. Found; C, 64.89; H, 7.32; N, 2.18. HRMS (electrospray, *m*/*z*): calcd for C_37_H_49_NOP_2_Rh [M + H]^+^, 688.2339; found, 688.2321. IR (cm^–1^): ν(C=N) 1587 (m), ν(C–O–C) 1188 (m). ^1^H NMR (300.13 MHz, benzene-*d*_6_, 298 K): δ 8.19 (d, ^3^*J*_H–H_ = 8.3, 1H, CH C-ring qn), 8.10 (d, ^3^*J*_H–H_ = 8.7, 1H, CH N-ring qn), 7.49–7.37 (m, 2H, CH C- and N-rings qn), 7.27 (m, 2H, CH-arom POP), 7.06 (dd, ^3^*J*_H–H_ = 7.0, ^4^*J*_H–H_ = 1.3, 2H, CH-arom POP), 7.00 (d, ^2^*J*_H–H_ = 6.9, 1H, CH N-ring qn), 6.87 (t, ^3^*J*_H–H_ = 7.6, 2H, CH-arom POP), 2.47 (s, 3H, CH_3_ qn), 2.45 (m, 4H, PC*H*(CH_3_)_2_), 1.25 (s, 6H, CH_3_), 1.23 (dvt, ^3^*J*_H–H_ = 7.4, *N* = 14.4, 24H, PCH(C*H*_3_)_2_). ^13^C{^1^H}-apt NMR (75.48 MHz, benzene-*d*_6_, 298 K): δ 197.1 (dt, ^1^*J*_C–Rh_ = 43.9, ^2^*J*_C–P_ = 11.1, Rh–C qn), 156.2 (vt, *N* = 16.0, C-arom POP), 149.2 (d, ^3^*J*_C–Rh_ = 3.5, C qn), 134.5 (dt, ^2^*J*_C–Rh_ = 3.4, ^3^*J*_C–P_ = 3.4, CH N-ring qn), 134.3 (s, *C*–CH_3_ qn), 131.4 (s, CH-arom POP), 131.1 (vt, *N* = 5.1, C-arom POP), 127.5 (s, CH-arom POP), 127.1 (s, CH C-ring qn), 126.0 (vt, *N* = 15.5, C-arom POP), 124.4 (s, C qn), 124.1 (s, CH-arom POP), 122.6 (s, CH C-ring qn), 121.0 (s, CH N-ring qn), 34.2 (s, *C*(CH_3_)_2_), 32.4 (s, C(*C*H_3_)_2_), 25.8 (dvt, ^2^*J*_C–Rh_ = 2.6, *N* = 18.1, P*C*H(CH_3_)_2_), 19.6 (vt, *N* = 8.7, PCH(*C*H_3_)_2_), 18.9 (s, PCH(*C*H_3_)_2_), 18.7 (s, CH_3_ qn). ^31^P{^1^H} NMR (121.49 MHz, benzene-*d*_6_, 298 K): δ 37.6 (d, ^1^*J*_Rh–P_ = 185.1).

### Reaction of 1 with 2-Methylquinoline: Preparation of Rh(κ^1^-C^4^-Quinolinyl-2-Me){κ^3^-P,O,P-[xant(P^i^Pr_2_)_2_]} (**5**)

A solution of **1** (200 mg, 0.37 mmol) in *n*-octane (3 mL) was treated with 2-methylquinoline (50 μL, 0.37 mmol), and the resulting mixture was stirred at 80 °C for 72 h. After this time, the solution was evaporated to dryness to afford a brown residue. Addition of pentane (4 mL) afforded a yellow solid that was washed with pentane (2 × 2 mL) and dried in vacuo. Yield: 161.7 mg (64%). Anal. Calcd. for C_37_H_48_NOP_2_Rh: C, 64.63; H, 7.04; N, 2.04. Found; C, 64.24; H, 7.32; N, 1.87. HRMS (electrospray, *m*/*z*): calcd for C_37_H_49_NOP_2_Rh [M + H]^+^, 688.2339; found, 688.2326. IR (cm^–1^): ν(C=N) 1546 (m), ν(C–O–C) 1197 (m). ^1^H NMR (300.13 MHz, benzene-*d*_6_, 298 K): δ 9.48 (m, 1H, CH C-ring qn), 8.40 (m, 1H, CH C-ring qn), 8.09 (d, ^3^*J*_H–Rh_ = 2.4, 1H, CH N-ring qn), 7.5 (m, 2H, CH C-ring qn), 7.17 (m, 2H, CH-arom POP), 7.05 (dd, ^3^*J*_H–H_ = 7.7, ^4^*J*_H–H_ = 1.5, 2H, CH-arom POP), 6.84 (t, ^3^*J*_H–H_ = 7.6, 2H, CH-arom POP), 2.88 (s, 3H, CH_3_ qn), 2.38 (m, 2H, PC*H*(CH_3_)_2_), 2.14 (m, 2H, PC*H*(CH_3_)_2_), 1.30 (s, 3H, CH_3_), 1.19 (s, 3H, CH_3_), 1.02 (dvt, ^3^*J*_H–H_ = 6.8, *N* = 15.4, 12H, PCH(C*H*_3_)_2_), 1.02 (dvt, ^3^*J*_H–H_ = 7.5, *N* = 14.4, 6H, PCH(C*H*_3_)_2_), 0.81 (dvt, ^3^*J*_H–H_ = 9.2, *N* = 16.3, 6H, PCH(C*H*_3_)_2_). ^13^C{^1^H}-apt NMR (75.48 MHz, benzene-*d*_6_, 298 K): δ 187.0 (dt, ^1^*J*_C–Rh_ = 43.1, ^2^*J*_C–P_ = 10.5, Rh–C qn), 156.1 (vt, *N* = 15.6, C-arom POP), 152.6 (s, *C*–CH_3_ qn), 147.5 (s, C qn), 138.7 (s, CH C-ring qn), 138.4 (s, C qn), 132.7 (s, CH N-ring qn), 131.1 (s, CH-arom POP), 131.0 (vt, *N* = 5.0, C-arom POP), 130.0 (s, CH C-ring qn), 128.0 (s, CH-arom POP, inferred from the HSQC spectrum), 127.3 (s, CH C-ring qn), 125.0 (vt, *N* = 16.2, C-arom POP), 124.3 (s, CH-arom POP), 120.9 (s, CH C-ring qn), 34.6 (s, C(*C*H_3_)_2_), 34.1 (s, *C*(CH_3_)_2_), 31.0 (s, C(*C*H_3_)_2_), 25.7 (vt, *N* = 18.4, P*C*H(CH_3_)_2_), 25.6 (s, CH_3_ qn), 24.9 (vt, *N* = 15.1, P*C*H(CH_3_)_2_), 19.2 (vt, *N* = 6.8, PCH(*C*H_3_)_2_), 18.5 (vt, *N* = 8.3, PCH(*C*H_3_)_2_), 18.3, 18.1 (both s, PCH(*C*H_3_)_2_). ^31^P{^1^H} NMR (121.49 MHz, benzene-*d*_6_, 298 K): δ 38.3 (d, ^1^*J*_Rh–P_ = 171.7).

### Reaction of 1 with 6-Methylquinoline: Preparation of Rh(κ^1^-C^4^-Quinolinyl-6-Me){κ^3^-P,O,P-[xant(P^i^Pr_2_)_2_]} (**6**)

A solution of **1** (200 mg, 0.37 mmol) in *n*-octane (3 mL) was treated with 6-methylquinoline (49 μL, 0.37 mmol), and the resulting mixture was stirred at 80 °C for 48 h. After this time, the solution was evaporated to dryness to afford a brown residue. Addition of pentane (4 mL) afforded a yellow solid that was washed with pentane (2 × 2 mL) and dried in vacuo. Yield: 156 mg (62%). Anal. Calcd. for C_37_H_48_NOP_2_Rh: C, 64.63; H, 7.04; N, 2.04. Found: C, 64.31; H, 6.88; N, 2.30. HRMS (electrospray, *m*/*z*): calcd for C_37_H_49_NOP_2_Rh [M + H]^+^, 688.2339; found, 688.2324. IR (cm^–1^): ν(C=N) 1547 (m), ν(C–O–C) 1198 (m). ^1^H NMR (300.13 MHz, benzene-*d*_6_, 298 K): δ 9.36 (s, 1H, CH C-ring qn), 8.62 (d, ^3^*J*_H–H_ = 4.6, 1H, CH N-ring qn), 8.42 (d, ^3^*J*_H–H_ = 8.5, 1H, CH C-ring qn), 8.07 (m, 1H, CH N-ring qn), 7.35 (dd, ^3^*J*_H–H_ = 8.5, ^4^*J*_H–H_ = 2.1, H, CH C-ring qn), 7.18 (m, 2H, CH-arom POP), 7.04 (d, ^3^*J*_H–H_ = 7.6, ^4^*J*_H–H_ = 1.5, 2H, CH-arom POP), 6.84 (t, ^3^*J*_H–H_ = 5.6, 2H, CH-arom POP), 2.55 (s, 3H, CH_3_ qn), 2.34 (m, 2H, PC*H*(CH_3_)_2_), 2.16 (m, 2H, PC*H*(CH_3_)_2_), 1.29 (s, 3H, CH_3_), 1.19 (s, 3H, CH_3_), 1.19–1.02 (m, 18H, PCH(C*H*_3_)_2_), 0.82 (dvt, ^3^*J*_H–H_ = 8.8, *N* = 16.2, 6H, PCH(C*H*_3_)_2_). ^13^C{^1^H}-apt NMR (100.62 MHz, benzene-*d*_6_, 298 K): δ 186.5 (dt, ^1^*J*_C–Rh_ = 41.9, ^2^*J*_C–P_ = 12.2, Rh–C qn), 156.0 (vt, *N* = 15.6, C-arom POP), 146.3 (s, C qn), 145.0 (s, CH N-ring qn), 140.5 (s, C qn), 138.0 (s, CH C-ring qn), 132.6 (s, CH N-ring qn), 131.2 (s, CH-arom POP), 130.9 (vt, *N* = 4.9, C-arom POP), 130.5 (s, CH C-ring qn), 130.1 (s, *C*–CH_3_ qn), 129.3 (s, CH C-ring qn), 128.4 (s, CH-arom POP), 125.0 (vt, *N* = 16.3, C-arom POP), 124.3 (s, CH-arom POP), 34.5 (s, C(*C*H_3_)_2_), 34.1 (s, *C*(CH_3_)_2_), 31.4 (s, C(*C*H_3_)_2_), 25.6 (dvt, ^2^*J*_C–Rh_ = 2.0, *N* = 18.7, P*C*H(CH_3_)_2_), 24.8 (dvt, ^2^*J*_C–Rh_ = 2.7, *N* = 19.0, P*C*H(CH_3_)_2_), 21.8 (s, CH_3_ qn), 19.3 (vt, *N* = 7.1, PCH(*C*H_3_)_2_), 18.4 (vt, *N* = 8.7, PCH(*C*H_3_)_2_), 18.3, 18.0 (both s, PCH(*C*H_3_)_2_). ^31^P{^1^H} NMR (161.98 MHz, benzene-*d*_6_, 298 K): δ 38.4 (d, ^1^*J*_Rh–P_ = 171.2).

### Reaction of 1 with 7-Methylquinoline: Preparation of Rh(κ^1^-C^4^-Quinolinyl-7-Me){κ^3^-P,O,P-[xant(P^i^Pr_2_)_2_]} (**7**)

A solution of **1** (200 mg, 0.37 mmol) in *n*-octane (3 mL) was treated with 7-methylquinoline (49 μL, 0.37 mmol), and the resulting mixture was stirred at 80 °C for 48 h. After this time, the solution was evaporated to dryness to afford a brown residue. Addition of pentane (4 mL) afforded a yellow solid that was washed with pentane (2 × 2 mL) and dried in vacuo. Yield: 84 mg (33%). The reaction is quantitative, but the isolated yield is low due to its high solubility in pentane. Anal. Calcd. for C_37_H_48_NOP_2_Rh: C, 64.63; H, 7.04; N, 2.04. Found; 64.93; H, 6.78; N, 2.23. HRMS (electrospray, *m*/*z*): calcd for C_37_H_49_NOP_2_Rh [M + H]^+^, 688.2339; found, 688.2342. IR (cm^–1^): ν(C=N) 1547 (m), ν(C–O–C) 1196 (m). ^1^H NMR (400.13 MHz, benzene-*d*_6_, 298 K): δ 9.44 (d, ^3^*J*_H–H_ = 7.8, 1H, CH C-ring qn), 8.60 (d, ^3^*J*_H–H_ = 4.5, 1H, CH N-ring qn), 8.27 (s, 1H, CH C-ring qn), 8.05 (m, 1H, CH N-ring qn), 7.36 (d, ^3^*J*_H–H_ = 7.8, 1H, CH C-ring qn), 7.19 (m, 2H, CH-arom POP), 7.07 (d, ^3^*J*_H–H_ = 7.0, 2H, CH-arom POP), 6.85 (t, ^3^*J*_H–H_ = 7.5, 2H, CH-arom POP), 2.38 (s, 3H, CH_3_ qn), 2.36 (m, 2H, PC*H*(CH_3_)_2_), 2.17 (m, 2H, PC*H*(CH_3_)_2_), 1.31 (s, 3H, CH_3_), 1.21 (s, 3H, CH_3_), 1.11 (m, 12H, PCH(C*H*_3_)_2_), 1.05 (dvt, ^3^*J*_H–H_ = 7.4, *N* = 14.4, 6H, PCH(C*H*_3_)_2_), 0.82 (dvt, ^3^*J*_H–H_ = 7.4, *N* = 15.7, 6H, PCH(C*H*_3_)_2_). ^13^C{^1^H}-apt NMR (100.62 MHz, benzene-*d*_6_, 298 K): δ 187.5 (dt, ^1^*J*_C–Rh_ = 42.7, ^2^*J*_C–P_ = 12.0, Rh–C qn), 156.0 (vt, *N* = 15.3, C-arom POP), 148.1 (s, C qn), 145.5 (s, CH N-ring qn), 138.9 (s, C qn), 138.7 (s, CH C-ring qn), 136.5 (s, *C*–CH_3_ qn), 132.0 (s, CH N-ring qn), 131.2 (s, CH-arom POP), 130.9 (vt, *N* = 4.8, C-arom POP), 129.9 (s, CH C-ring qn), 128.0 (s, CH-arom POP, inferred from the HSQC spectrum), 125.0 (vt, *N* = 14.5, C-arom POP), 124.3 (s, CH-arom POP), 123.7 (s, CH C-ring qn), 34.5 (s, C(*C*H_3_)_2_), 34.1 (s, *C*(CH_3_)_2_), 31.3 (s, C(*C*H_3_)_2_), 25.6 (dvt, ^2^*J*_C–Rh_ = 1.6, *N* = 18.9, P*C*H(CH_3_)_2_), 24.8 (dvt, ^2^*J*_C–Rh_ = 2.5, *N* = 18.9, P*C*H(CH_3_)_2_), 21.8 (s, CH_3_ qn), 19.3 (vt, *N* = 6.0, PCH(*C*H_3_)_2_), 18.4, 18.0 (both s, PCH(*C*H_3_)_2_). ^31^P{^1^H} NMR (161.98 MHz, benzene-*d*_6_, 298 K): δ 38.3 (d, ^1^*J*_Rh–P_ = 171.2).

### Reaction of **1** with Quinoline

A solution of **1** (200 mg, 0.37 mmol) in *n*-octane (3 mL) was treated with quinoline (43 μL, 0.37 mmol), and the resulting mixture was stirred at 80 °C for 48 h. After this time, the obtained suspension was evaporated to dryness to afford a brown residue. Addition of pentane (4 mL) afforded a yellow solid that was washed with pentane (2 × 2 mL) and dried in vacuo. Yield: 126 mg (51%). The ^31^P{^1^H} NMR spectra of the mother liquors show the presence of complexes Rh(κ^1^-C^4^-quinolinyl){κ^3^-P,O,P-[xant(P^i^Pr_2_)_2_]} (**8**) and Rh(κ^1^-C^2^-quinolinyl){κ^3^-P,O,P-[xant(P^i^Pr_2_)_2_]} (**9**) in a ratio 37:63, while the NMR spectra of the solid only show the presence of **8**, that was obtained in a pure form due to the different solubility of **8** and **9** in pentane.

#### Data for **8**

Anal. Calcd. for C_36_H_46_NOP_2_Rh: C, 64.19; H, 6.88; N, 2.08. Found; 63.82; H, 7.00; N, 2.23. HRMS (electrospray, *m*/*z*): calcd for C_36_H_47_NOP_2_Rh [M + H]^+^, 674.2182; found, 674.2198. IR (cm^–1^): ν(C=N) 1548 (m), ν(C–O–C) 1196 (m). ^1^H NMR (300.13 MHz, benzene-*d*_6_, 298 K): δ 9.54 (m, 1H, CH C-ring qn), 8.60 (d, ^3^*J*_H–H_ = 4.4, 1H, CH N-ring qn), 8.50 (m, 1H, CH C-ring qn), 8.09 (m, 2H, CH N-ring qn), 7.51 (m, 2H, CH C-ring qn), 7.17 (m, 2H, CH-arom POP), 7.05 (dd, ^3^*J*_H–H_ = 7.2, ^4^*J*_H–H_ = 1.5, 2H, CH-arom POP), 6.84 (t, ^3^*J*_H–H_ = 7.5, 2H, CH-arom POP), 2.34 (m, 2H, PC*H*(CH_3_)_2_), 2.14 (m, 2H, PC*H*(CH_3_)_2_), 1.30 (s, 3H, CH_3_), 1.19 (s, 3H, CH_3_), 1.10 (dvt, ^3^*J*_H–H_ = 6.5, *N* = 13.6, 12H, PCH(C*H*_3_)_2_), 1.02 (dvt, ^3^*J*_H–H_ = 7.5, *N* = 14.7, 6H, PCH(C*H*_3_)_2_), 0.78 (dvt, ^3^*J*_H–H_ = 7.9, *N* = 15.7, 6H, PCH(C*H*_3_)_2_). ^13^C{^1^H}-apt NMR (75.48 MHz, benzene-*d*_6_, 298 K): δ 187.9 (dt, ^1^*J*_C–Rh_ = 43.0, ^2^*J*_C–P_ = 11.7, Rh–C qn), 156.0 (vt, *N* = 15.5, C-arom POP), 148.1 (s, C qn), 145.4 (s, CH N-ring qn), 140.8 (s, C qn), 138.9 (s, CH C-ring qn), 132.6 (s, CH N-ring qn), 131.2 (s, CH-arom POP), 131.0 (vt, *N* = 5.0, C-arom POP), 130.8 (s, CH C-ring qn), 127.9 (s, CH-arom POP, inferred from the HSQC spectrum), 127.3 (s, CH C-ring qn), 125.0 (vt, *N* = 16.4, C-arom POP), 124.3 (s, CH-arom POP), 121.5 (s, CH C-ring qn), 34.5 (s, C(*C*H_3_)_2_), 34.1 (s, *C*(CH_3_)_2_), 31.2 (s, C(*C*H_3_)_2_), 25.7 (dvt, ^2^*J*_C–Rh_ = 2.1, *N* = 18.4, P*C*H(CH_3_)_2_), 24.8 (dvt, ^2^*J*_C–Rh_ = 2.8, *N* = 19.3, P*C*H(CH_3_)_2_), 19.3 (vt, *N* = 6.9, PCH(*C*H_3_)_2_), 18.3 (m, PCH(*C*H_3_)_2_), 18.0 (s, PCH(*C*H_3_)_2_). ^31^P{^1^H} NMR (121.49 MHz, benzene-*d*_6_, 298 K): δ 38.5 (d, ^1^*J*_Rh–P_ = 171.1).

#### Spectroscopic Data for **9** Obtained from Figures S23–S25

^1^H NMR (300.13 MHz, benzene-*d*_6_, 298 K): δ 8.38 (d, ^3^*J*_H–H_ = 8.3, 1H, CH C-ring qn), 8.19 (d, ^3^*J*_H–H_ = 8.4, 1H, CH N-ring qn), 7.69 (d, ^3^*J*_H–H_ = 8.6, 1H, CH C-ring qn), 7.58 (m, 1H, CH C-ring qn), 7.44–7.32 (m, 3H, CH N-ring qn + 2 CH-arom POP), 7.27 (m, 1H, CH C-ring qn), 7.16 (d, ^3^*J*_H–H_ = 7.7, 2H, CH-arom POP), 6.97 (t, ^3^*J*_H–H_ = 7.6, 2H, CH-arom POP), 2.54 (m, 4H, PC*H*(CH_3_)_2_), 1.35 (s, 6H, CH_3_), 1.39–1.25 (m, 24H, PCH(C*H*_3_)_2_). ^31^P{^1^H} NMR (121.49 MHz, benzene-*d*_6_, 298 K): δ 37.7 (d, ^1^*J*_Rh–P_ = 184.7).

### Reaction of **1** with 8-Methylquinoline

A solution of **1** (200 mg, 0.37 mmol) in *n*-octane (3 mL) was treated with 8-methylquinoline (50 μL, 0.37 mmol), and the resulting mixture was stirred at 80 °C for 48 h. After this time, the solution was evaporated to dryness to afford a brown residue. Addition of pentane (4 mL) afforded an orange solid that was washed with pentane (2 × 2 mL) and dried in vacuo. Yield: 91 mg (36%). The reaction is quantitative, but the isolated yield is low due to the high solubility in pentane. The ^31^P{^1^H} NMR spectra of the crude solution show that the ratio of Rh(κ^1^-C^4^-quinolinyl-8-Me){κ^3^-P,O,P-[xant(P^i^Pr_2_)_2_]} (**10**): Rh(κ^1^-C^2^-quinolinyl-8-Me){κ^3^-P,O,P-[xant(P^i^Pr_2_)_2_]} (**11**) is 1:1. Upon isolation and due to their different solubility in pentane, the ratio of **10**:**11** changes to 35:65. Anal. Calcd. for C_37_H_48_NOP_2_Rh: C, 64.63; H, 7.04; N, 2.04. Found; 64.78; H, 7.00; N, 2.27. HRMS (electrospray, *m*/*z*): calcd for C_37_H_49_NOP_2_Rh [M + H]^+^, 688.2339; found, 688.2325. IR (cm^–1^): ν(C=N) 1580 (m), ν(C–O–C) 1193 (m).

#### Spectroscopic Data for **10**

^1^H NMR (500.13 MHz, benzene-*d*_6_, 298 K): δ 9.56 (m, 1H, CH C-ring qn), 8.72 (d, ^3^*J*_H–H_ = 4.4, 1H, CH N-ring qn), 8.21 (broad s, 1H, CH N-ring qn), 7.65–7.54 (m, 1H, CH C-ring qn), 7.31–7.22 (m, 3H, 2H CH-arom POP + 1H CH C-ring qn), 7.20–7.13 (m, 2H, CH-arom POP), 7.00–6.92 (m, 2H, CH-arom POP), 3.27 (s, 3H, CH_3_ qn), 2.45 (m, 2H, PC*H*(CH_3_)_2_), 2.26 (m, 2H, PC*H*(CH_3_)_2_), 1.46–1.12 (m, 24H, 6H CH_3_ + 18H PCH(C*H*_3_)_2_), 0.90 (dvt, ^3^*J*_H–H_ = 8.3, *N* = 15.6, 6H, PCH(C*H*_3_)_2_). ^13^C{^1^H}-apt (100.62 MHz, benzene-*d*_6_, 298 K): δ 188.3 (dt, ^1^*J*_C–Rh_ = 41.5, ^2^*J*_C–P_ = 12.5, Rh–C qn), 156.0 (vt, *N* = 15.2, C-arom POP), 146.8 (s, C qn), 144.2 (s, CH N-ring qn), 140.5 (s, C qn), 137.5 (s, C qn), 137.3 (s, CH C-ring qn), 132.6 (s, CH N-ring qn), 131.1 (s, CH-arom POP), 130.9 (s, C-arom POP), 127.8 (s, CH-arom POP, inferred from the HSQC spectrum), 127.5 (s, CH C-ring qn, inferred from the HSQC spectrum), 125.0 (vt, *N* = 15.6, C-arom POP), 124.3 (s, CH-arom POP), 121.3 (s, CH C-ring qn), 34.5 (s, C(*C*H_3_)_2_), 34.1 (s, *C*(CH_3_)_2_), 31.2 (s, C(*C*H_3_)_2_), 26.0–25.4 (m, P*C*H(CH_3_)_2_), 24.8 (vt, *N* = 18.7, P*C*H(CH_3_)_2_), 19.4 (s, CH_3_ qn), 19.3, 18.5, 18.4, 18.0 (all s, PCH(*C*H_3_)_2_). ^31^P{^1^H} NMR (161.98 MHz, benzene-*d*_6_, 298 K): δ 38.1 (d, ^1^*J*_Rh–P_ = 171.8).

#### Spectroscopic Data for **11**

^1^H NMR (500.13 MHz, benzene-*d*_6_, 298 K): δ 8.16 (d, ^3^*J*_H–H_ = 8.4, 1H, CH N-ring qn), 7.65–7.54 (m, 2H, CH C-ring qn), 7.40 (m, 2H, CH-arom POP), 7.36 (d, ^3^*J*_H–H_ = 8.4, 1H, CH N-ring qn), 7.31–7.22 (m, 1H, CH qn), 7.20–7.13 (m, 2H, CH-arom POP), 7.00–6.92 (m, 2H, CH-arom POP), 3.25 (s, 3H, CH_3_ qn), 2.51 (m, 4H, PC*H*(CH_3_)_2_), 1.46–1.12 (m, 30H, 6 CH_3_, 24 PCH(C*H*_3_)_2_). ^13^C{^1^H}-apt NMR (100.62 MHz, benzene-*d*_6_, 298 K): δ 197.4 (dt, ^1^*J*_C–Rh_ = 43.1, ^2^*J*_C–P_ = 10.9, Rh–C qn), 156.4 (vt, *N* = 15.7, C-arom POP), 147.6 (s, C qn), 134.9 (s, CH N-ring qn), 134.7 (s, C qn), 131.3 (s, CH-arom POP), 131.2 (s, C-arom POP), 127.5 (s, CH qn, inferred from the HSQC spectrum), 127.4 (s, CH-arom POP), 126.5 (s, CH qn), 126.0 (vt, *N* = 15.6, C-arom POP), 125.2 (s, CH N-ring qn), 125.0 (s, C qn), 124.1 (s, CH-arom POP), 121.2 (s, CH qn), 34.3 (s, *C*(CH_3_)_2_), 32.2 (s, C(*C*H_3_)_2_), 26.0–25.4 (m, P*C*H(CH_3_)_2_), 19.6 (vt, *N* = 7.8, PCH(*C*H_3_)_2_), 19.2 (s, CH_3_ qn), 18.9 (s, PCH(*C*H_3_)_2_). ^31^P{^1^H} NMR (161.98 MHz, benzene-*d*_6_, 298 K): δ 37.3 (d, ^1^*J*_Rh–P_ = 184.8).

### Reaction of **1** with 3-Methoxyquinoline: Preparation of Rh(κ^1^-C^2^-Quinolinyl-3-OMe){κ^3^-P,O,P-[xant(P^i^Pr_2_)_2_]} (**12**)

A solution of **1** (200 mg, 0.37 mmol) in *n*-octane (3 mL) was treated with 3-methoxyquinoline (53 μL, 0.37 mmol), and the resulting mixture was stirred at 80 °C for 72 h. After this time, the solution was evaporated to dryness to afford a brown residue. Addition of pentane (4 mL) afforded an orange solid that was washed with pentane (2 × 2 mL) and dried in vacuo. Yield: 126 mg (49%). The reaction is quantitative, but the isolated yield is low due to its high solubility in pentane. Anal. Calcd. for C_37_H_48_NO_2_P_2_Rh: C, 63.16; H, 6.88; N, 2.04. Found; C, 63.38; H, 6.62; N, 2.21. HRMS (electrospray, *m*/*z*): calcd for C_37_H_48_NO_2_P_2_Rh [M]^+^, 703.2210; found, 703.2226. IR (cm^–1^): ν(C=N) 1580 (m), ν(C–O–C) 1090 (s), 1017(s). ^1^H NMR (400.13 MHz, benzene-*d*_6_, 343 K): δ 8.21 (d, ^3^*J*_H–H_ = 8.1, 1H, CH C-ring qn), 7.63 (d, ^3^*J*_H–H_ = 7.8, 1H, CH C-ring qn), 7.40 (m, 1H, CH C-ring qn), 7.34 (m, 2H, CH-arom POP), 7.21 (m, 1H, CH C-ring qn), 7.12 (d, ^3^*J*_H–H_ = 7.7, 2H, CH-arom POP), 6.90 (t, ^3^*J*_H–H_ = 7.5, 2H, CH-arom POP), 6.53 (s, 1H, CH N-ring qn), 3.70 (s, 3H, CH_3_ qn), 2.39 (m, 4H, PC*H*(CH_3_)_2_), 1.31 (s, 6H, CH_3_), 1.24 (dvt, ^3^*J*_H–H_ = 7.1, *N* = 14.1, 12H, PCH(C*H*_3_)_2_), 1.15 (dvt, ^3^*J*_H–H_ = 7.6, *N* = 15.9, 12H, PCH(C*H*_3_)_2_). ^13^C{^1^H}-apt NMR (100.62 MHz, benzene-*d*_6_, 343 K): δ 192.9 (dt, ^1^*J*_C–Rh_ = 45.1, ^2^*J*_C–P_ = 11.2, Rh–C qn), 157.7 (s, C–CH_3_ qn), 156.8 (vt, *N* = 16.0, C-arom POP), 145.2 (d, ^3^*J*_C–Rh_ = 3.6, C qn), 131.5 (s, C-arom POP), 131.2 (s, CH-arom POP), 128.0 (s, CH C-ring qn, inferred from the HSQC spectrum), 127.1 (s, CH-arom POP), 126.8 (s, C qn), 126.7 (s, CH C-ring qn), 126.2 (s, C-arom POP), 124.9 (s, CH C-ring qn), 124.1 (s, CH-arom POP), 121.6 (s, CH C-ring qn), 101.8 (s, CH, qn N-ring), 54.1 (s, OCH_3_), 34.5 (s, *C*(CH_3_)_2_), 31.9 (s, C(*C*H_3_)_2_, inferred from the HSQC spectrum), 26.3 (vt, *N* = 14.5, P*C*H(CH_3_)_2_), 19.2 (vt, *N* = 8.9, PCH(*C*H_3_)_2_), 18.9 (s, PCH(*C*H_3_)_2_). ^31^P{^1^H} NMR (161.98 MHz, benzene-*d*_6_, 343 K): δ 38.6 (d, ^1^*J*_Rh–P_ = 183.0).

### Reaction of **1** with 3-(Trifluoromethyl)quinoline

A solution of **1** (200 mg, 0.37 mmol) in *n*-octane (3 mL) was treated with 3-(trifluoromethyl)quinoline (72.1 mg, 0.37 mmol), and the resulting mixture was stirred at 80 °C for 5 days. After this time, the ^31^P{^1^H} NMR spectra of the crude solution show a mixture of derivatives Rh(κ^1^-C^2^-quinolinyl-3-CF_3_){κ^3^-P,O,P-[xant(P^i^Pr_2_)_2_]} (**13**; δ 35.3, d, ^1^*J*_Rh–P_ = 183.9 Hz; 25%), Rh(κ^1^-C^4^-quinolinyl-3-CF_3_){κ^3^-P,O,P-[xant(P^i^Pr_2_)_2_]} (**14**; δ 37.5, d, ^1^*J*_Rh–P_ = 174.6 Hz; 14%), Rh(κ^1^-C^6^-quinolinyl-3-CF_3_){κ^3^-P,O,P-[xant(P^i^Pr_2_)_2_]} (**15**; δ 37.8, d, ^1^*J*_Rh–P_ = 172.8 Hz; 25%), and Rh(κ^1^-C^7^-quinolinyl-3-CF_3_){κ^3^-P,O,P-[xant(P^i^Pr_2_)_2_]} (**16**; δ 38.0, d, ^1^*J*_Rh–P_ = 173.7 Hz; 36%). The solution was evaporated to dryness to afford a brown residue. Addition of pentane (4 mL) afforded a red solid that was washed with pentane (2 × 2 mL) and dried in vacuo. Yield: 53 mg (20%). ^1^H and ^31^P{^1^H} NMR spectra of the red solid show only complexes **15** and **16** in a ratio of 1:2.5. Anal. Calcd. for C_37_H_45_F_3_NOP_2_Rh: C, 59.92; H, 6.12; N, 1.89. Found: 60.31; H, 6.43; N, 2.08. HRMS (electrospray, *m*/*z*): calcd for C_37_H_46_F_3_NOP_2_Rh [M + H]^+^, 742.2056; found, 742.2058. IR (cm^–1^): ν(C=N) 1577 (m), ν(C–O–C) 1151 (m).

#### Spectroscopic Data of Complex **15**

^1^H NMR (400.13 MHz, benzene-*d*_6_, 298 K): δ 9.05 (broad singlet, 1H, CH N-ring qn), 8.66 (d, ^3^*J*_H–H_ = 8.4, 1H, CH C-ring qn), 8.28 (s, 1H, CH C-ring qn), 8.15 (s, 1H, CH C-ring qn), 8.12 (d, ^3^*J*_H–H_ = 8.5, CH C-ring qn), 7.27–7.18 (m, 2H, 2 CH-arom POP), 7.10–7.01 (m, 2H, CH-arom POP), 6.91–6.80 (m, 2H, CH-arom POP), 2.31 (m, 4H, PC*H*(CH_3_)_2_), 1.24 (s, 6H, CH_3_), 1.17–0.99 (m, 24H, PCH(C*H*_3_)_2_). ^13^C{^1^H}-apt NMR (100.62 MHz, benzene-*d*_6_, 298 K): δ 156.0 (m, C-arom POP), 147.6 (s, C qn), 146.1 (s, CH C-ring qn), 140.9 (s, CH N-ring qn), 135.2 (s, CH C-ring qn), 131.3 (s, CH-arom POP), 130.9 (m, C-arom POP), 129.8 (s, CH N-ring qn), 128.2 (s, CH-arom POP, inferred from the HSQC spectrum), 125.7 (s, C qn), 125.0 (s, C-arom POP), 124.3 (s, CH-arom POP), 123.7 (s, CH C-ring qn), 123.1 (q, ^1^*J*_C–F_ = 279.2, *C*F_3_ qn), 118.7 (q, ^2^*J*_C–F_ = 31.8, *C*–CF_3_), 34.1 (s, *C*(CH_3_)_2_), 33.0 (s, C(*C*H_3_)_2_), 25.4 (m, P*C*H(CH_3_)_2_), 19.3 (s, PCH(*C*H_3_)_2_), 18.5 (s, PCH(*C*H_3_)_2_); the signal for the Rh–C atom was not observed. ^31^P{^1^H} NMR (161.98 MHz, benzene-*d*_6_, 298 K): δ 37.8 (d, ^1^*J*_Rh–P_ = 172.8).

#### Spectroscopic Data of Complex **16**

^1^H NMR (400.13 MHz, benzene-*d*_6_, 298 K): δ 9.14 (broad singlet, 1H, CH N-ring qn), 8.96 (s, 1H, CH C-ring qn), 8.52 (d, ^3^*J*_H–H_ = 8.1, 1H, CH C-ring qn), 8.05 (s, H, CH N-ring qn), 7.27–7.18 (m, 3H, 1H CH C-ring qn, 2H CH-arom POP), 7.10–7.01 (m, 2H, CH-arom POP), 6.91–6.80 (m, 2H, CH-arom POP), 2.31 (m, 4H, PC*H*(CH_3_)_2_), 1.23 (s, 6H, CH_3_), 1.17–0.99 (m, 24H, PCH(C*H*_3_)_2_). ^13^C{^1^H}-apt NMR (100.62 MHz, benzene-*d*_6_, 298 K): δ 179.7 (dt, ^1^*J*_C–Rh_ = 40.4, ^2^*J*_C–P_ = 12.5, Rh–C qn), 156.0 (m, C-arom POP), 147.9 (s, C qn), 144.8 (s, CH N-ring qn), 142.3 (s, CH C-ring qn), 137.7 (s, CH C-ring qn), 133.3 (s, CH N-ring qn), 131.3 (s, CH-arom POP), 130.9 (m, C-arom POP), 128.2 (s, CH-arom POP, inferred from the HSQC spectrum), 125.0 (m, C-arom POP), 124.6 (s, C qn), 124.3 (s, CH-arom POP), 123.1 (q, ^1^*J*_C–F_ = 279.2, *C*F_3_ qn), 121.2 (s, CH C-ring qn), 118.7 (q, ^2^*J*_C–F_ = 31.8, *C*–CF_3_), 34.1 (s, *C*(CH_3_)_2_), 33.0 (s, C(*C*H_3_)_2_), 25.4 (m, P*C*H(CH_3_)_2_), 19.3 (s, PCH(*C*H_3_)_2_), 18.5 (s, PCH(*C*H_3_)_2_). ^31^P{^1^H} NMR (161.98 MHz, benzene-*d*_6_, 298 K): δ 38.0 (d, ^1^*J*_Rh–P_ = 173.7).

## Supporting Information Available

General information for the Experimental Section, structural analysis, and NMR spectra (PDF)
