# Supplementary material for: Rhodium-Promoted C–H Bond Activation of Quinoline, Methylquinolines, and Related Mono-Substituted Quinolines
Source: Organometallics. 2022 Aug 11;41(16):2317–26. doi: 10.1021/acs.organomet.2c00270 (PMC9969481; doi:10.1021/acs.organomet.2c00270)
Supplement: Supplementary file 1 — om2c00270_si_001.pdf [file om2c00270_si_001.pdf]

SUPPORTING INFORMATION

Rhodium-Promoted C-H Bond Activation of Quinoline, Methylquinolines and Related Mono-Substituted Quinolines

Laura A. de las Heras, Miguel A. Esteruelas,* Montserrat Oliván, and Enrique Oñate

Departamento de Química Inorgánica – Instituto de Síntesis Química y Catálisis Homogénea (ISQCH) – Centro de Innovación en Química Avanzada (ORFEO-CINQA), Universidad de Zaragoza – CSIC, 50009 Zaragoza, Spain

* e-mail: maester@unizar.es

Contents:

- General Information.	S2
- Structural Analysis of Complexes **2**, **3**, **4**, **5**, **6**, **8**, **11**, **12**, **13**, and **15-16**	S3
- NMR spectra	S8

• General Information

All reactions were carried out with exclusion of air using Schlenk-tube techniques or in a drybox. Pentane was obtained oxygen- and water-free from an MBraun solvent purification apparatus, while *n*-octane was dried and distilled under argon prior to use. Quinolines (except 3-(trifluorometyl)quinoline, that is solid and was used as received) were distillated in a Kugelrohr glass over prior to use. ^{1}H, $^{13}C\{^{1}H\}$, and $^{31}P\{^{1}H\}$ NMR spectra were recorded on Bruker 300 ARX, Bruker Avance 300 MHz, Bruker Avance 400 MHz, or Bruker Avance 500 MHz instruments. Chemical shifts (expressed in ppm) are referenced to residual solvent peaks (^{1}H, $^{13}C\{^{1}H\}$) or external 85% H_3PO_4 ($^{31}P\{^{1}H\}$). Coupling constant J and N ($N = J_{P\text{-}H} + J_{P'\text{-}H}$ for ^{1}H and $N = J_{P\text{-}C} + J_{P'\text{-}C}$ for $^{13}C\{^{1}H\}$) are given in hertz. Attenuated total reflection infrared spectra (ATR-IR) of solid samples were run on a PerkinElmer Spectrum 100 FT-IR spectrometer. C, H, and N analyses were carried out in a PerkinElmer 2400 CHNS/O analyzer. High-resolution electrospray mass spectra were acquired using a MicroTOF-Q hybrid quadrupole time-of-flight spectrometer (Bruker Daltonics, Bremen, Germany). RhH{κ^3-P,O,P-[xant(P^iPr_2)$_2$]} (**1**)[1] was prepared by the published method.

• Structural Analysis of Complexes 2, 3, 4, 5, 6, 8, 11, 12, 13, and 15-16.

X-ray data were collected on a APEX CCD (**3**, **11**) and D8 Venture Bruker diffractometers (Mo radiation, λ = 0.71073 Å). The crystals were cooled with a nitrogen flow from Oxford Cryosystems systems. Data were corrected for absorption by using a multiscan method applied with the SADABS program.[2] The structures were solved by Patterson or direct methods and refined by full-matrix least squares on F^2 with SHELXL2016 or SHELXL2019,[3] including isotropic and subsequently anisotropic displacement parameters. The hydrogen atoms were observed in the last Fourier Maps or calculated, and refined freely or using a restricted riding model.

Complex **13** crystallizes in a pseudomerohedral twin. A monoclinic crystal structure which has $\beta \simeq 90°$ has a lattice with, at least approximately, the *mmm* symmetry characteristic of the orthorhombic crystal family. The crystal was solved and refined in the monoclinic $P2_1/c$ space group with the twin matrix (1 0 0 0 -1 0 0 0 -1) and a BASF parameter of 0.46561.

Complexes **15** and **16** co-crystallize in the same crystal given rise also to a pseudomerohedral twin. A monoclinic crystal structure which $\beta \simeq 90°$. The crystal was solved and refined in the monoclinic $P2_1/c$ space group with the twin matrix (1 0 0 0 -1 0 0 0 -1) and a BASF parameter of 0.49965, as well as in the orthorhombic Pnma space group with the xant(P^iPr_2)$_2$ ligand disordered in two moieties (occupancy 0.5 each). The result is better in the group with higher symmetry, although in both cases geometric and displacement parameter constraints had to be implemented to achieve a reasonable geometry. Furthermore, the trifluoromethyl group of the activated 3-(trifluoromethyl)quinoline ligand was observed disordered in two positions, indicating unequivocally that the crystal is formed by a co-crystallization of complexes **15** and **16**.

Crystal data for **2** (CCDC 2175435): $C_{37}H_{48}NOP_2Rh$, M_W 687.61, orange, irregular block (0.224 x 0.080 x 0.045 mm^3), orthorhombic, space group $Pmn2_1$, *a*: 14.4971(7) Å, *b*: 8.1708(5) Å, *c*: 14.5331(8) Å, V = 1721.49(16) Å^3, Z = 2, Z' = 0.5, D_{calc}: 1.327 g cm^{-3}, F(000): 720, T = 100(2) K, μ 0.618 mm^{-1}. 19753 measured reflections (2θ: 3-57°, ω scans 0.3°), 5221 unique (R_{int} = 0.0416); min./max. transm. factors 0.713/0.862. Final agreement factors were R^1 = 0.0778 (4287 observed reflections, $I > 2\sigma(I)$) and wR^2 =

0.2044; Flack parameter 0.41(9); data/restraints/parameters 5221/17/221; GoF = 1.095. Largest peak and hole 3.615 (close to Rh atoms) and -1.206 e/ Å^3.

Crystal data for **3** (CCDC 2175441): $C_{37}H_{48}NOP_2Rh$, M_W 687.61, orange, irregular block (0.224 x 0.162 x 0.059 mm^3), orthorhombic, space group Pmn2$_1$, *a*: 14.2273(11) Å, *b*: 15.6939(12) Å, *c*: 7.3895(6) Å, V = 1649.9(2) Å^3, Z = 2, Z' = 0.5, D_{calc}: 1.384 g cm^{-3}, F(000): 720, T = 100(2) K, μ 0.645 mm^{-1}. 23749 measured reflections (2θ: 3-57°, ω scans 0.3^o), 4145 unique (R_{int} = 0.0451); min./max. transm. factors 0.781/0.862. Final agreement factors were R^1 = 0.0295 (3770 observed reflections, I > 2σ(I)) and wR^2 = 0.0957; Flack parameter 0.08(4); data/restraints/parameters 4145/1/219; GoF = 0.774. Largest peak and hole 0.428 (close to Rh atoms) and -0.749 e/ Å^3.

Crystal data for **4** (CCDC 2175436): $C_{37}H_{48}NOP_2Rh$, M_W 687.61, orange, irregular block (0.170 x 0.100 x 0.080 mm^3), orthorhombic, space group Pmn2$_1$, *a*: 14.4094(5) Å, *b*: 8.0370(3) Å, *c*: 14.7913(5) Å, V = 1712.96(10) Å^3, Z = 2, Z' = 0.5, D_{calc}: 1.333 g cm^{-3}, F(000): 720, T = 100(2) K, μ 0.621 mm^{-1}. 32739 measured reflections (2θ: 3-57°, ω scans 0.3^o), 3484 unique (R_{int} = 0.0334); min./max. transm. factors 0.777/0.862. Final agreement factors were R^1 = 0.0353 (3448 observed reflections, I > 2σ(I)) and wR^2 = 0.0979; Flack parameter 0.05(5); data/restraints/parameters 3484/7/221; GoF = 1.057. Largest peak and hole 1.683 (close to Rh atoms) and -0.471 e/ Å^3.

Crystal data for **5** (CCDC 2175437): $C_{37}H_{48}NOP_2Rh$, M_W 687.61, orange, plate (0.150 x 0.140 x 0.072 mm^3), triclinic, space group P-1, *a*: 11.5427(5) Å, *b*: 12.1709(6) Å, *c*: 13.8877(7) Å, *α*: 107.2161(16)°, *β*: 92.1728(16)°, *γ*: 111.5982(14)°, V = 1708.90(14) Å^3, Z = 2, Z' = 1, D_{calc}: 1.336 g cm^{-3}, F(000): 720, T = 100(2) K, μ 0.622 mm^{-1}. 58985 measured reflections (2θ: 3-57°, ω scans 0.3^o), 10324 unique (R_{int} = 0.0294); min./max. transm. factors 0.832/0.862. Final agreement factors were R^1 = 0.0281 (9928 observed reflections, I > 2σ(I)) and wR^2 = 0.0717; data/restraints/parameters 10324/28/390; GoF = 1.058. Largest peak and hole 1.419 (close to Rh atoms) and -0.614 e/ Å^3.

Crystal data for **6** (CCDC 2175442): $C_{37}H_{48}NOP_2Rh$, 2 x C_6H_6, M_W 687.61, yellow, irregular block (0.2 x 0.2 x 0.04 mm^3), triclinic, space group P-1, *a*: 11.3990(3) Å, *b*: 12.0760(4) Å, *c*: 17.7569(5) Å, *α*: 99.7560(10)°, *β*: 102.8960(10)°, *γ*: 109.9000(10)°, V = 2158.58(11) Å^3, Z = 2, Z' = 1, D_{calc}: 1.298 g cm^{-3}, F(000): 888, T = 100(2) K, μ 0.506 mm^{-1}. 64830 measured reflections (2θ: 3-57°, ω scans 0.3^o), 10693 unique (R_{int} =

0.0281); min./max. transm. factors 0.829/0.862. Final agreement factors were R^1 = 0.0202 (10246 observed reflections, I > 2σ(I)) and wR^2 = 0.0537; data/restraints/parameters 10693/0/498; GoF = 1.022. Largest peak and hole 0.483 (close to Rh atoms) and -0.453 e/ Å^3.

Crystal data for **8** (CCDC 2175438): $C_{36}H_{46}NOP_2Rh$, 3 x C_6H_6, M_W 907.91, yellow, irregular block (0.150 x 0.115 x 0.105 mm^3), triclinic, space group P-1, *a*: 10.3581(4) Å, *b*: 11.6486(3) Å, *c*: 19.8469(7) Å, *α*: 92.1735(11)°, *β*: 96.6412(12)°, *γ*: 103.9029(10)°, *V* = 2303.55(13) Å^3, *Z* = 2, *Z'* = 1, D_{calc}: 1.309 g cm^{-3}, F(000): 956, T = 100(2) K, μ 0.480 mm^{-1}. 64972 measured reflections (2θ: 3-57°, ω scans 0.3°), 8572 unique (R_{int} = 0.0295); min./max. transm. factors 0.827/0.862. Final agreement factors were R^1 = 0.0296 (8404 observed reflections, I > 2σ(I)) and wR^2 = 0.0674; data/restraints/parameters 8572/0/542; GoF = 1.069. Largest peak and hole 2.454 (close to Rh atoms) and -1.340 e/ Å^3.

Crystal data for **11** (CCDC 2175439): $C_{37}H_{48}NOP_2Rh$, M_W 687.61, red, irregular block (0.196 x 0.170 x 0.086 mm^3), orthorhombic, space group $Pmn2_1$, *a*: 14.467(3) Å, *b*: 8.0723(17) Å, *c*: 14.615(3) Å, *V* = 1706.9(6)Å ^{3}, *Z* = 2, *Z'* = 0.5, D_{calc}: 1.338 g cm^{-3}, F(000): 720, T = 100(2) K, μ 0.623 mm^{-1}. 20236 measured reflections (2θ: 3-57°, ω scans 0.3°), 4322 unique (R_{int} = 0.0507); min./max. transm. factors 0.694/0.862. Final agreement factors were R^1 = 0.0635 (3430 observed reflections, I > 2σ(I)) and wR^2 = 0.1878; Flack parameter -0.16(4); data/restraints/parameters 4322/8/215; GoF = 0.973. Largest peak and hole 3.018 (close to Rh atoms) and -0.600 e/ Å^3.

Crystal data for **12** (CCDC 2175440): $C_{37}H_{48}NO_2P_2Rh$, M_W 703.61, yellow, needle (0.202 x 0.072 x 0.04 mm^3), monoclinic, space group $P2_1/c$, *a*: 10.2761(15) Å, *b*: 16.0303(19) Å, *c*: 20.997(3) Å, *β*: 93.317(4)°, *V* = 3453.0(8) Å^3, *Z* = 4, *Z'* = 1, D_{calc}: 1.353 g cm^{-3}, F(000): 1472, T = 100(2) K, μ 0.620 mm^{-1}. 57776 measured reflections (2θ: 3-57°, ω scans 0.3°), 8526 unique (R_{int} = 0.0296); min./max. transm. factors 0.766/0.862. Final agreement factors were R^1 = 0.0275 (7855 observed reflections, I > 2σ(I)) and wR^2 = 0.0742; data/restraints/parameters 8526/5/399; GoF = 0.996. Largest peak and hole 1.132 (close to Rh atoms) and -0.422 e/ Å^3.

Crystal data for **13** (CCDC 2175444): $C_{37}H_{45}F_3NOP_2Rh$, M_W 741.59, orange, irregular block (0.256 x 0.156 x 0.065 mm^3), monoclinic, space group $P2_1/c$, *a*: 14.7443(7) Å, *b*:

14.5232(7) Å, *c*: 16.1036(8) Å, *β*: 89.9465(19)°, *V* = 3448.3(3) Å^3, *Z* = 4, *Z'* = 1, D_{calc}: 1.428 g cm^{-3}, F(000): 1536, T = 100(2) K, μ 0.635 mm^{-1}. 61723 measured reflections (2θ: 3-57°, ω scans 0.3^o), 8549 unique (R_{int} = 0.0509); min./max. transm. factors 0.655/0.862. Final agreement factors were R^1 = 0.0520 (7750 observed reflections, I > 2σ(I)) and wR^2 = 0.1486; data/restraints/parameters 8549/0/417; GoF = 1.049. Largest peak and hole 2.416 (close to Rh atoms) and -0.762 e/ Å^3.

Crystal data for **15-16** (CCDC 2175443): $C_{37}H_{45}F_3NOP_2Rh$, M_W 741.59, red, irregular block (0.155 x 0.130 x 0.077 mm^3), orthorhombic, space group Pnma, *a*: 13.364(4) Å, *b*: 14.408(3) Å, *c*: 18.129(5) Å, *V* = 3490.8(15) Å ^{3}, *Z* = 4, *Z'* = 0.5, D_{calc}: 1.411 g cm^{-3}, F(000): 1536, T = 100(2) K, μ 0.627 mm^{-1}. 157921 measured reflections (2θ: 3-57°, ω scans 0.3^o), 4470 unique (R_{int} = 0.0319); min./max. transm. factors 0.807/0.862. Final agreement factors were R^1 = 0.0595 (4246 observed reflections, I > 2σ(I)) and wR^2 = 0.1614; data/restraints/parameters 4470/227/ 389; GoF = 1.146. Largest peak and hole 1.970 (close to Rh atoms) and -1.742 e/ Å^3.

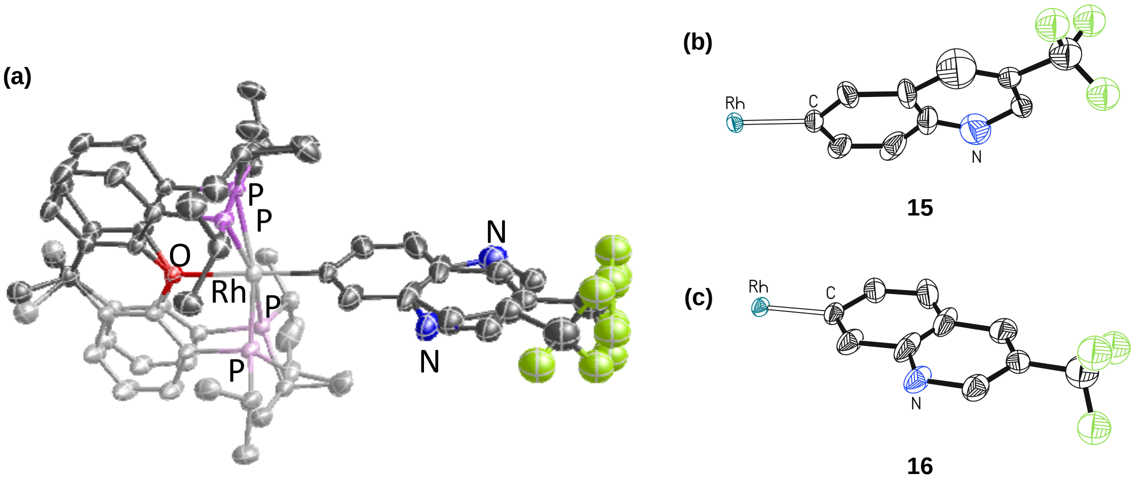

Figure S1. (a) Molecular structure of complexes **15-16**. (b) Detail of the structure showing the activation of position 6 of 3-(trifluoromethyl)quinoline (c) Detail of the structure showing the activation of position 7 of 3-(trifluoromethyl)quinoline. Hydrogen atoms are omitted for clarity.

References

(1) Esteruelas, M. A.; Martínez, A.; Oliván, M.; Vélez, A. A General Rhodium Catalyst for the Deuteration of Boranes and Hydrides of the Group 14 Elements. *J. Org. Chem.* **2020**, *85*, 15693-15698.

(2) Blessing, R. H. *Acta Crystallogr.* **1995**, *A51*, 33. SADABS: Area-detector absorption correction; Bruker- AXS, Madison, WI, 1996.

(3) SHELXL-2016/6. Sheldrick, G. M. *Acta Cryst.* **2008**, *A64*, 112-122.

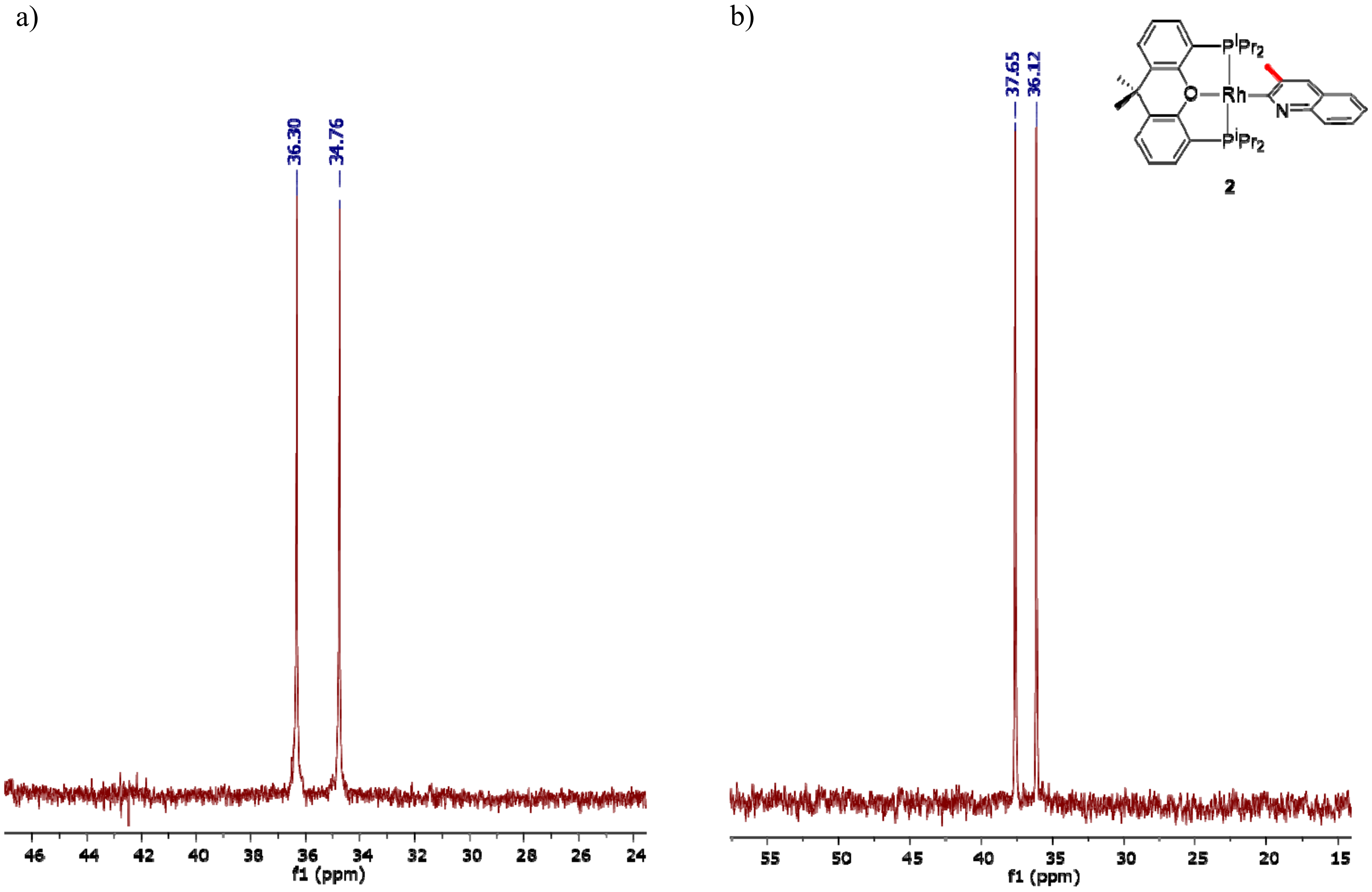

Figure S2. (a) $^{31}P\{^{1}H\}$ NMR spectrum (121.49 MHz, *n*-octane, 298 K) of the reaction of **1** with 3-methylquinoline after 48 h at 80 ºC. (b) $^{31}P\{^{1}H\}$ NMR spectrum (121.49 MHz, benzene-d_6, 298 K) of Rh(κ^1-C^2-quinolinyl-3-Me){κ^3-P,O,P-[xant(P^iPr_2)$_2$]} (**2**).

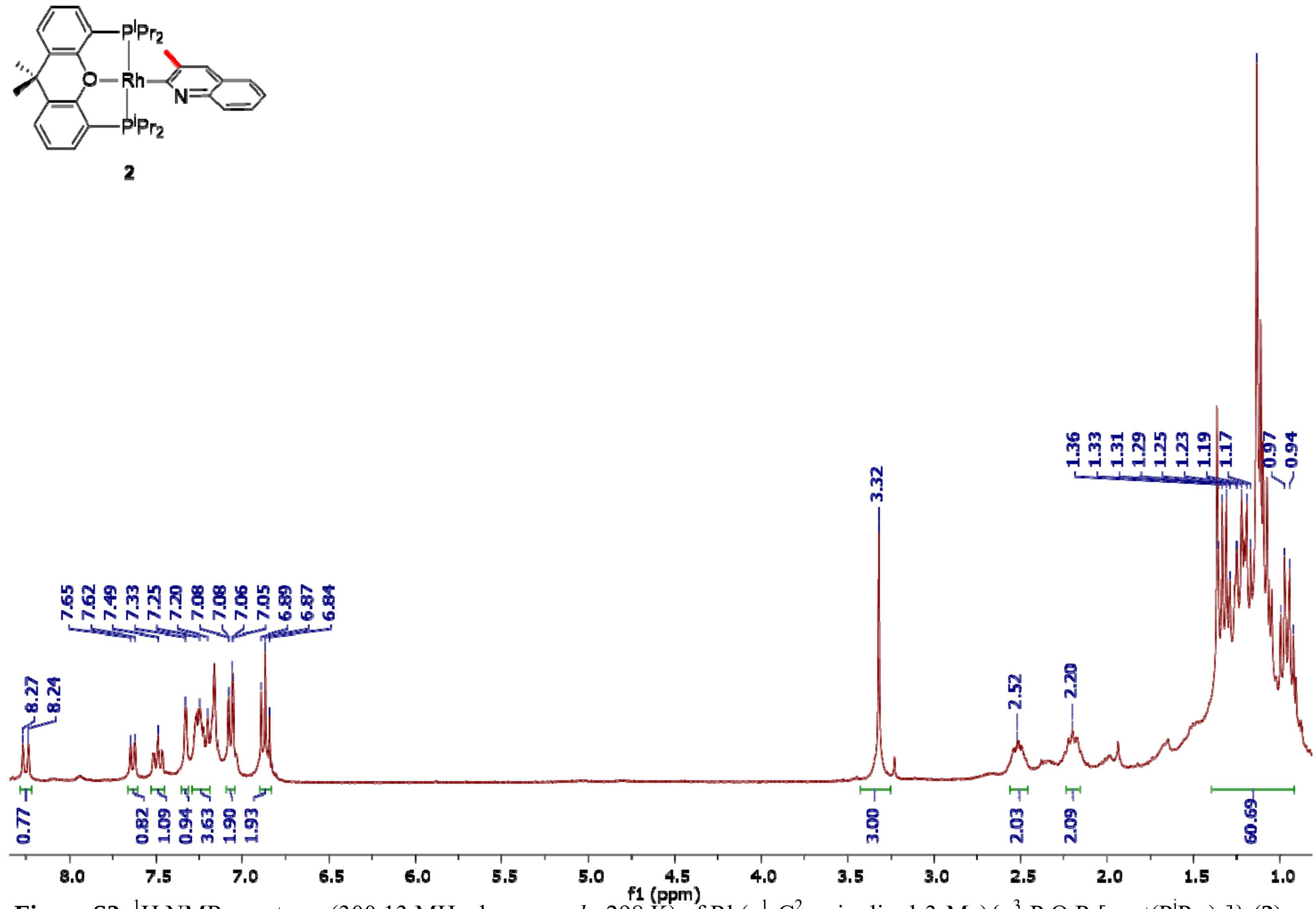

Figure S3. ^{1}H NMR spectrum (300.13 MHz, benzene-d_6, 298 K) of Rh(κ^1-C^2-quinolinyl-3-Me){κ^3-P,O,P-[xant(P^iPr_2)$_2$]} (**2**).

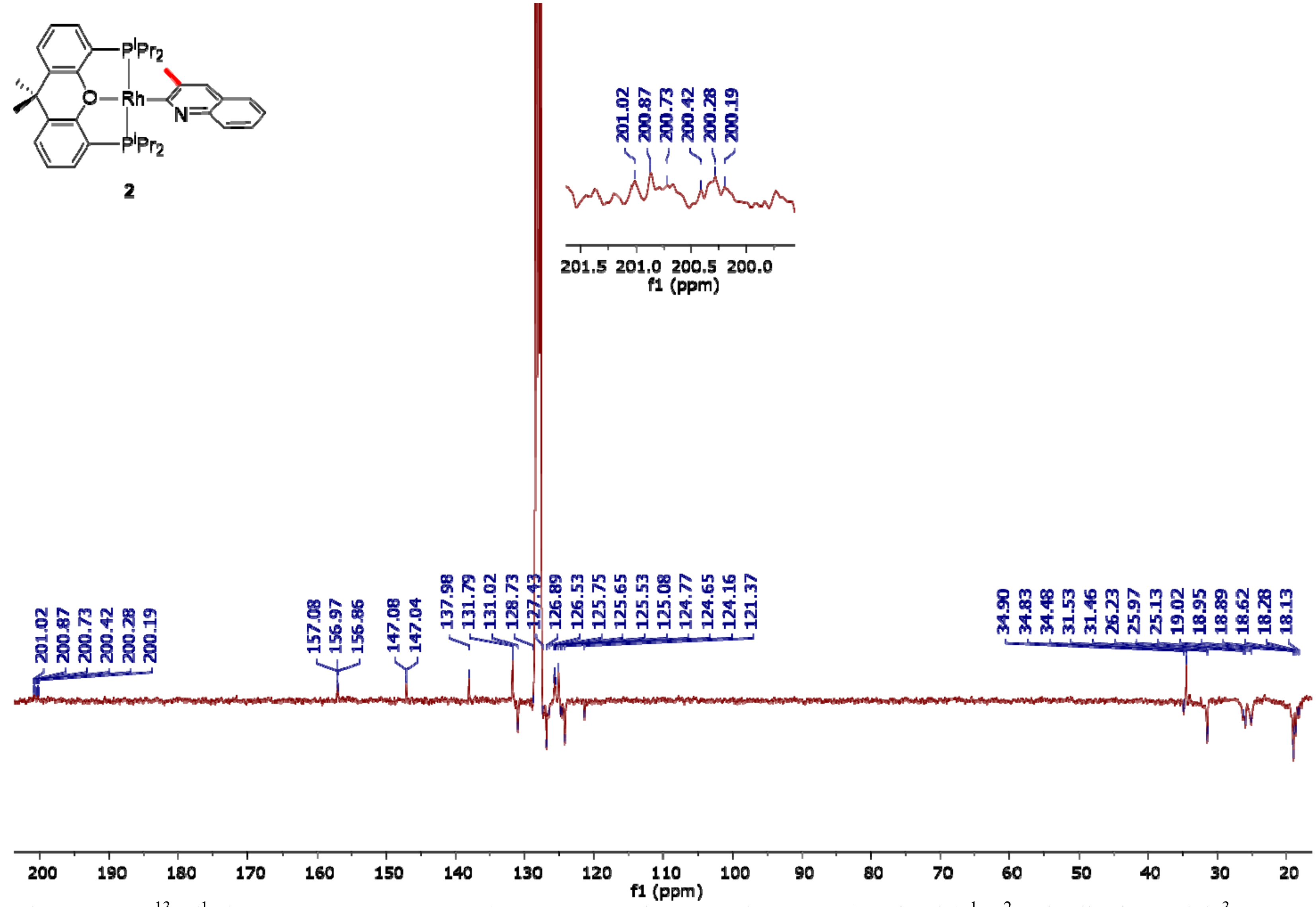

Figure S4. $^{13}C\{^1H\}$-apt NMR spectrum (75.48 MHz, benzene-d_6, 298 K) of Rh(κ^1-C^2-quinolinyl-3-Me){κ^3-P,O,P-[xant(P^{i}Pr$_2$)$_2$]} (**2**). APT spectra show CH and CH_3 negative while CH_2 and C are positive.

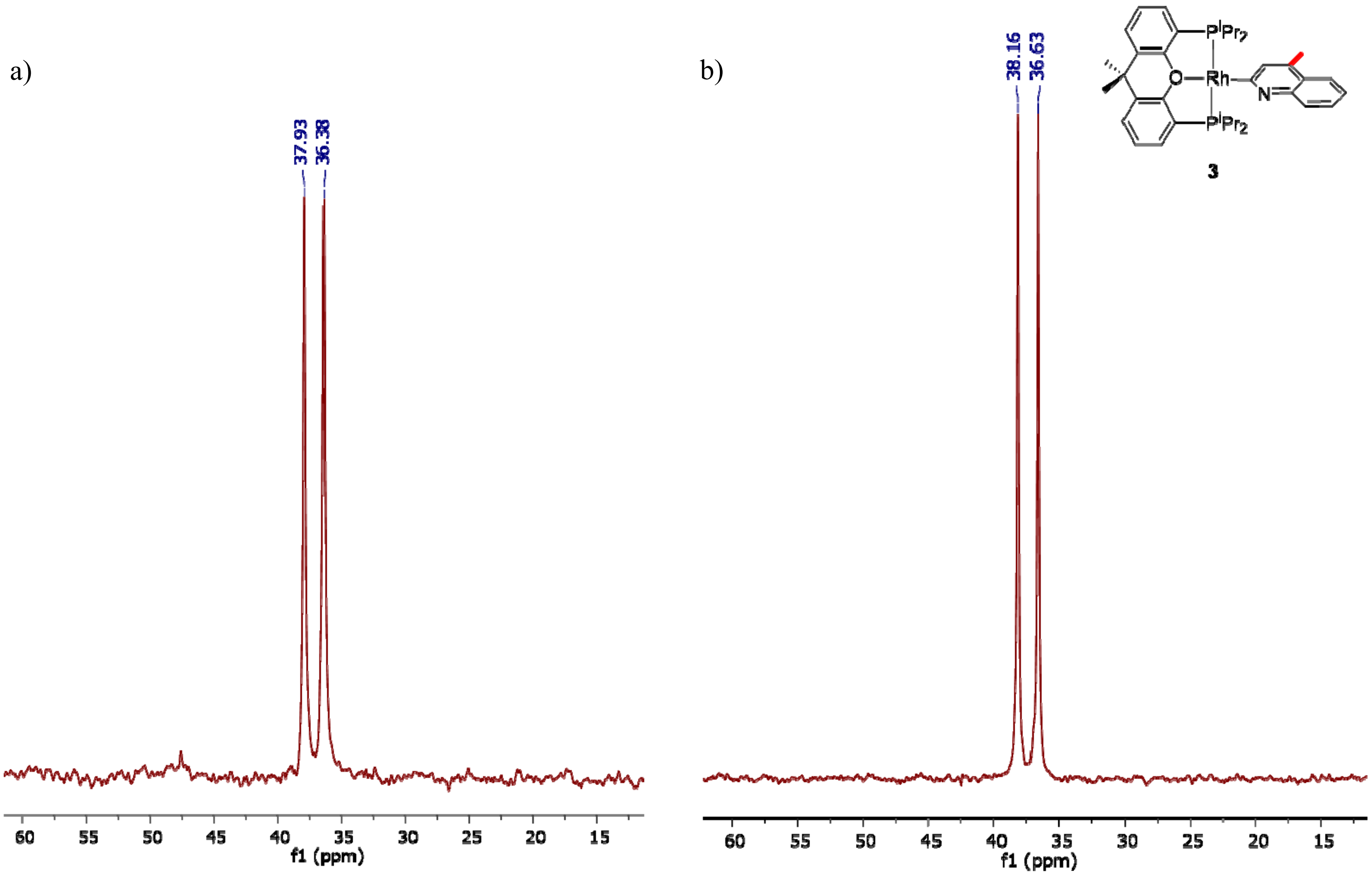

Figure S5. (a) $^{31}P\{^1H\}$ NMR spectrum (121.49 MHz, *n*-octane, 298 K) of the reaction of **1** with 4-methylquinoline after 48 h at 80 ºC. (b) $^{31}P\{^1H\}$ NMR spectrum (121.49 MHz, benzene-d_6, 298 K) of Rh(κ^1-C^2-quinolinyl-4-Me){κ^3-P,O,P-[xant(P^{i}Pr$_2$)$_2$]} (**3**).

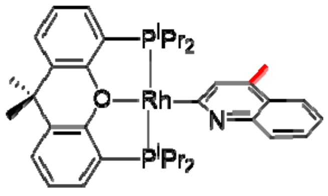

3

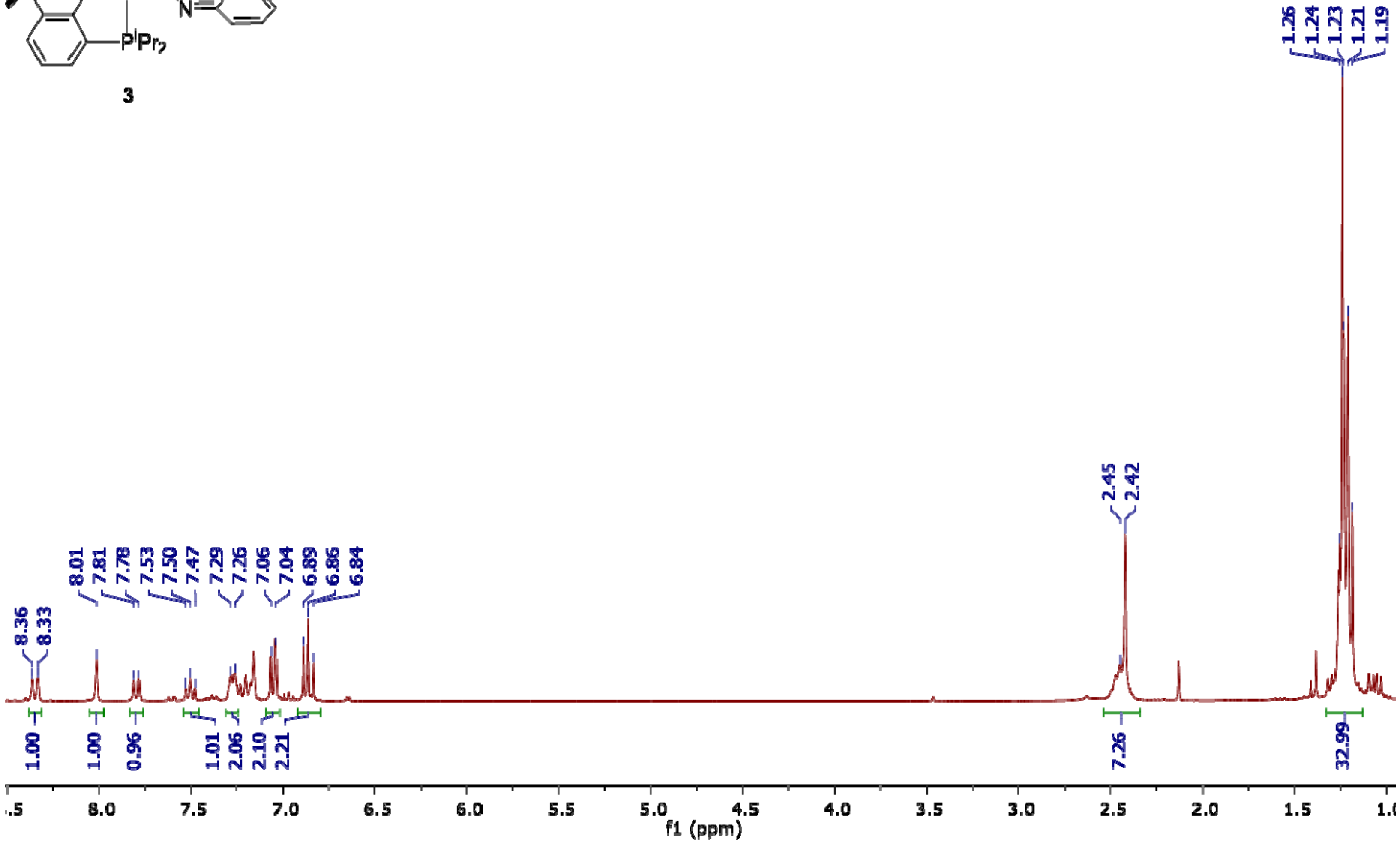

Figure S6. ^{1}H NMR spectrum (300.13 MHz, benzene-d_6, 298 K) of Rh(κ^1-C^2-quinolinyl-4-Me){κ^3-P,O,P-[xant(P^iPr_2)$_2$]} (**3**).

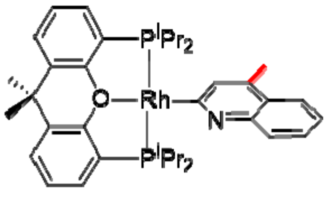

3

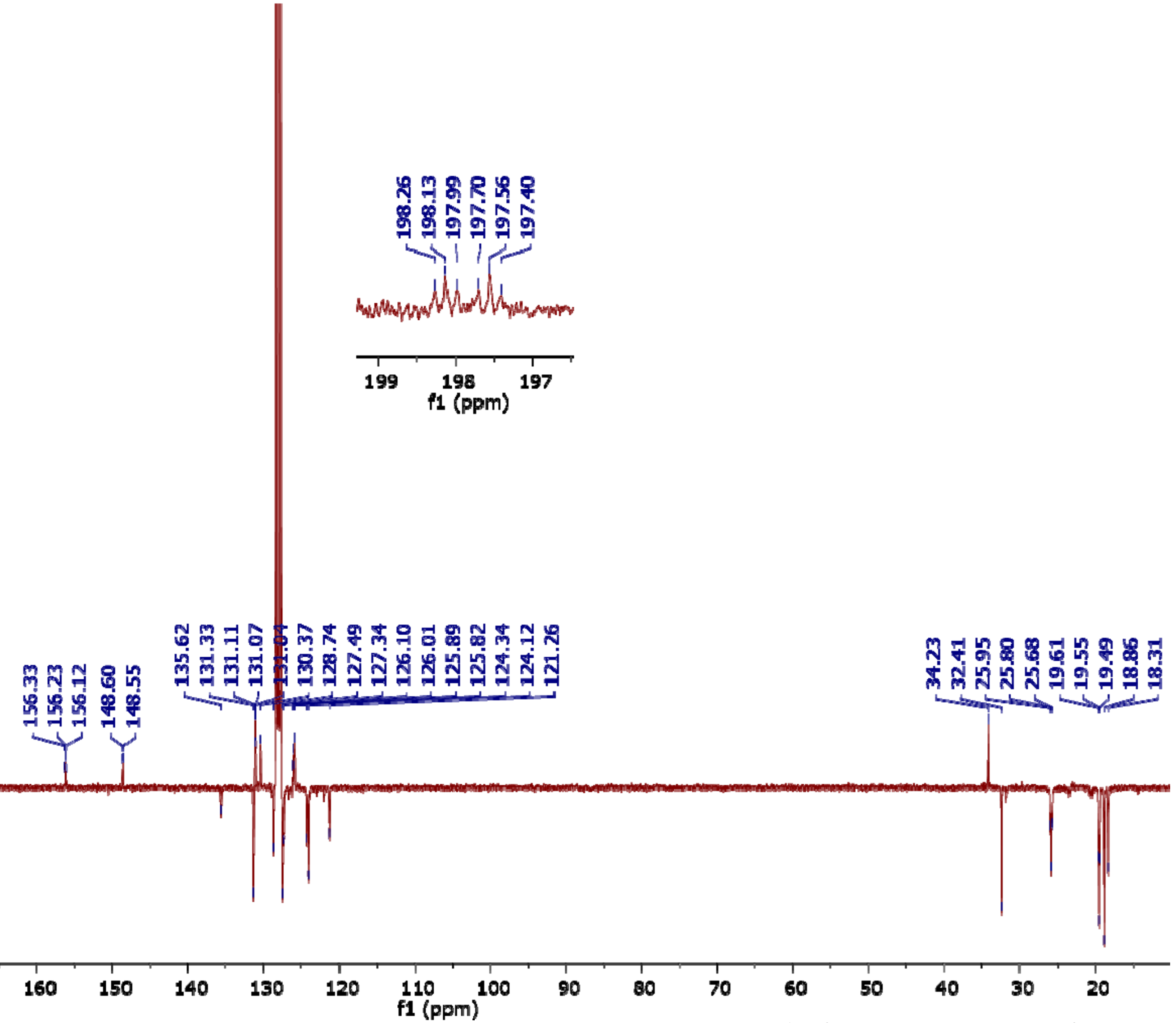

Figure S7. $^{13}C\{^1H\}$-apt NMR spectrum (75.48 MHz, benzene-d_6, 298 K) of Rh(κ^1-C^2-quinolinyl-4-Me){κ^3-P,O,P-[xant(P^{i}Pr$_2$)$_2$]} (**3**).

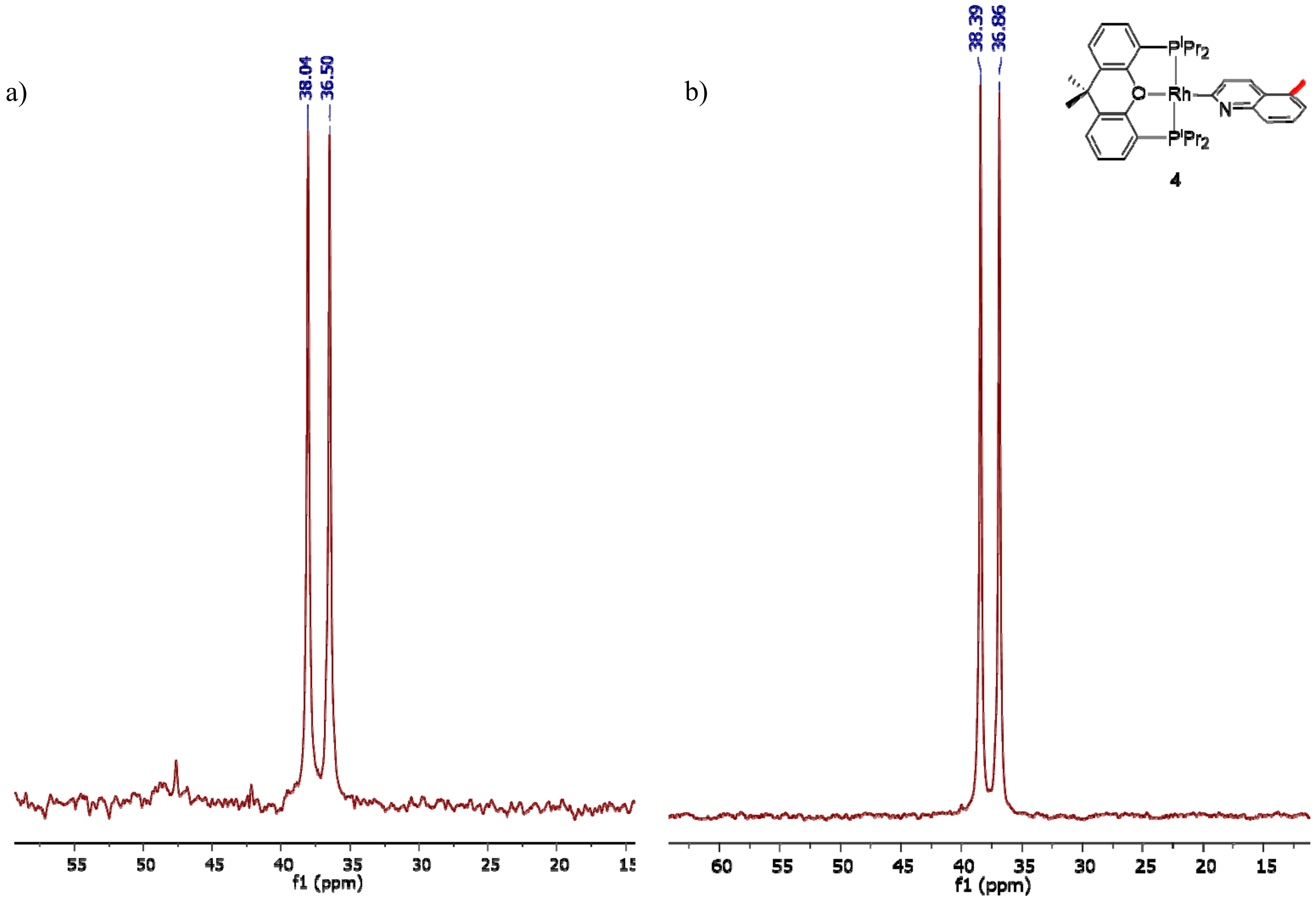

Figure S8. (a) $^{31}P\{^1H\}$ NMR spectrum (121.49 MHz, *n*-octane, 298 K) of the reaction of **1** with 5-methylquinoline after 48 h at 80 ºC. (b) $^{31}P\{^1H\}$ NMR spectrum (121.49 MHz, benzene-d_6, 298 K) of Rh(κ^1-C^2-quinolinyl-5-Me){κ^3-P,O,P-[xant(P^{i}Pr$_2$)$_2$]} (**4**).

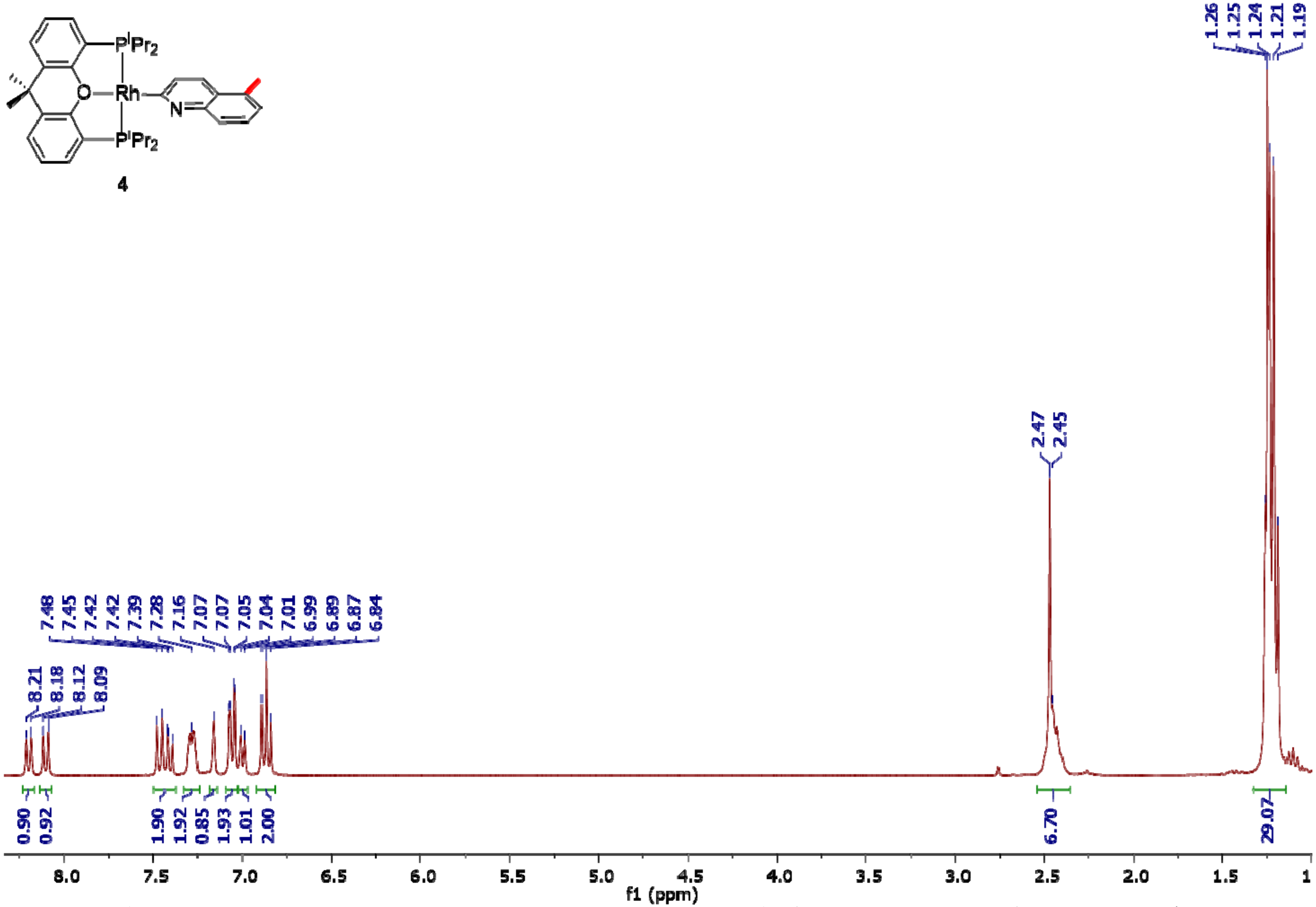

Figure S9. ^{1}H NMR spectrum (300.13 MHz, benzene-d_6, 298 K) of Rh(κ^1-C^2-quinolinyl-5-Me){κ^3-P,O,P-[xant(P^{i}Pr$_2$)$_2$]} (**4**).

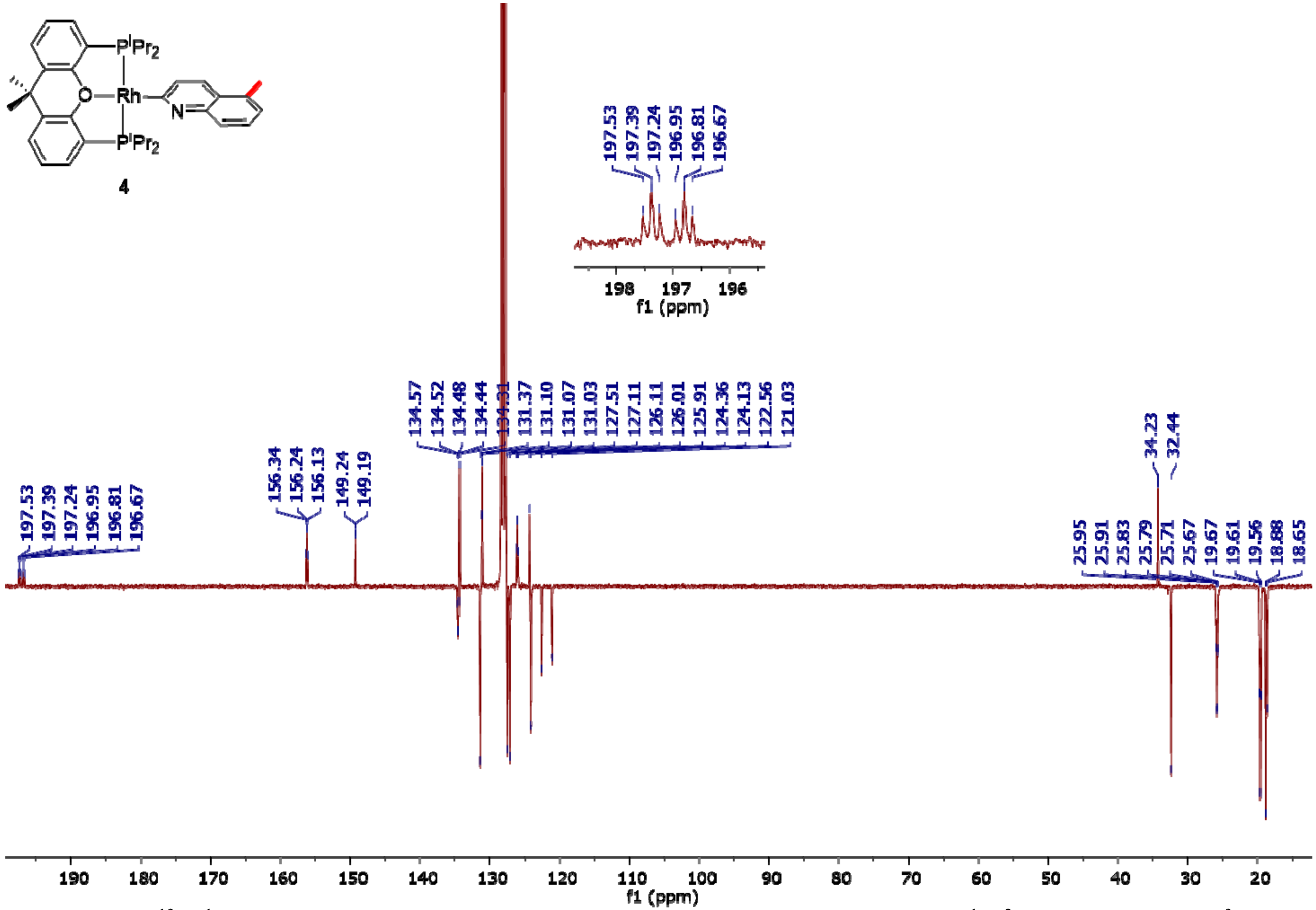

Figure S10. $^{13}C\{^1H\}$-apt NMR spectrum (75.48 MHz, benzene-d_6, 298 K) of Rh(κ^1-C^2-quinolinyl-5-Me){κ^3-P,O,P-[xant(P^iPr_2)$_2$]} (**4**).

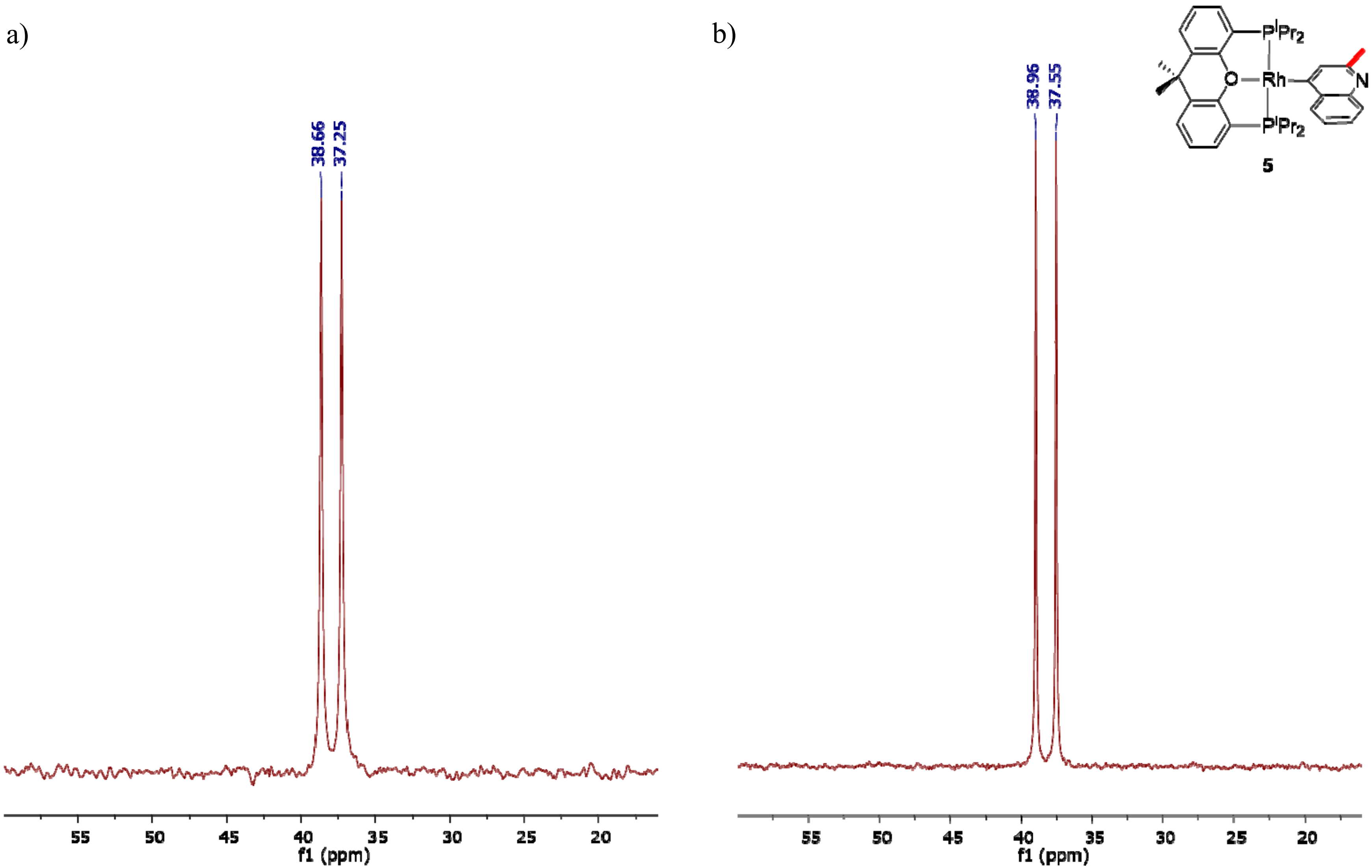

Figure S11. (a) $^{31}P\{^1H\}$ NMR spectrum (121.49 MHz, *n*-octane, 298 K) of the reaction of **1** with 2-methylquinoline after 72 h at 80 ºC. (b) $^{31}P\{^1H\}$ NMR spectrum (121.49 MHz, benzene-d_6, 298 K) of Rh(κ^1-C^4-quinolinyl-2-Me){κ^3-P,O,P-[xant(P^{i}Pr$_2$)$_2$]} (**5**).

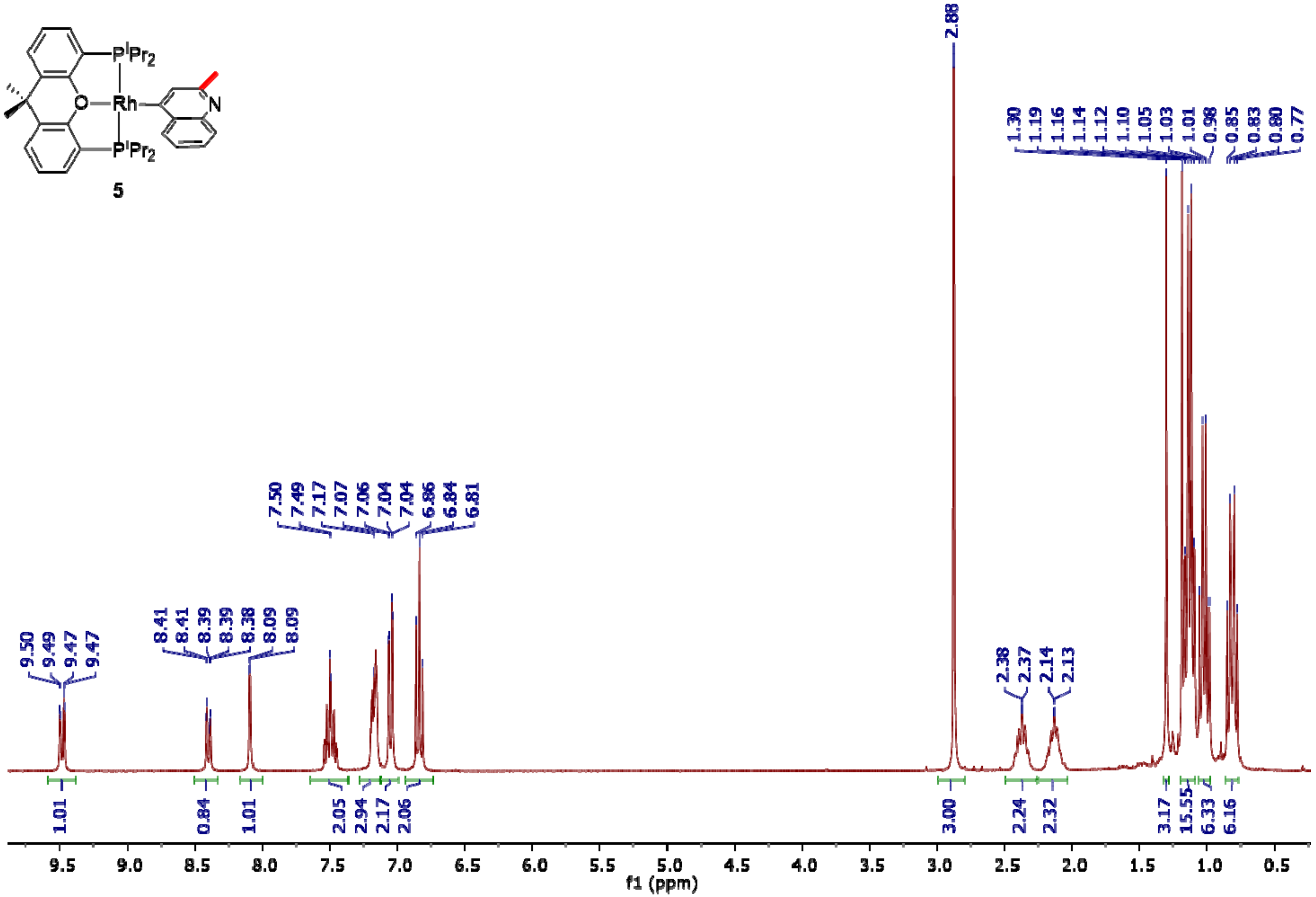

Figure S12. ^{1}H NMR spectrum (300.13 MHz, benzene-d_6, 298 K) of Rh(κ^1-C^4-quinolinyl-2-Me){κ^3-P,O,P-[xant(P^{i}Pr$_2$)$_2$]} (**5**).

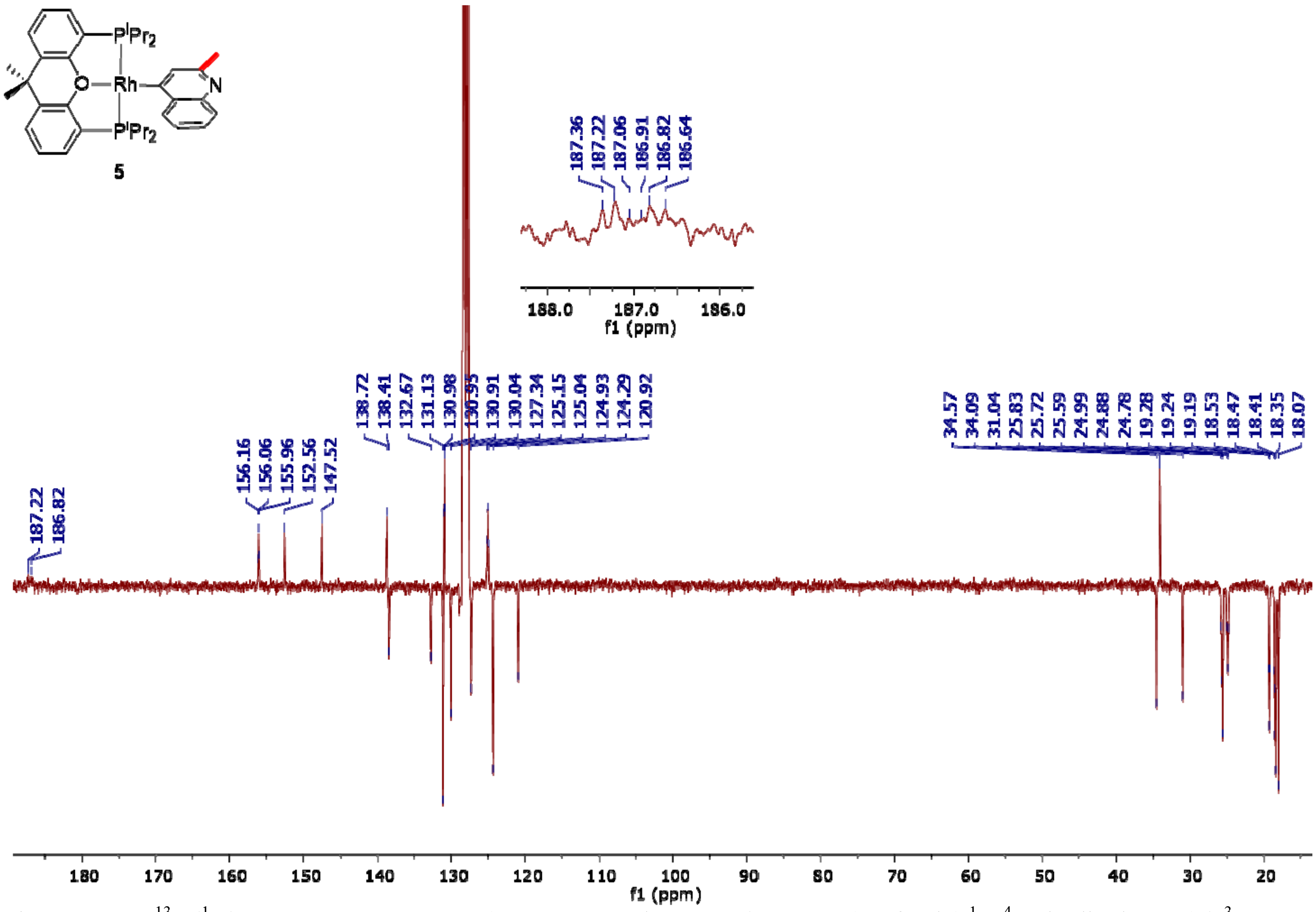

Figure S13. $^{13}C\{^{1}H\}$-apt NMR spectrum (75.48 MHz, benzene-d_6, 298 K) of Rh(κ^1-C^4-quinolinyl-2-Me){κ^3-P,O,P-[xant(P^iPr_2)$_2$]} (**5**).

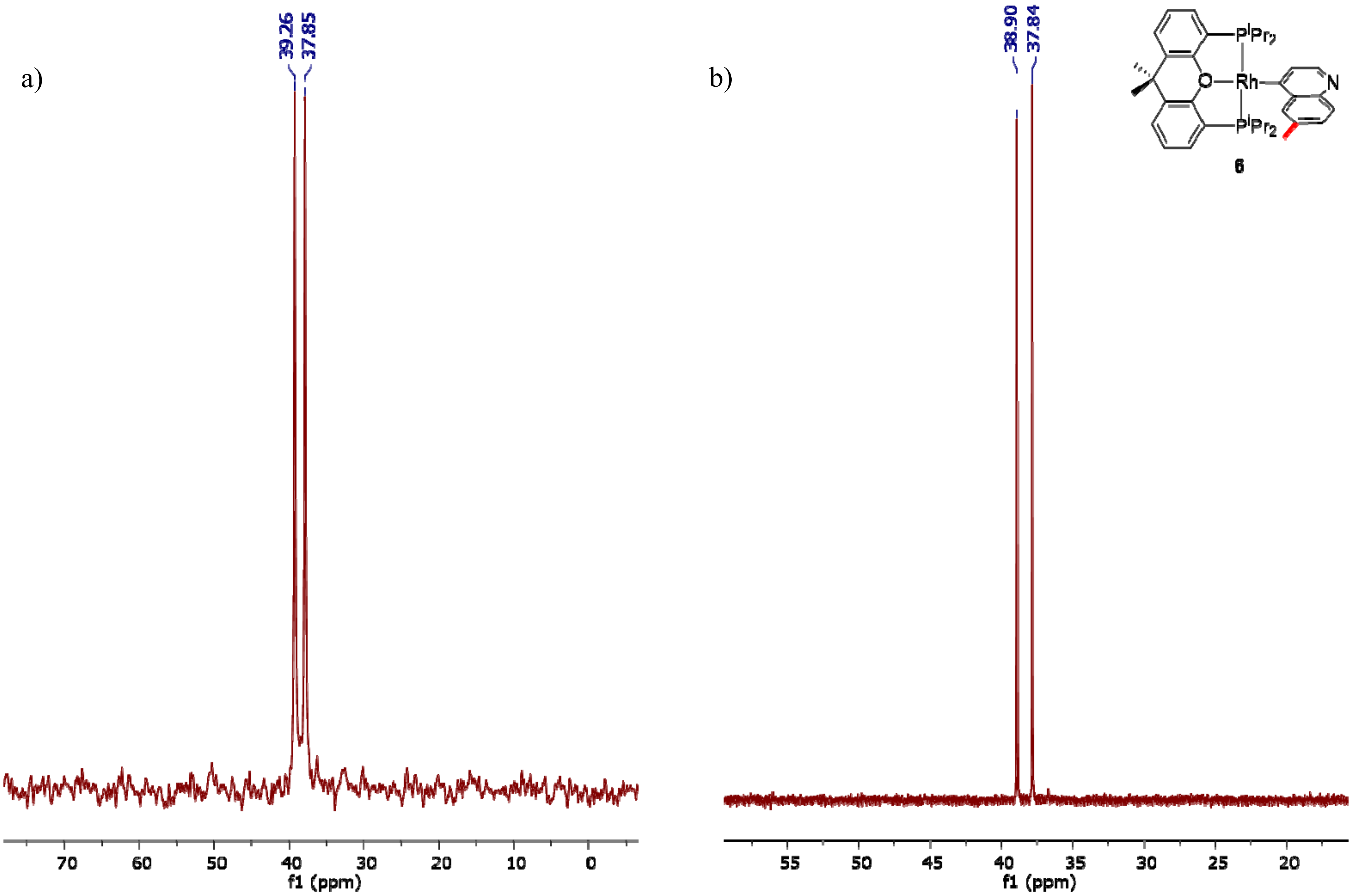

Figure S14. (a) $^{31}P\{^{1}H\}$ NMR spectrum (121.49 MHz, *n*-octane, 298 K) of the reaction of **1** with 6-methylquinoline after 48 h at 80 ºC. (b) $^{31}P\{^{1}H\}$ NMR spectrum (161.98 MHz, benzene-d_6, 298 K) of Rh(κ^1-C^4-quinolinyl-6-Me){κ^3-P,O,P-[xant(P^{i}Pr$_2$)$_2$]} (**6**).

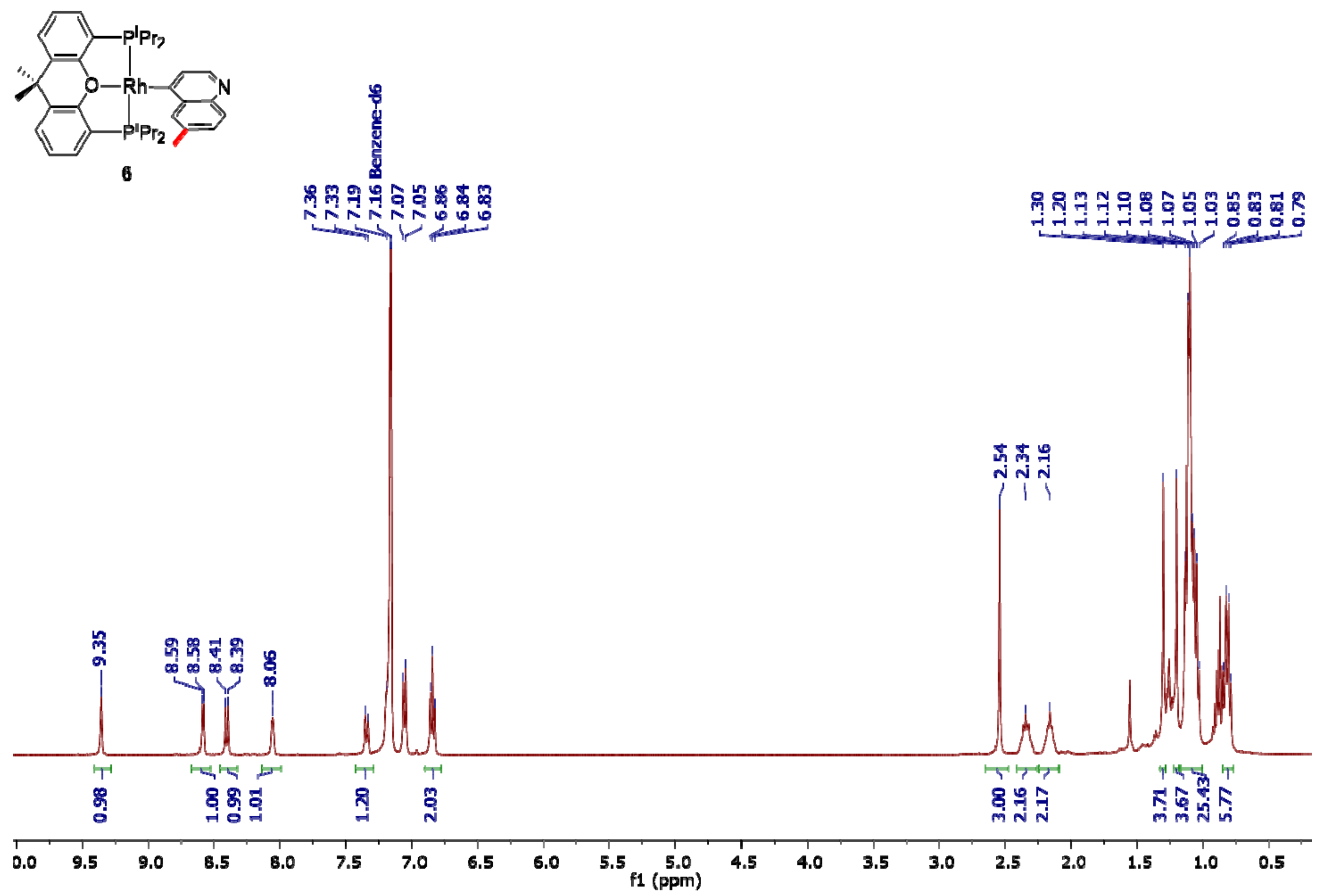

Figure S15. ^{1}H NMR spectrum (300.13 MHz, benzene-d_6, 298 K) of Rh(κ^1-C^4-quinolinyl-6-Me){κ^3-P,O,P-[xant(P^{i}Pr$_2$)$_2$]} (**6**).

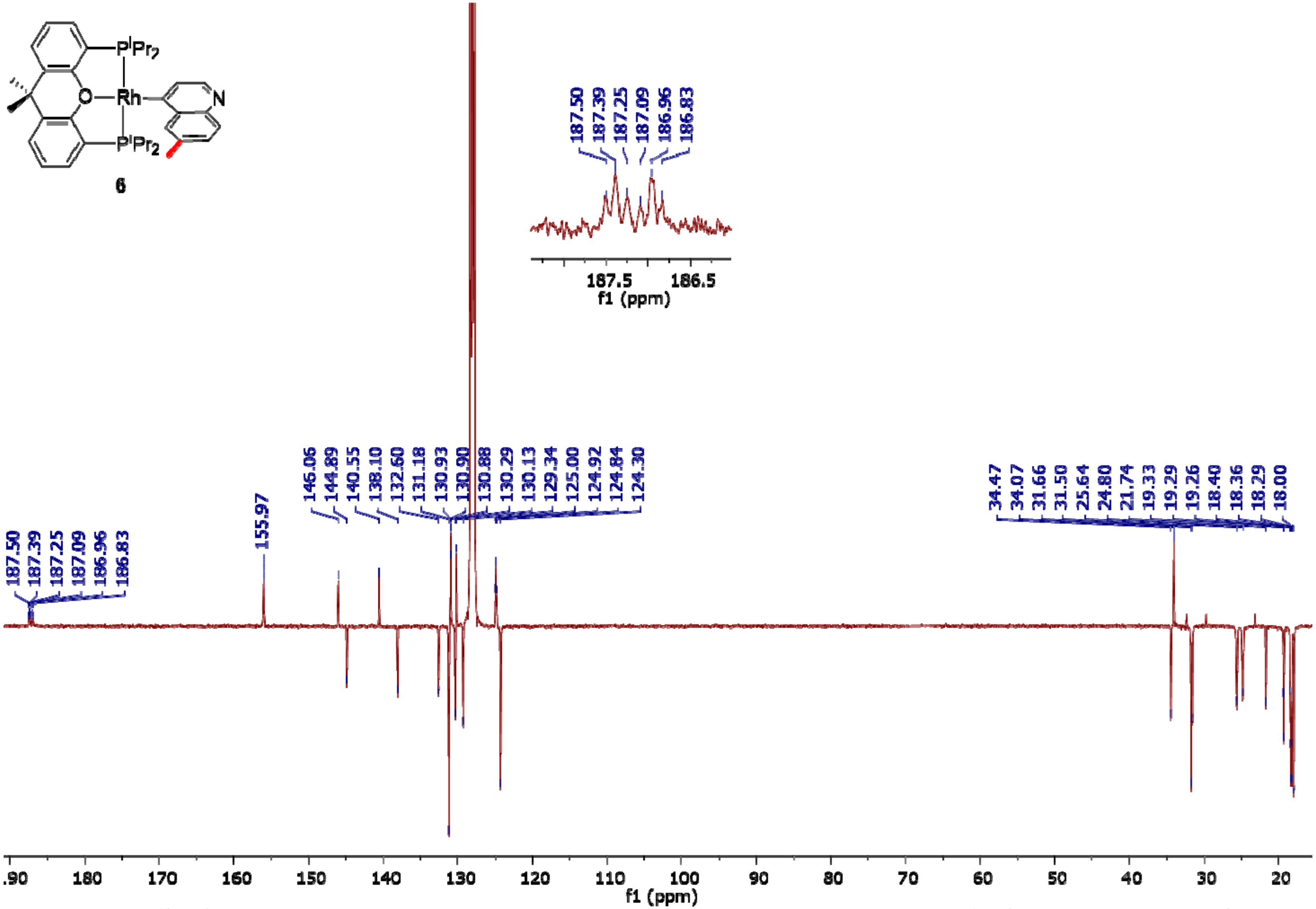

Figure S16. $^{13}C\{^1H\}$-apt NMR spectrum (100.62 MHz, benzene-d_6, 298 K) of Rh(κ^1-C^4-quinolinyl-6-Me){κ^3-P,O,P-[xant(P^{i}Pr$_2$)$_2$]} (**6**).

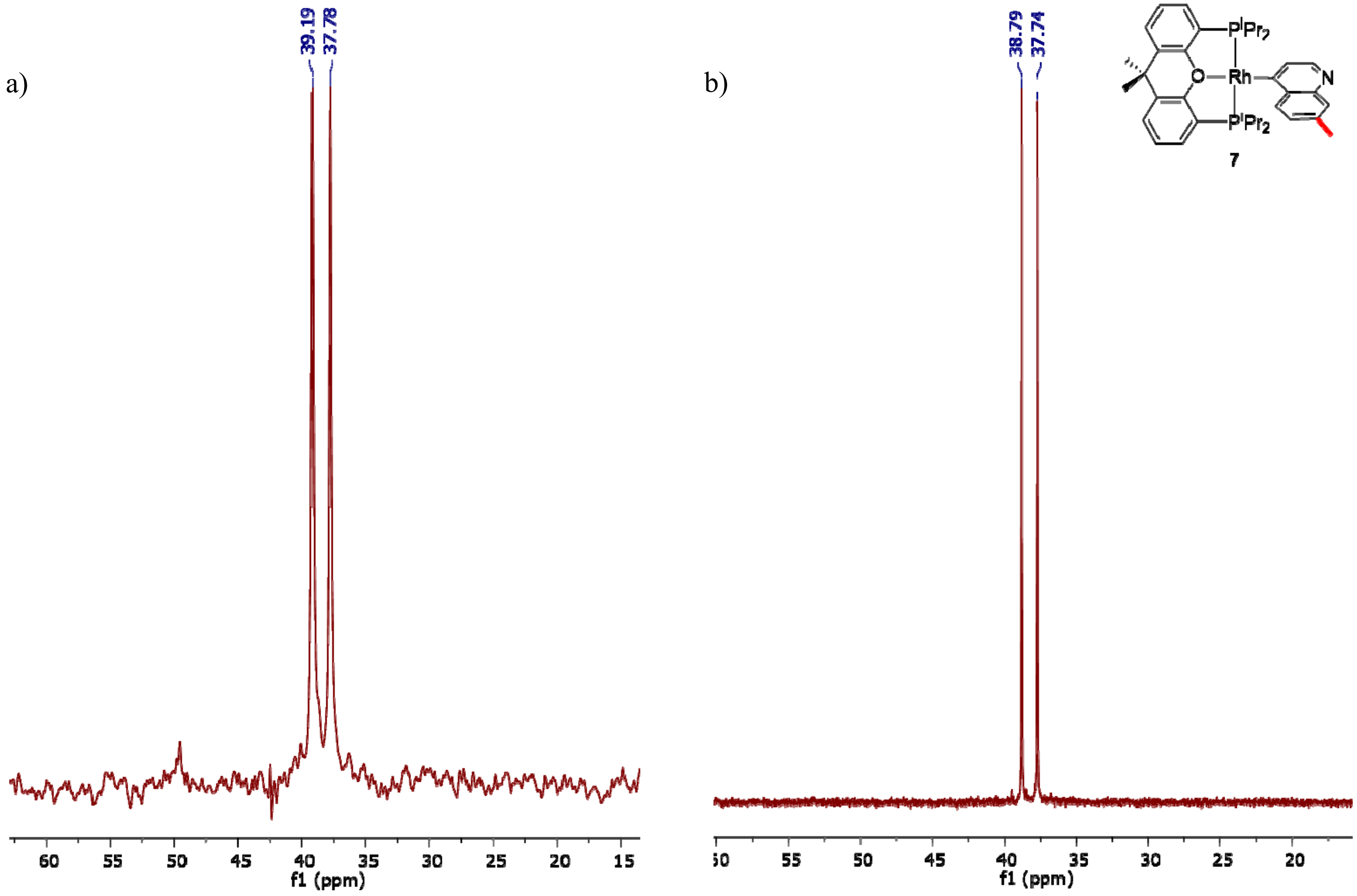

Figure S17. (a) $^{31}P\{^{1}H\}$ NMR spectrum (121.49 MHz, *n*-octane, 298 K) of the reaction of **1** with 7-methylquinoline after 48 h at 80 ºC. (b) $^{31}P\{^{1}H\}$ NMR spectrum (161.98 MHz, benzene-d_6, 298 K) of Rh(κ^1-C^4-quinolinyl-7-Me)$\{\kappa^3$-P,O,P-[xant(P^{i}Pr$_2$)$_2$]$\}$ (**7**).

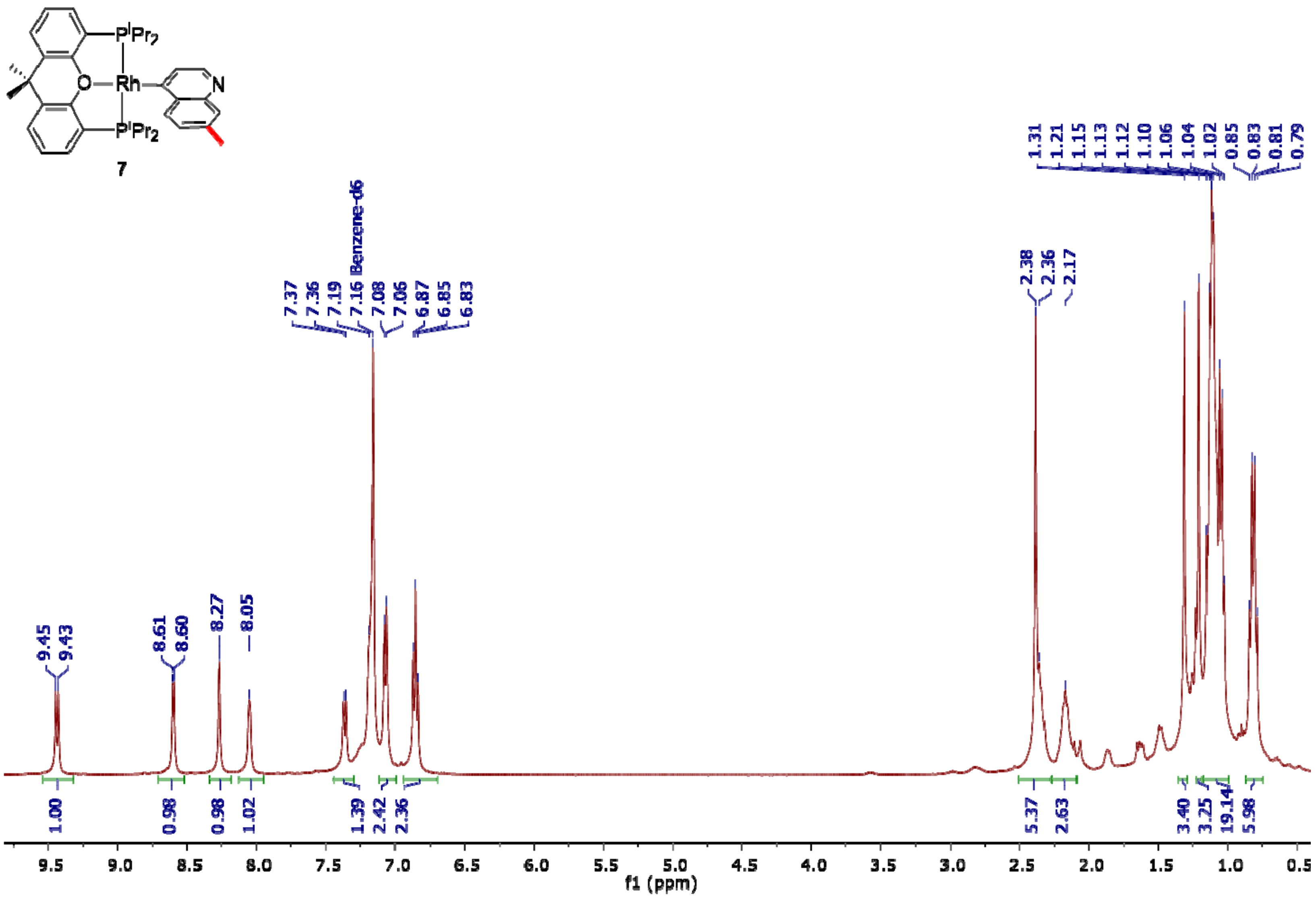

Figure S18. ^{1}H NMR spectrum (400.13 MHz, benzene-d_6, 298 K) of Rh(κ^1-C^4-quinolinyl-7-Me){κ^3-P,O,P-[xant(P^{i}Pr$_2$)$_2$]} (**7**).

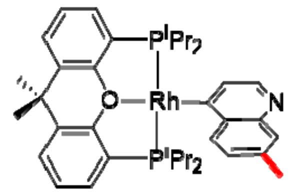

7

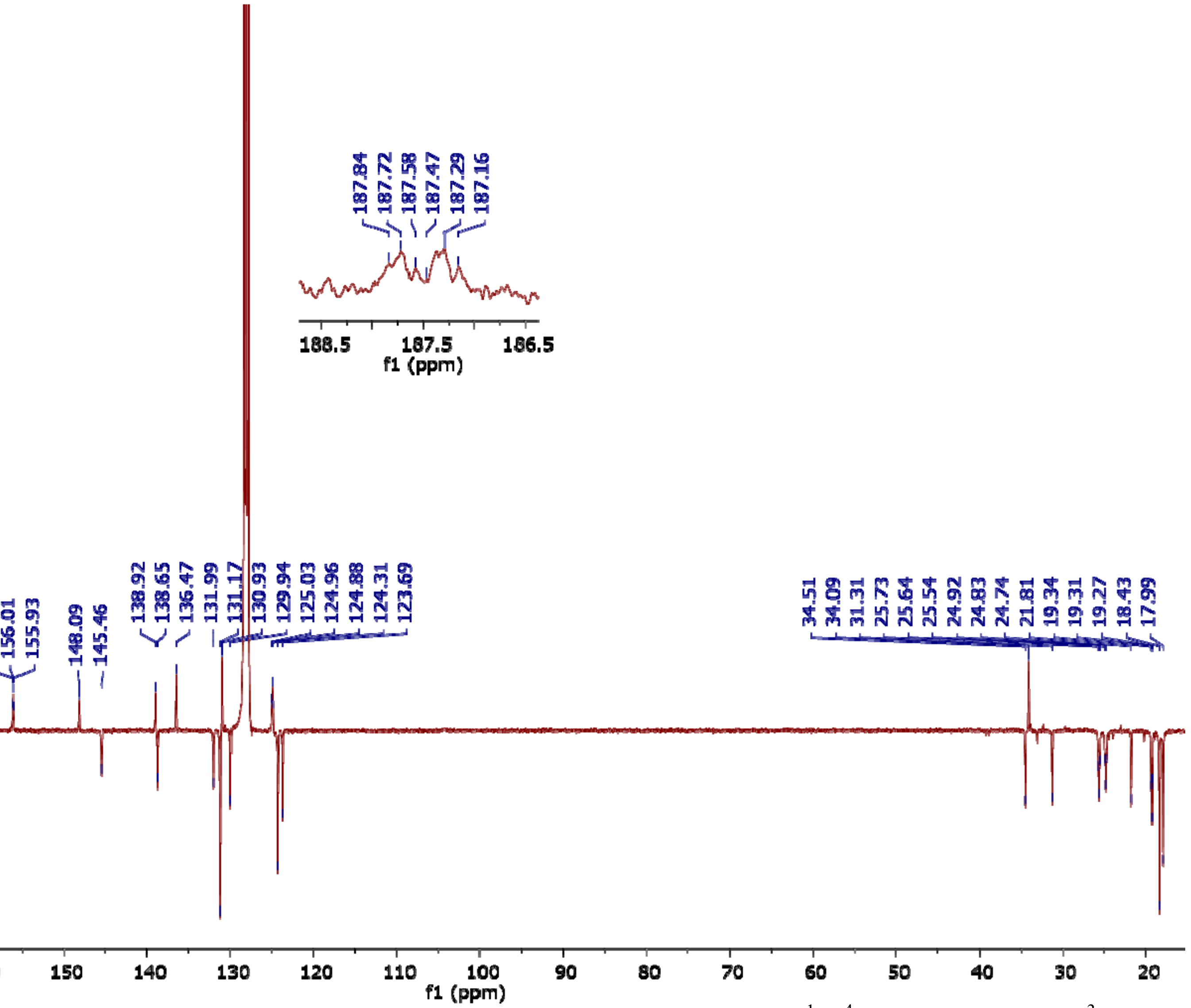

Figure S19. $^{13}C\{^{1}H\}$-apt NMR spectrum (100.62 MHz, benzene-d_6, 298 K) of Rh(κ^1-C^4-quinolinyl-7-Me){κ^3-P,O,P-[xant(P^{i}Pr$_2$)$_2$]} (**7**).

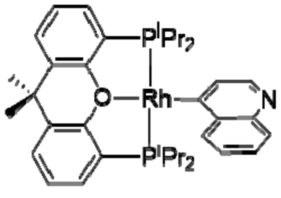

8

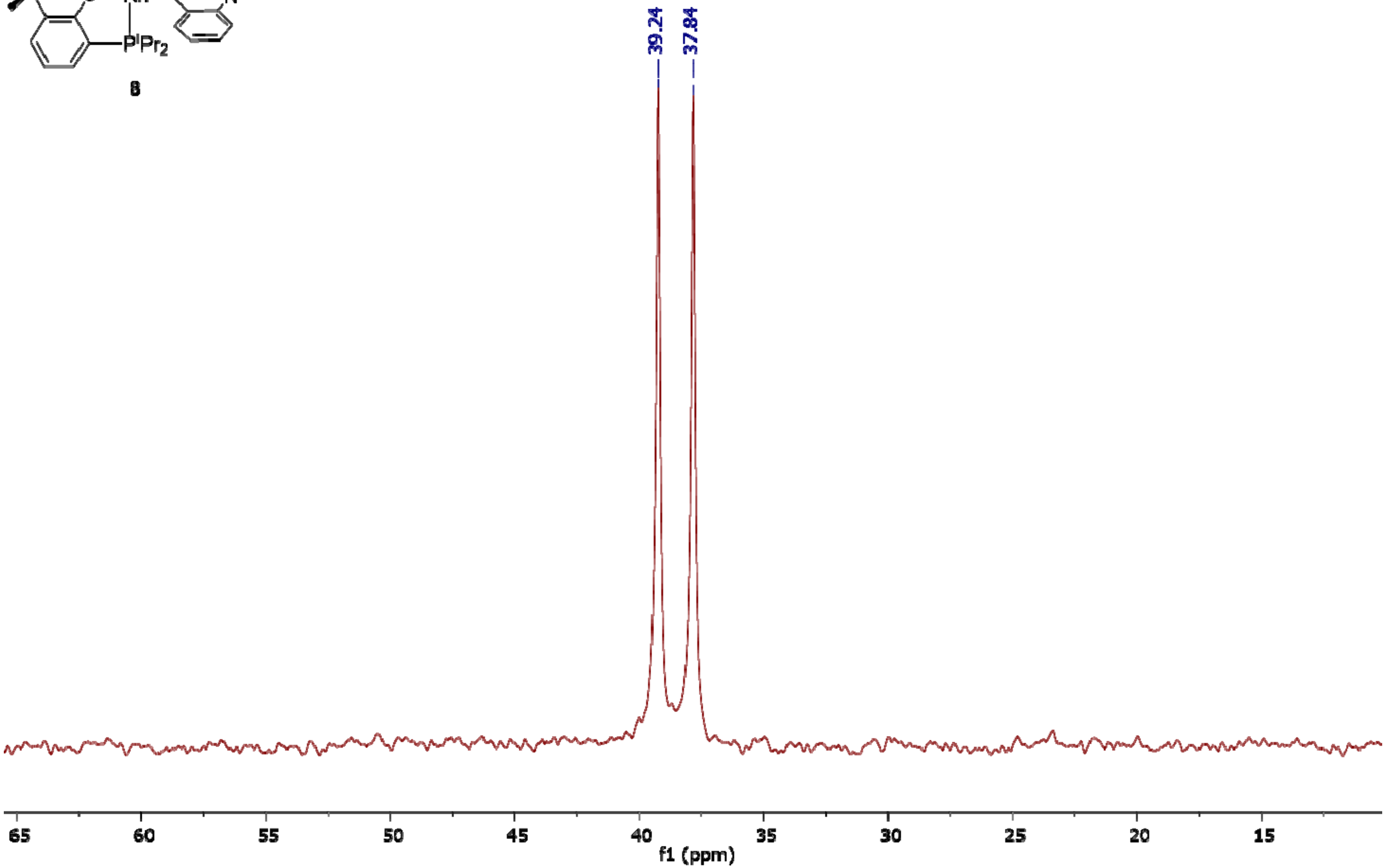

Figure S20. $^{31}P\{^{1}H\}$ NMR spectrum (121.49 MHz, benzene-d_6, 298 K) of Rh(κ^{1}-C^{4}-quinolinyl){κ^{3}-P,O,P-[xant(P^{i}Pr$_2$)$_2$]} (**8**).

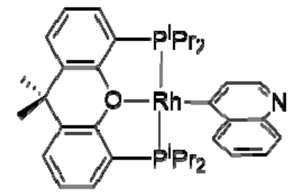

8

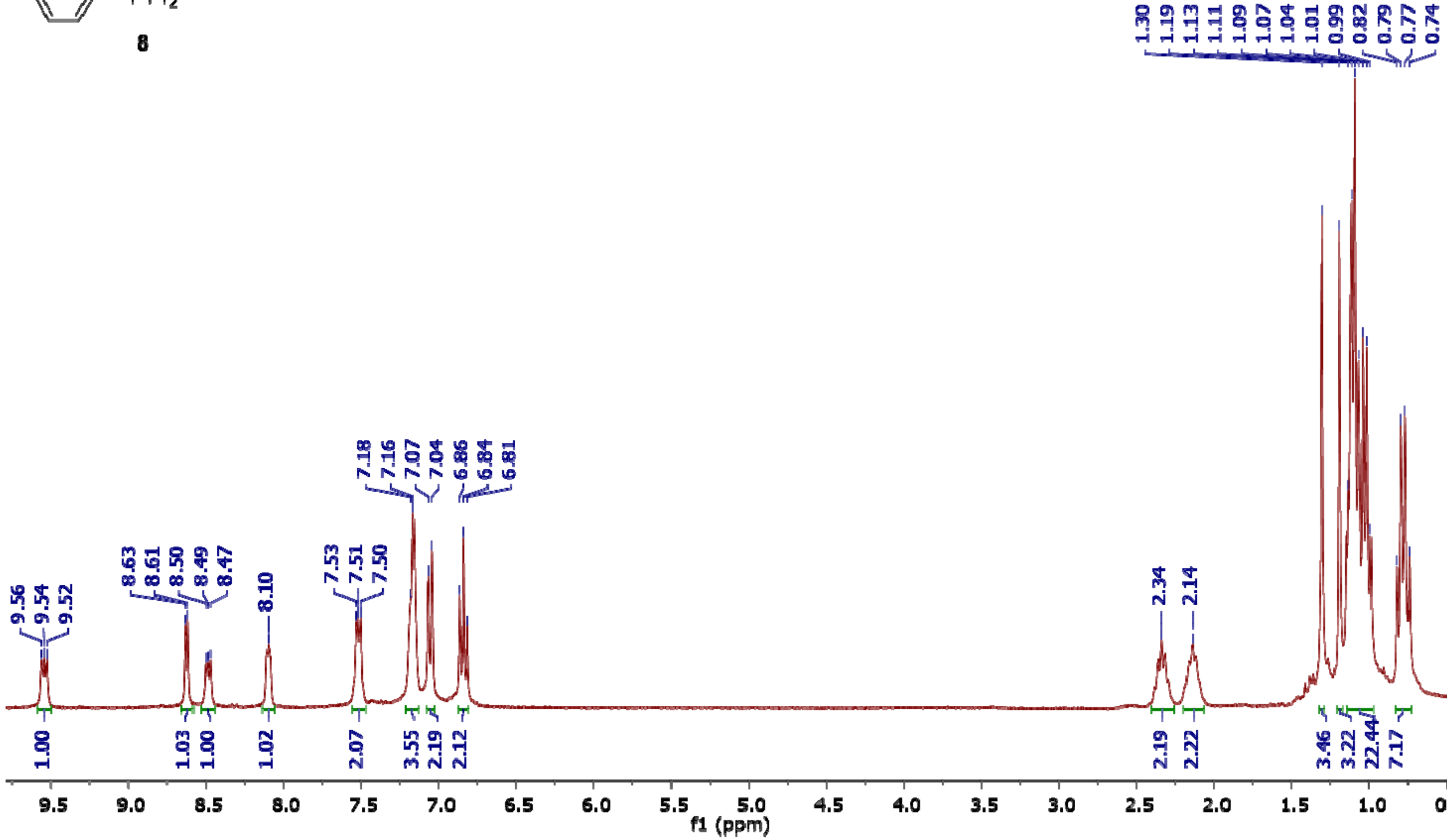

Figure S21. ^{1}H NMR spectrum (300.13 MHz, benzene-d_6, 298 K) of Rh(κ^1-C^4-quinolinyl){κ^3-P,O,P-[xant(P^{i}Pr$_2$)$_2$]} (**8**).

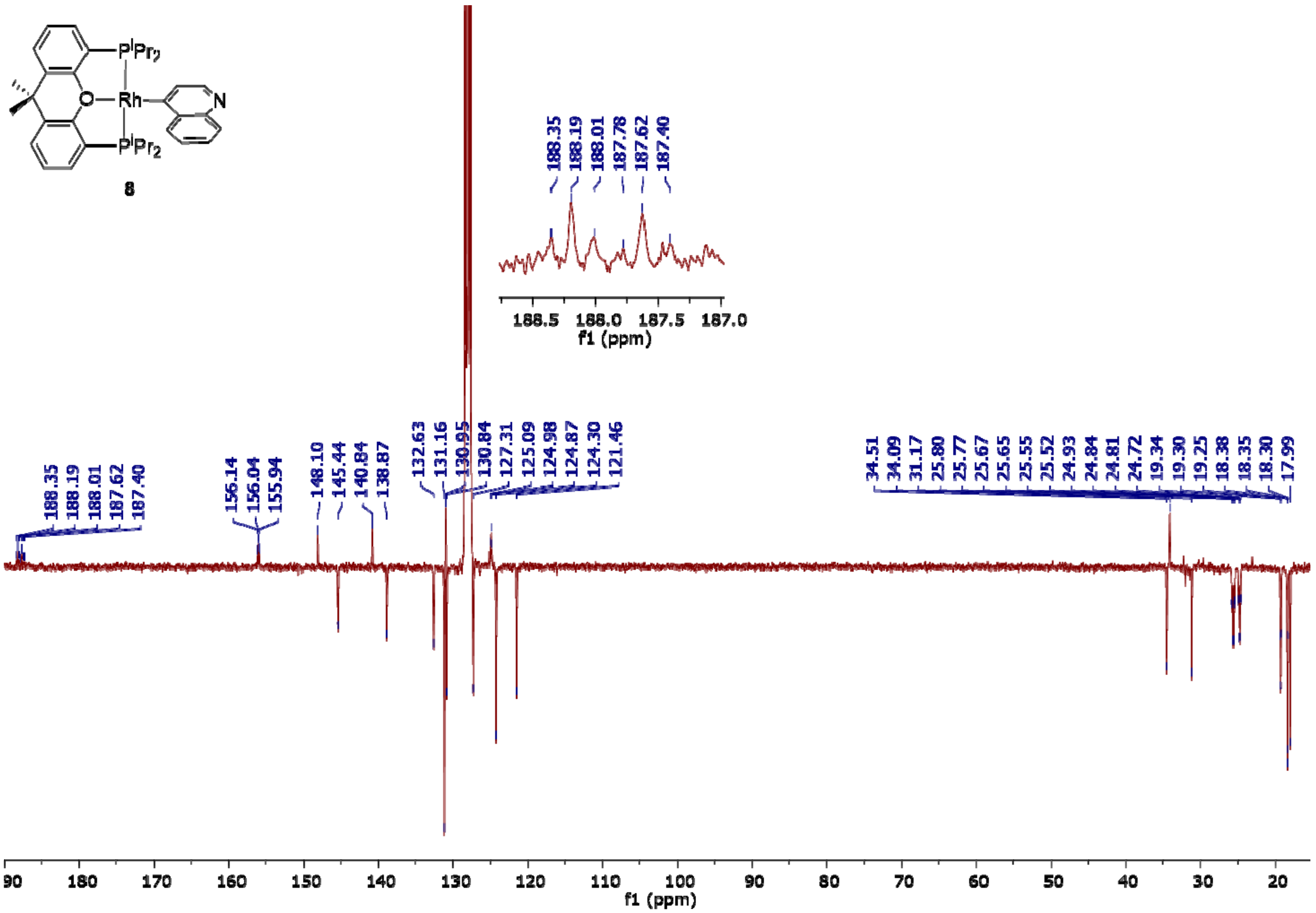

Figure S22. $^{13}C\{^{1}H\}$-apt NMR spectrum (75.48 MHz, benzene-d_6, 298 K) of Rh(κ^1-C^4-quinolinyl){κ^3-P,O,P-[xant(P^{i}Pr$_2$)$_2$]} (**8**).

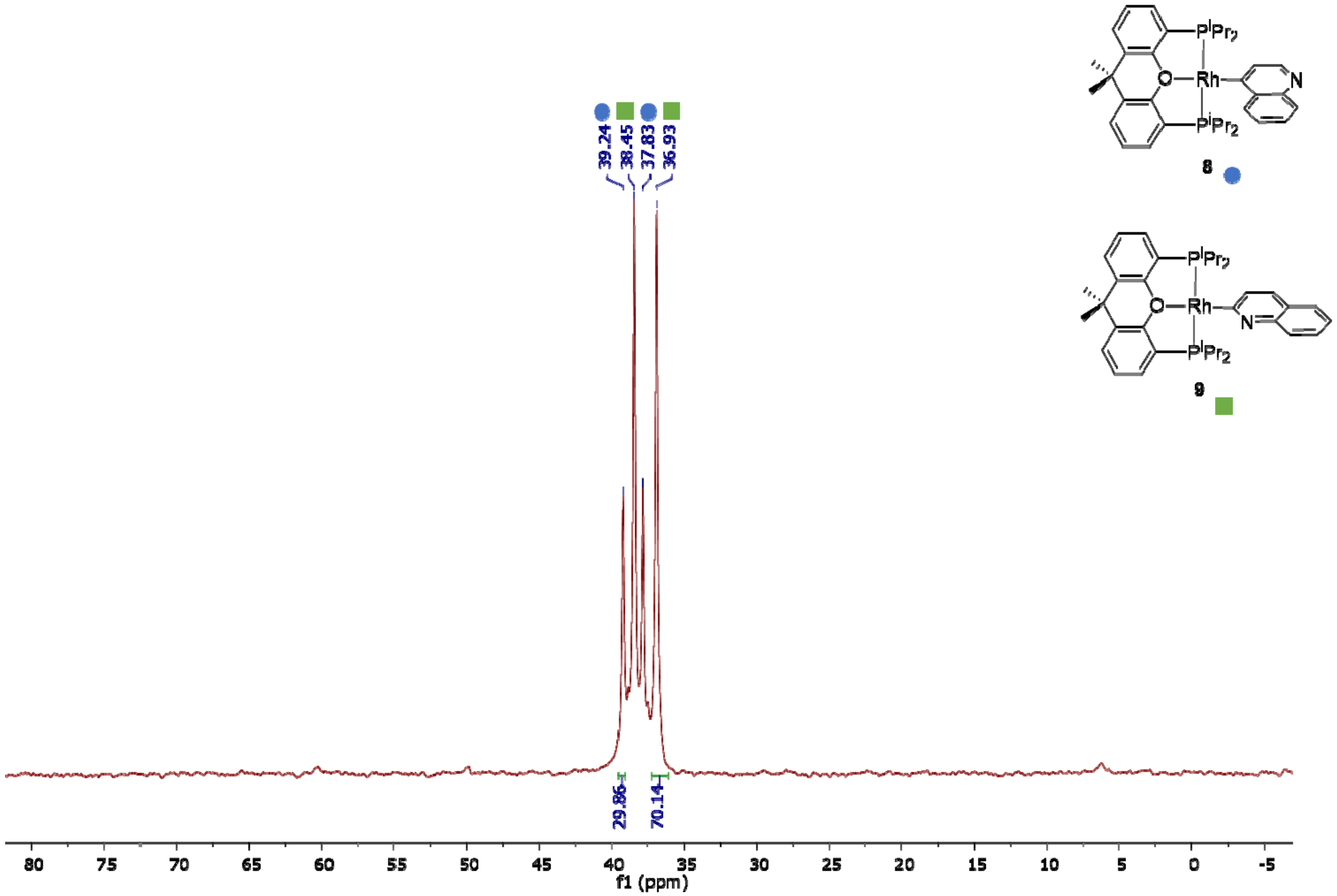

Figure S23. (a) $^{31}P\{^{1}H\}$ NMR spectrum (161.98 MHz, benzene-d_6, 298 K) of the mother liquors of the reaction of **1** with quinoline after 48 h at 80 ºC.

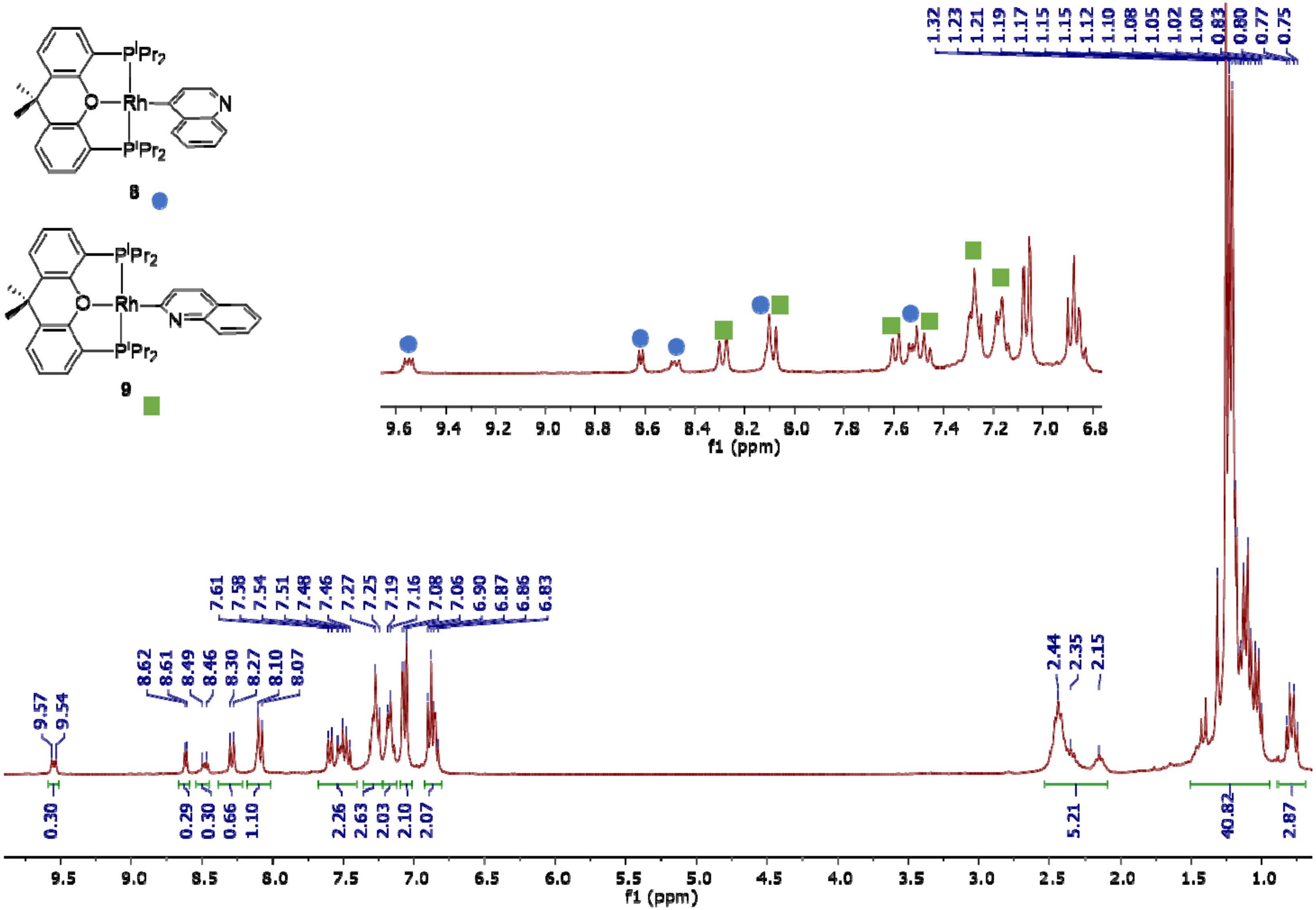

Figure S24. ^{1}H NMR spectrum (300.13 MHz, benzene-d_6, 298 K) of a mixture of complexes Rh(κ^1-C[4]-quinolinyl){κ^3-P,O,P-[xant(P^{i}Pr$_2$)$_2$]} (**8**) and Rh(κ^1-C[2]-quinolinyl){κ^3-P,O,P-[xant(P^{i}Pr$_2$)$_2$]} (**9**).

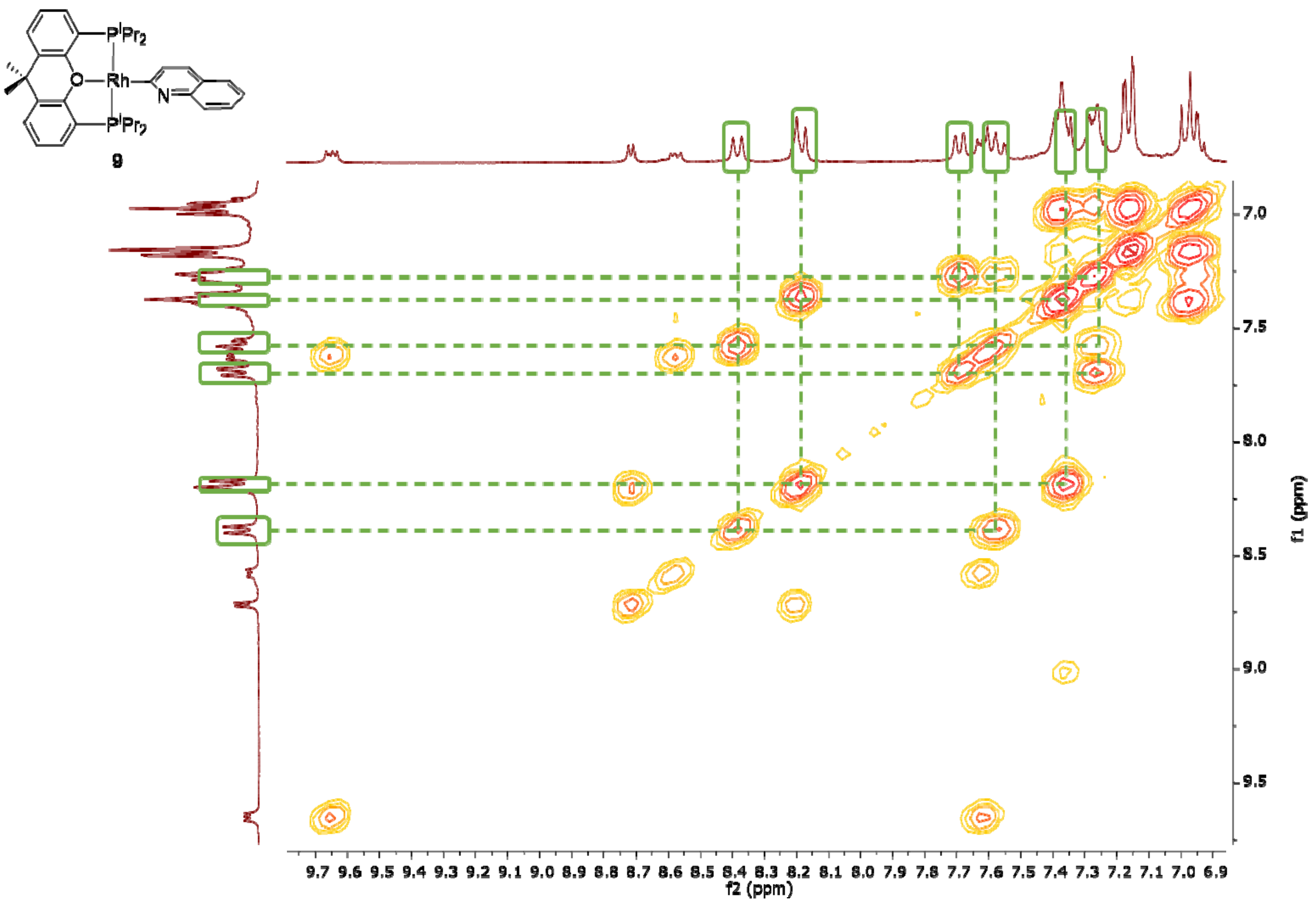

Figure S25. (^{1}H-^{1}H)-COSY NMR spectrum (300.13 MHz, benzene-d_6, 298 K) of a mixture of complexes **8** and **9**. Peaks corresponding to Rh(κ^1-C^2-quinolinyl){κ^3-P,O,P-[xant(P^{i}Pr$_2$)$_2$]} (**9**) are highlighted in green.

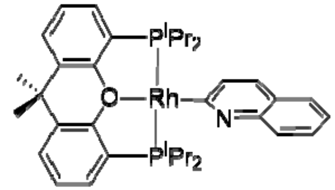

9

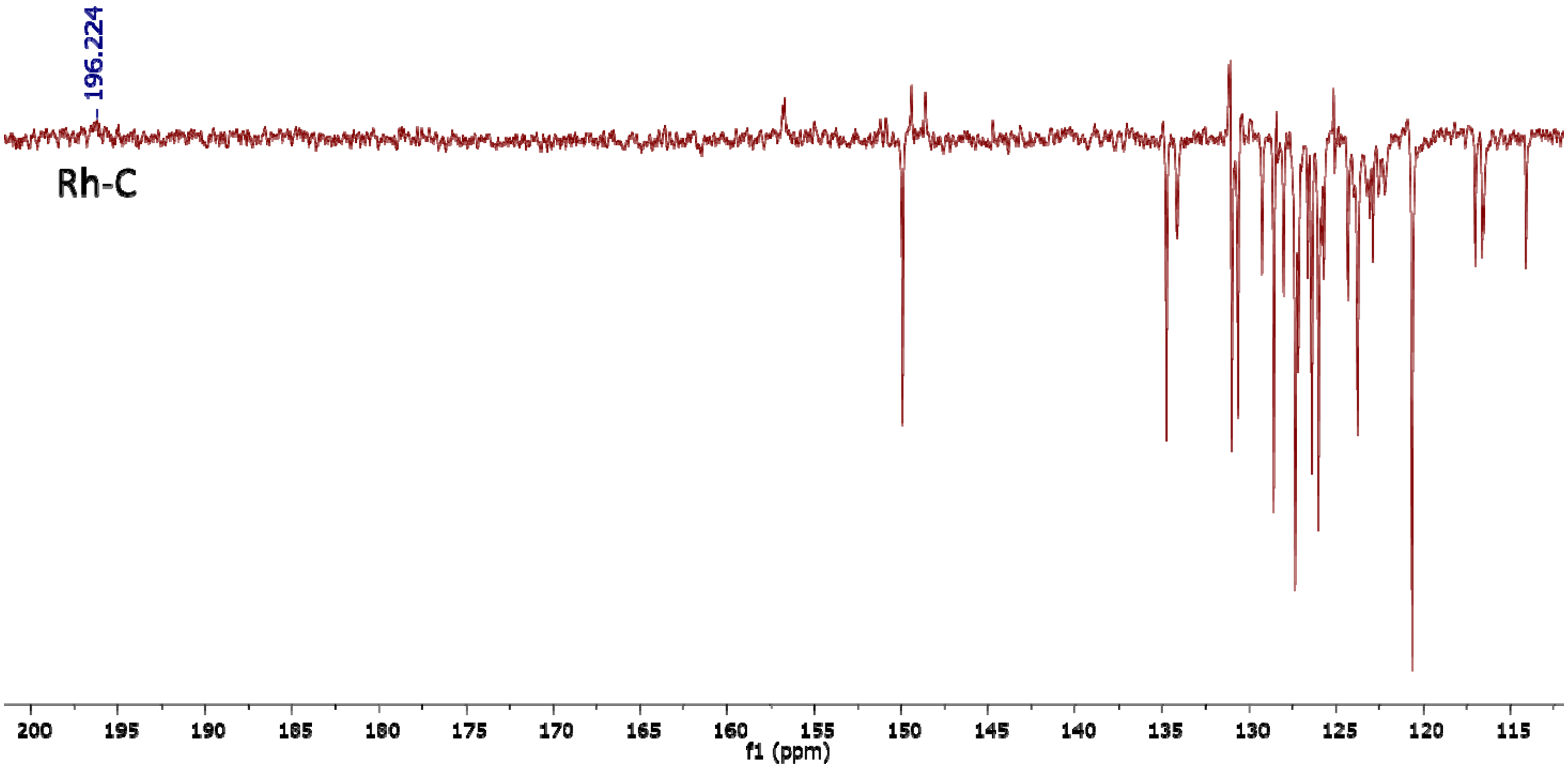

Figure S26. Aromatic region of the $^{13}C\{^1H\}$-apt NMR spectrum (75.48 MHz, *n*-octane, 298 K) of a mixture of complexes Rh(κ^1-C^4-quinolinyl){κ^3-P,O,P-[xant(P^{i}Pr$_2$)$_2$]} (**8**) and Rh(κ^1-C^2-quinolinyl){κ^3-P,O,P-[xant(P^{i}Pr$_2$)$_2$]} (**9**).

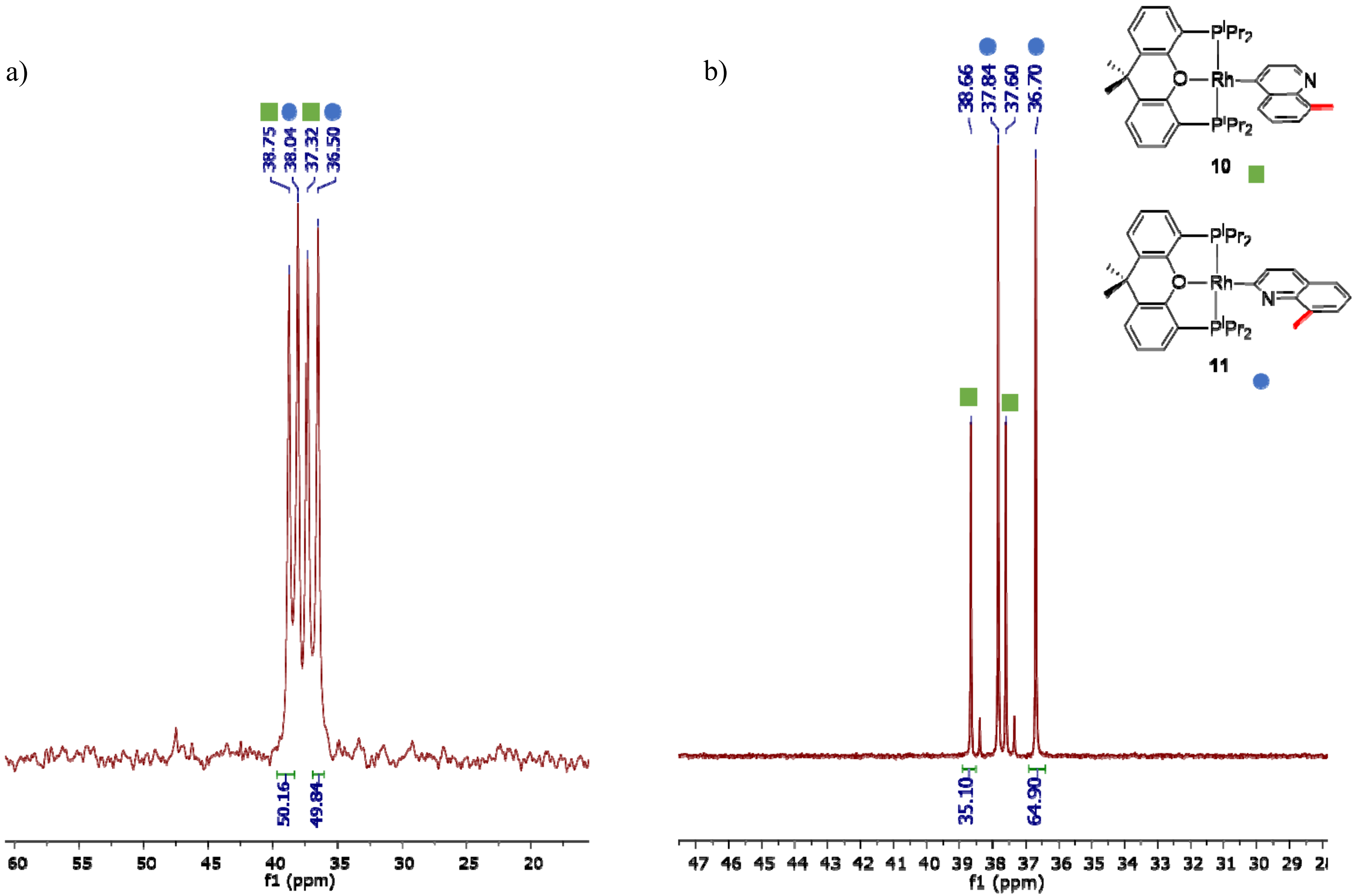

Figure S27. (a) $^{31}P\{^{1}H\}$ NMR spectrum (121.49 MHz, *n*-octane, 298 K) of the reaction of **1** with 8-methylquinoline after 48 h at 80 ºC. (b) $^{31}P\{^{1}H\}$ NMR spectrum (161.98 MHz, benzene-d_6, 298 K) of a mixture of complexes Rh(κ^1-C^4-quinolinyl-8-Me){κ^3-P,O,P-[xant($P^iPr_2)_2$]} (**10**) and Rh(κ^1-C^2-quinolinyl-8-Me){κ^3-P,O,P-[xant($P^iPr_2)_2$]} (**11**).

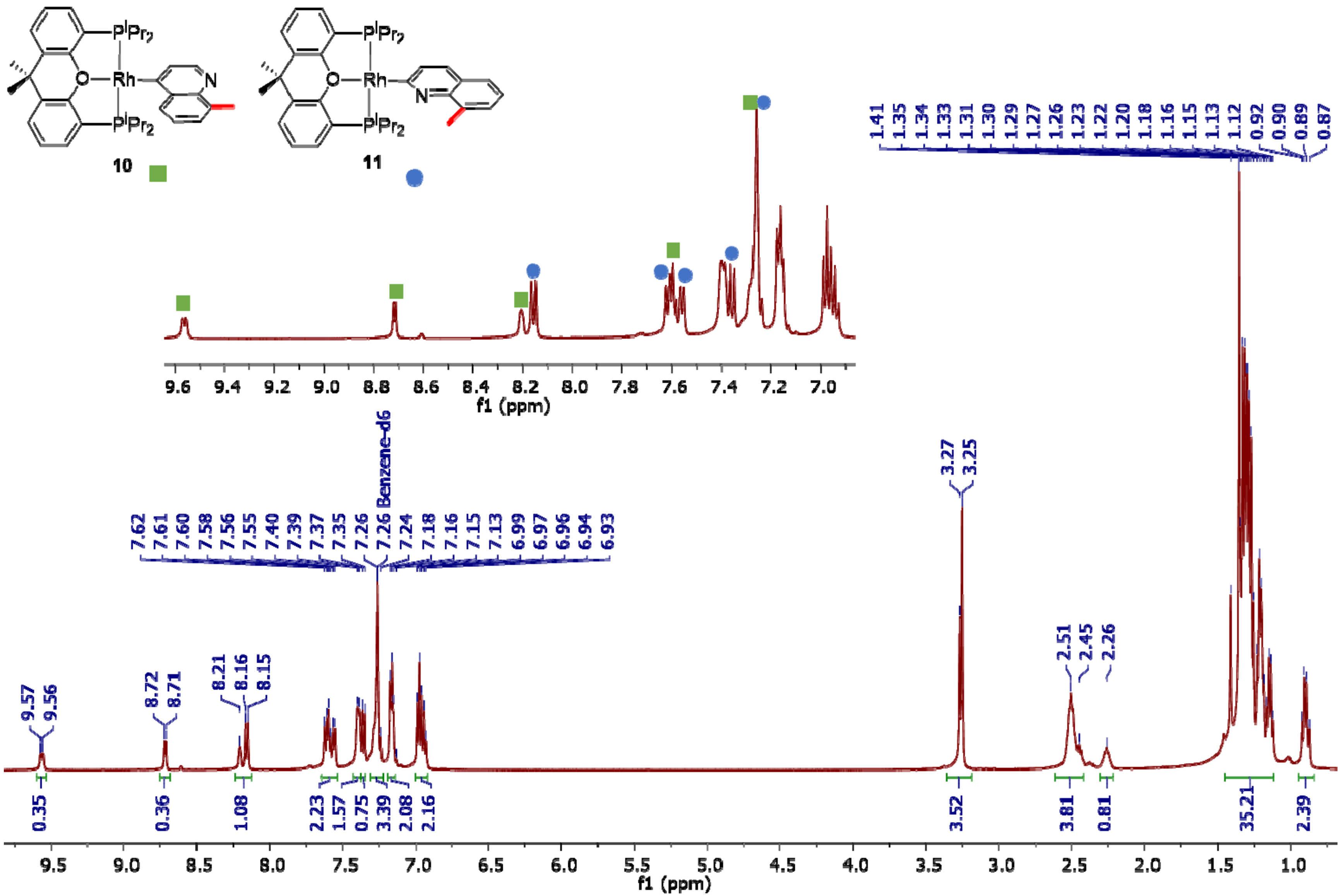

Figure S28. ^{1}H NMR spectrum (500.13 MHz, benzene-d_6, 298 K) of a mixture of complexes Rh(κ^1-C^4-quinolinyl-8-Me){κ^3-P,O,P-[xant(P^{i}Pr$_2$)$_2$]} (**10**) and Rh(κ^1-C^2-quinolinyl-8-Me){κ^3-P,O,P-[xant(P^{i}Pr$_2$)$_2$]} (**11**).

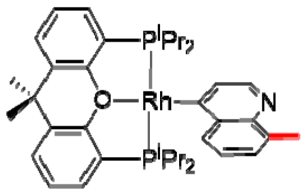

10

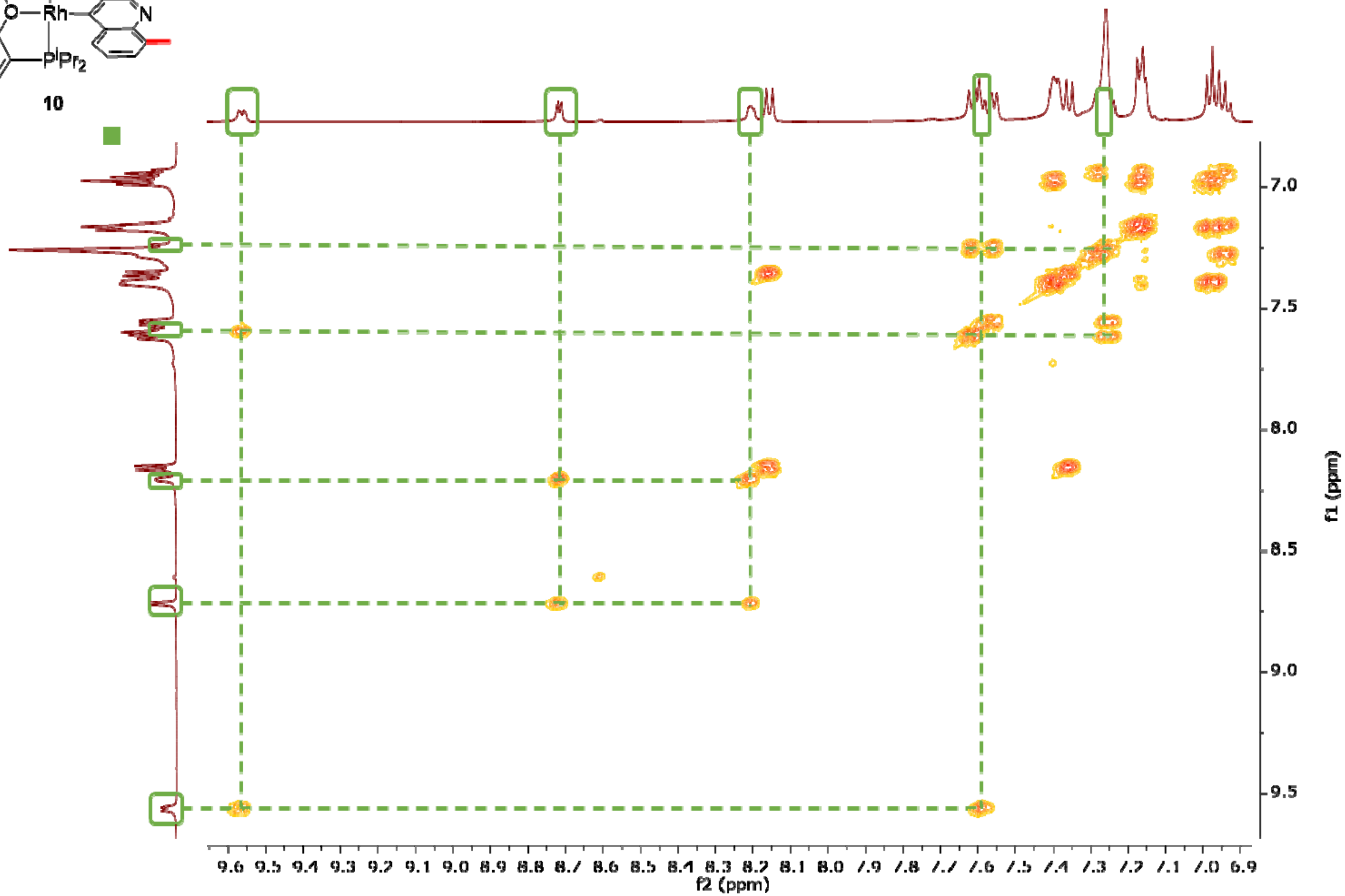

Figure S29. (^{1}H-^{1}H)-COSY NMR spectrum (500.13 MHz, benzene-d_6, 298 K) of a mixture of complexes **10** and **11**. Peaks corresponding to Rh(κ^1-C^4-quinolinyl-8-Me){κ^3-P,O,P-[xant(P^{i}Pr$_2$)$_2$]} (**10**) are highlighted in green.

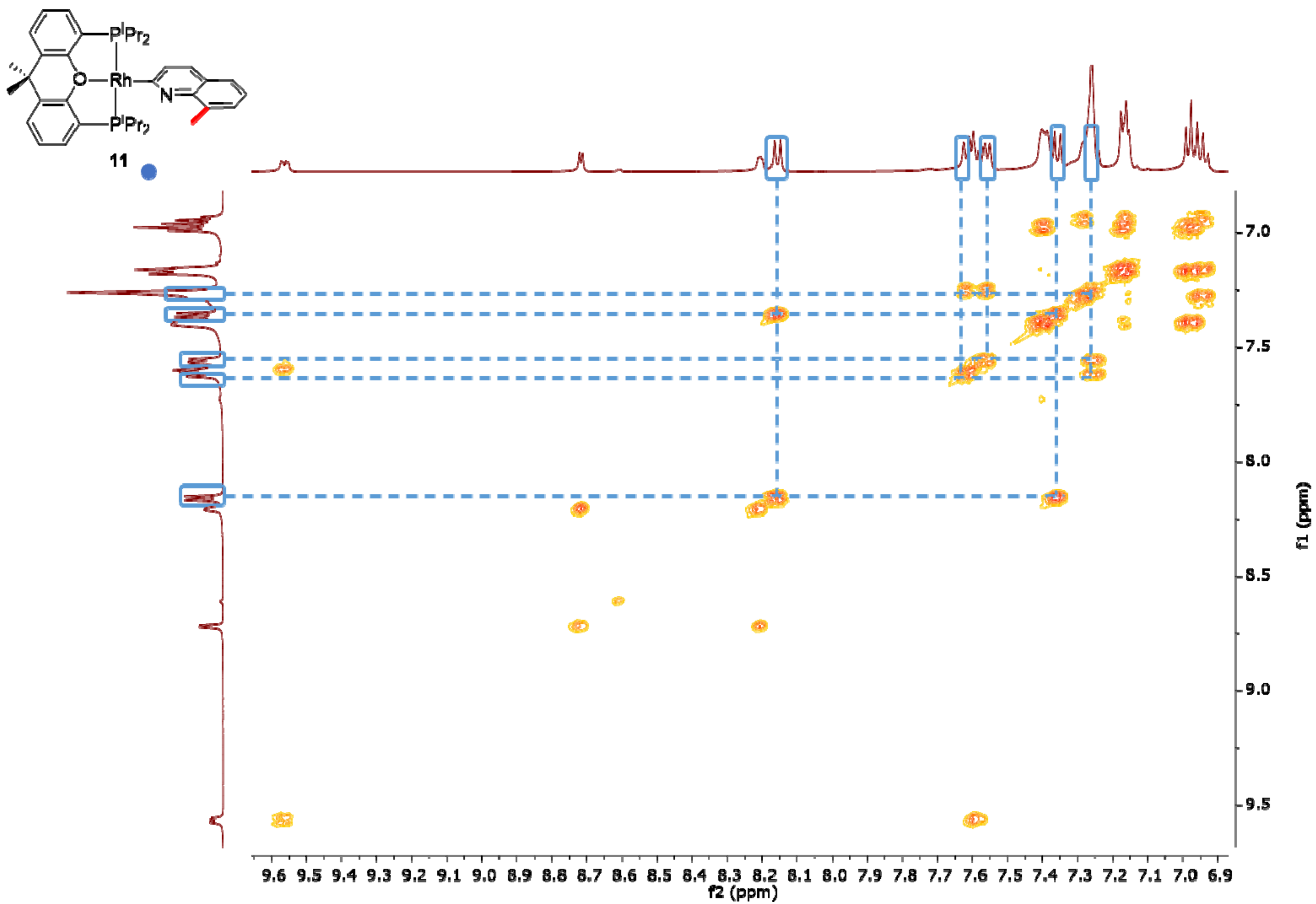

Figure S30. (^{1}H-^{1}H)-COSY NMR spectrum (500.13 MHz, benzene-d_6, 298 K) of a mixture of complexes **8** and **9**. Peaks corresponding to Rh(κ^1-C^2-quinolinyl-8-Me){κ^3-P,O,P-[xant(P^{i}Pr$_2$)$_2$]} (**11**) are highlighted in blue.

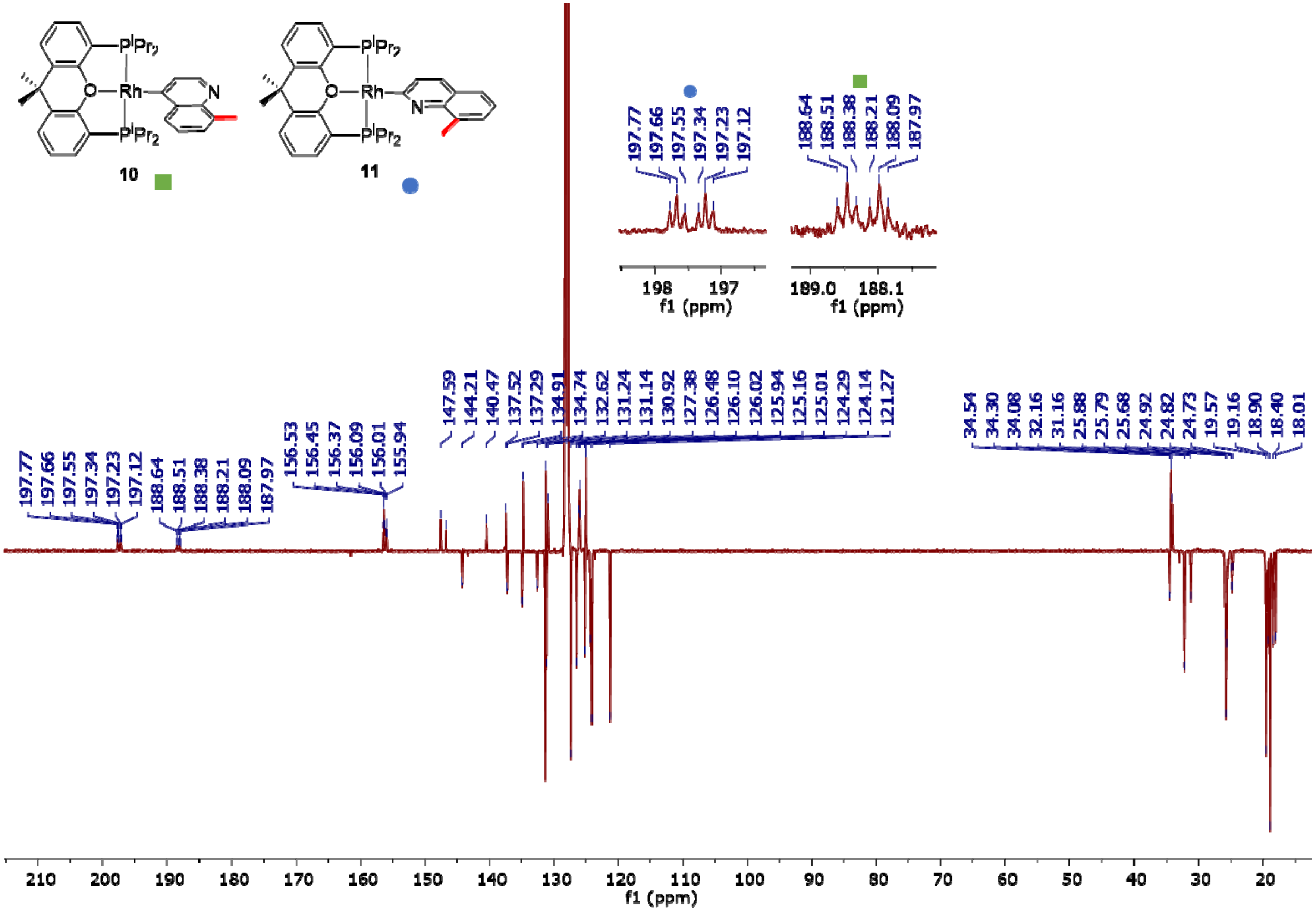

Figure S31. $^{13}C\{^1H\}$-apt NMR spectrum (100.62 MHz, benzene-d_6, 298 K) of a mixture of complexes **10** and **11**.

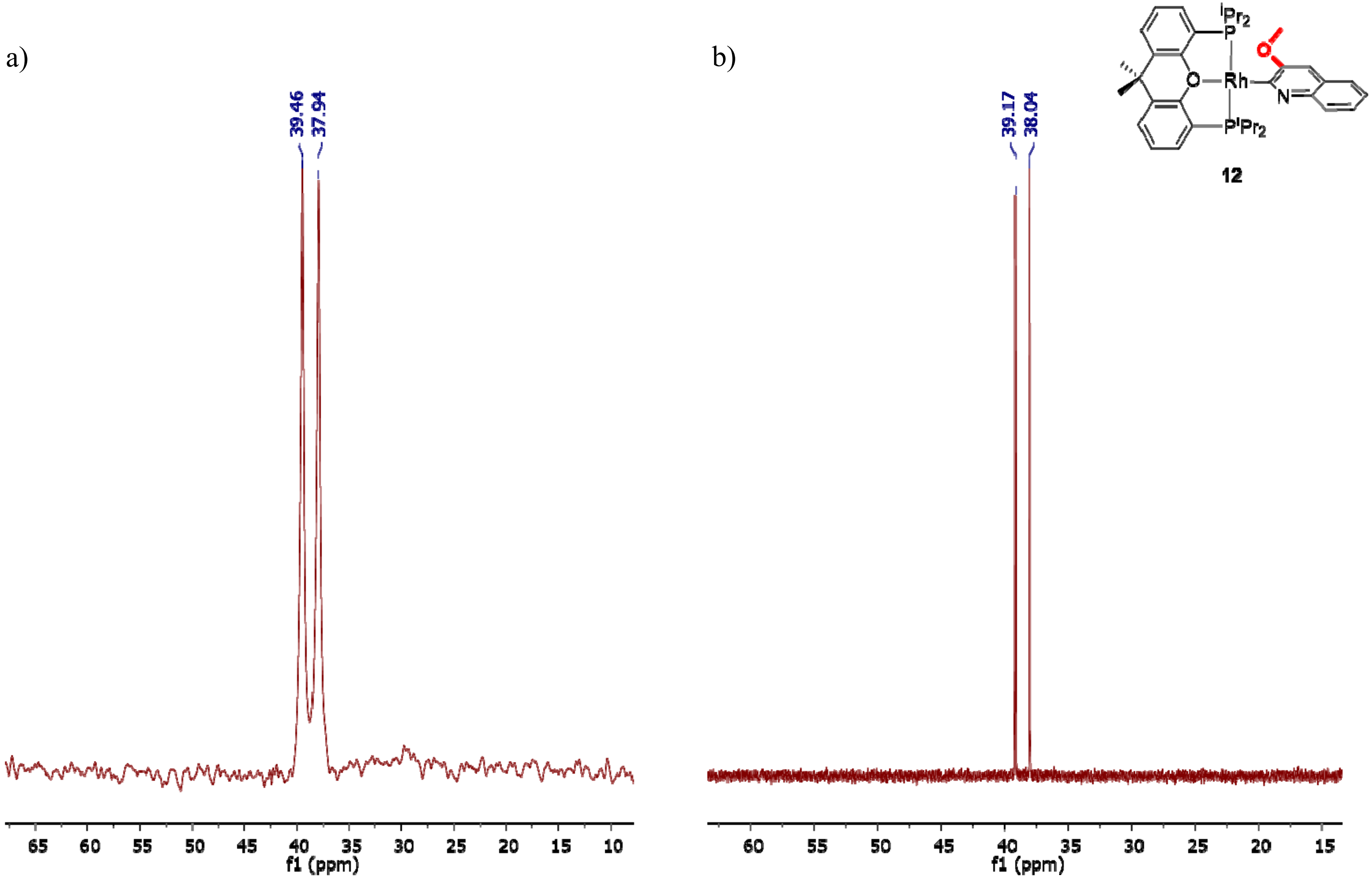

Figure S32. (a) $^{31}P\{^{1}H\}$ NMR spectrum (121.49 MHz, *n*-octane, 298 K) of the reaction of **1** with 3-methoxyquinoline after 72 h at 80 ºC. (b) $^{31}P\{^{1}H\}$ NMR spectrum (161.98 MHz, benzene-d_6, 298 K) of Rh(κ^1-C^2-quinolinyl-3-OMe){κ^3-P,O,P-[xant($P^{i}Pr_2$)$_2$]} (**12**).

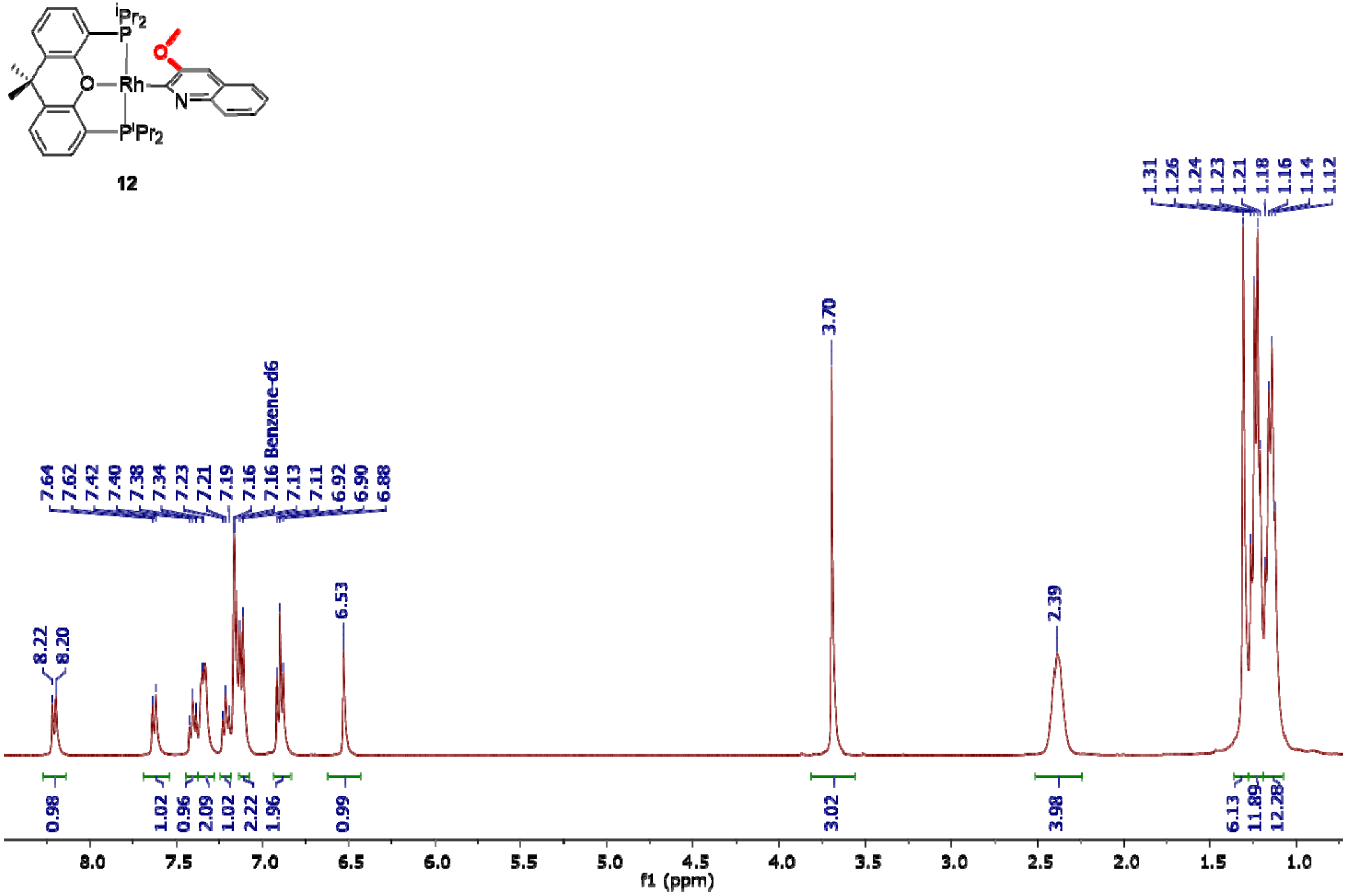

Figure S33. ^{1}H NMR spectrum (400.13 MHz, benzene-d_6, 343 K) of Rh(κ^1-C^2-quinolinyl-3-OMe){κ^3-P,O,P-[xant(P^{i}Pr$_2$)$_2$]} (**12**).

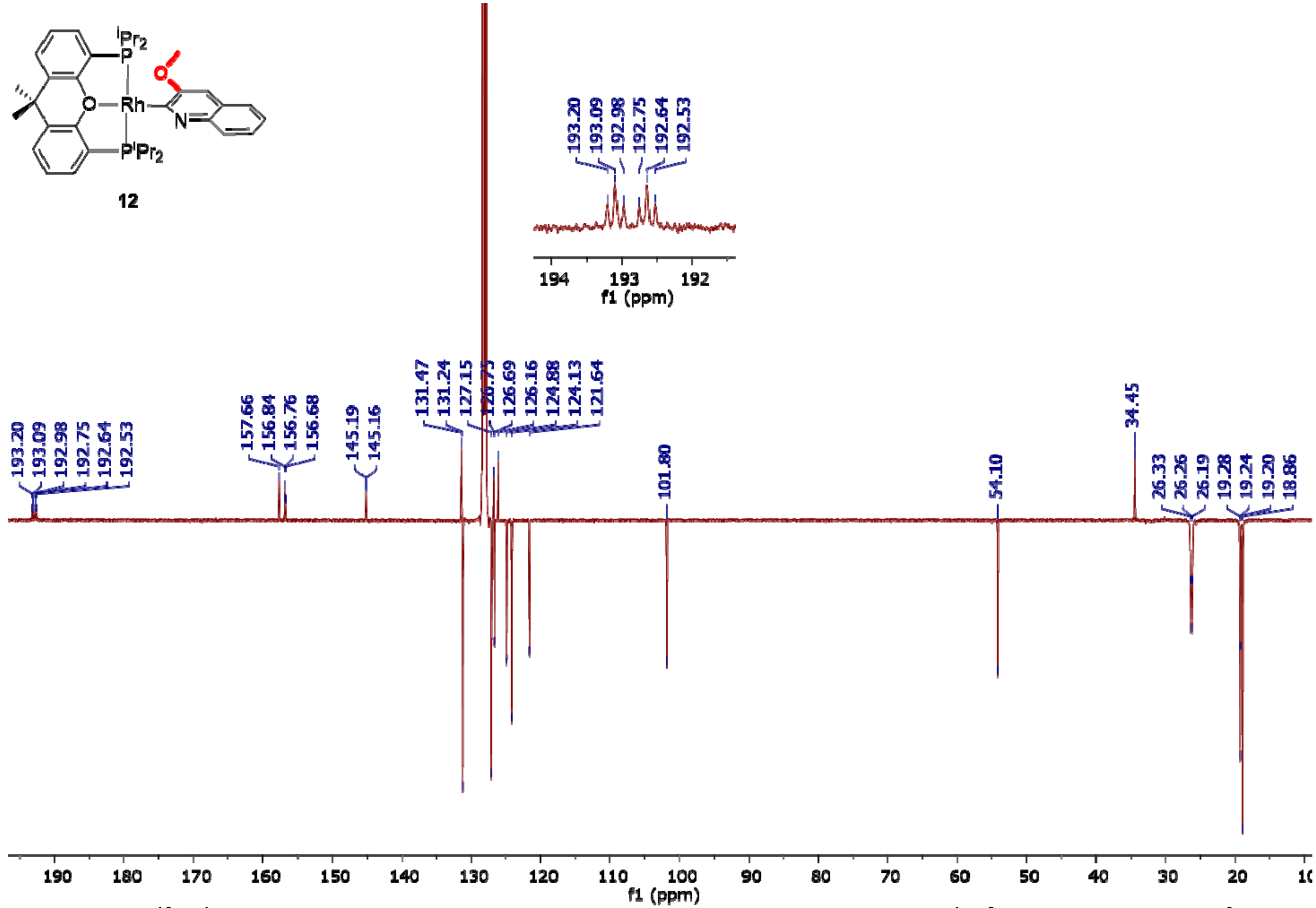

Figure S34. $^{13}C\{^{1}H\}$-apt NMR spectrum (100.62 MHz, benzene-d_6, 343 K) of Rh(κ^1-C^2-quinolinyl-3-OMe){κ^3-P,O,P-[xant(P^iPr_2)$_2$]} (**12**).

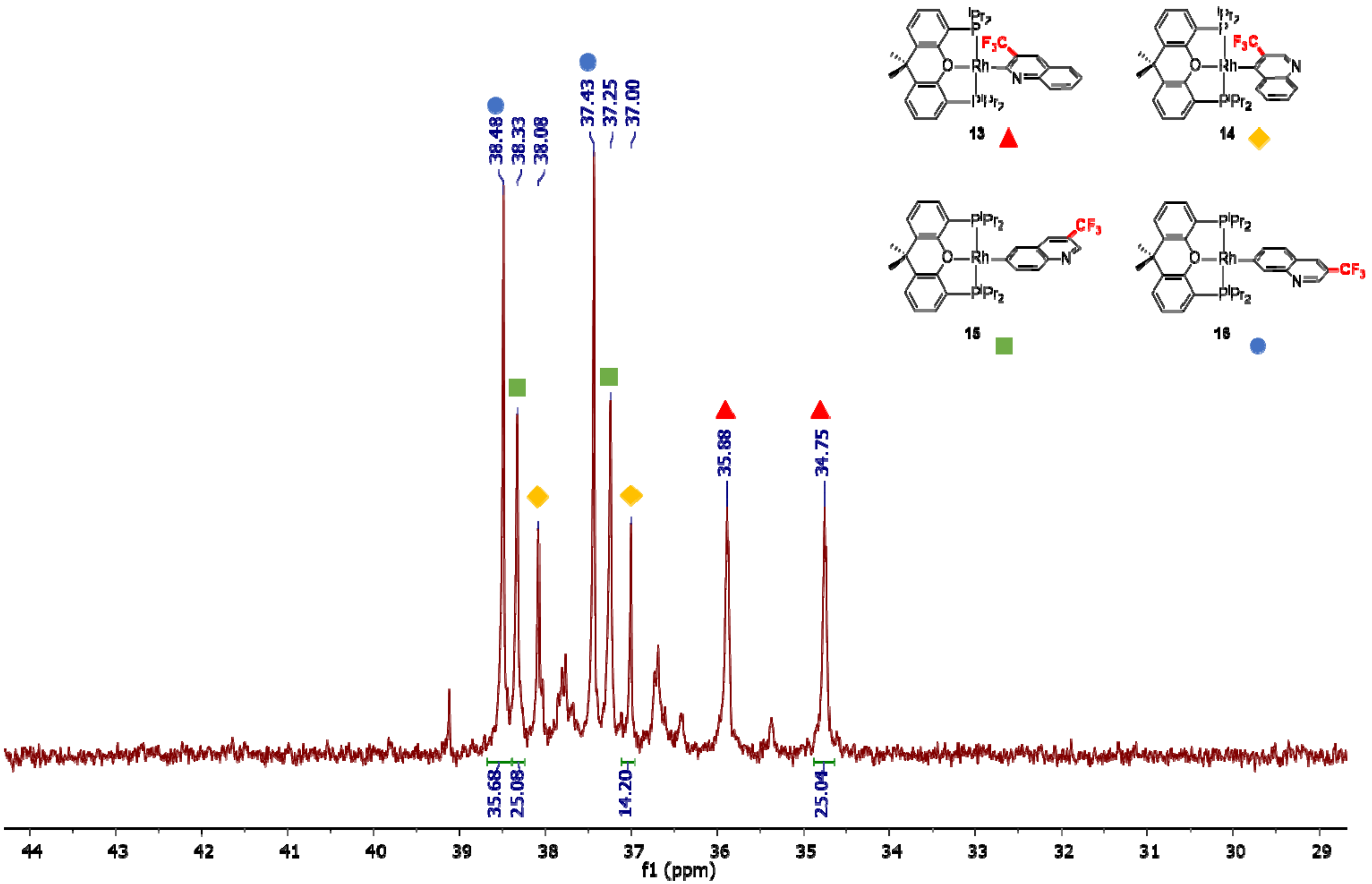

Figure S35. (a) $^{31}P\{^{1}H\}$ NMR spectrum (161.98 MHz, *n*-octane, 298 K) of the reaction of **1** with 3-(trifluoromethyl)quinoline after 5 days at 80 ºC.

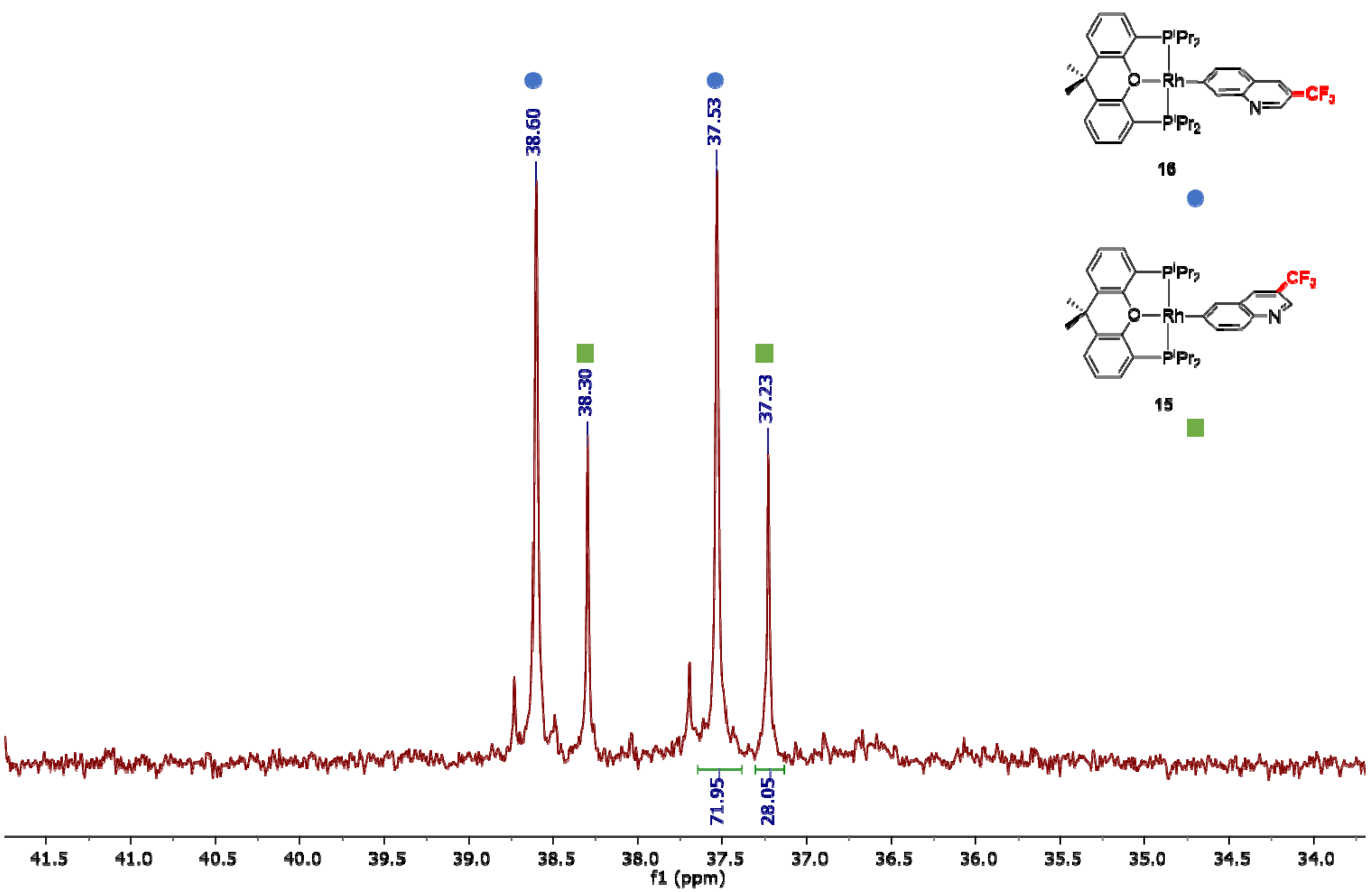

Figure S36. $^{31}P\{^{1}H\}$ NMR spectrum (161.98 MHz, benzene-d_6, 298 K) of a mixture of complexes Rh(κ^1-C^6-quinolinyl-3-CF_3){κ^3-P,O,P-[xant(P^iPr_2)$_2$]} (**15**) and Rh(κ^1-C^7-quinolinyl-3-CF_3){κ^3-P,O,P-[xant(P^iPr_2)$_2$]} (**16**).

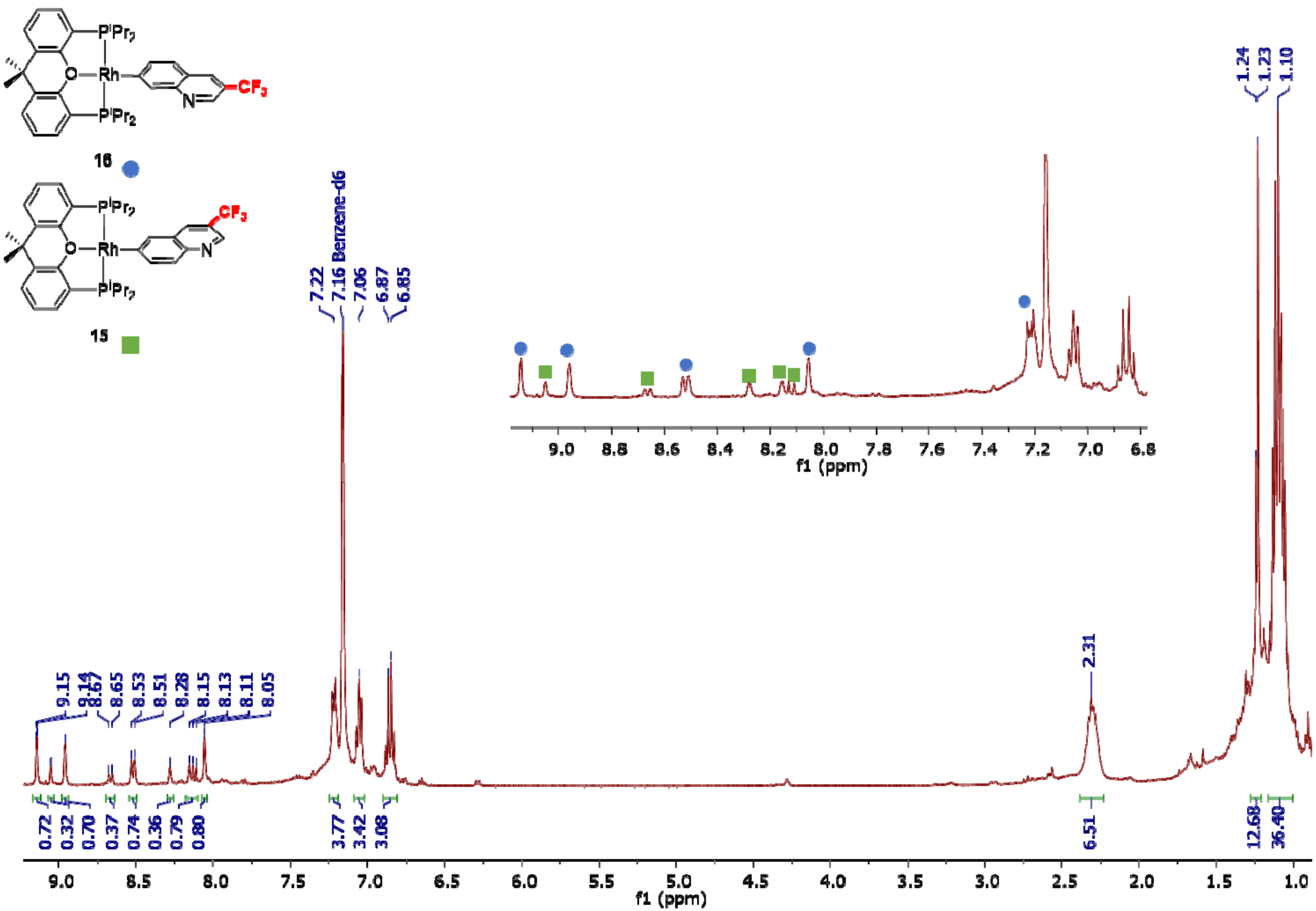

Figure S37. ^{1}H NMR spectrum (400.13 MHz, benzene-d_6, 298 K) of a mixture of complexes Rh(κ^1-C^6-quinolinyl-3-CF_3){κ^3-P,O,P-[xant(P^{i}Pr$_2$)$_2$]} (**15**) and Rh(κ^1-C^7-quinolinyl-3-CF_3){κ^3-P,O,P-[xant(P^{i}Pr$_2$)$_2$]} (**16**).

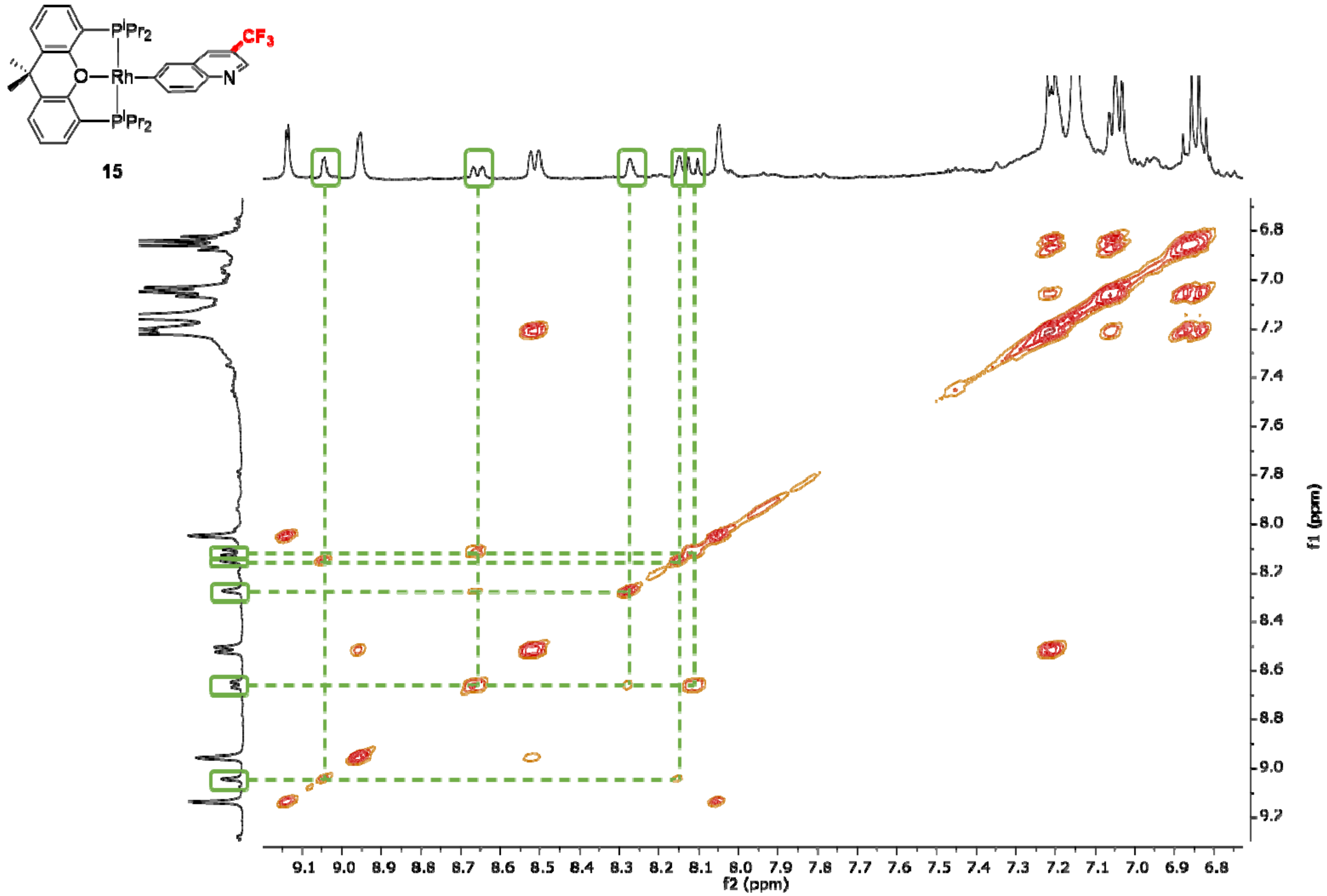

Figure S38. (^{1}H-^{1}H)-COSY NMR spectrum (400.13 MHz, benzene-d_6, 298 K) of a mixture of complexes **15** and **16**. Peaks corresponding to Rh(κ^1-C^6-quinolinyl-3-CF_3){κ^3-P,O,P-[xant(P^iPr_2)$_2$]} (**15**) are highlighted in green.

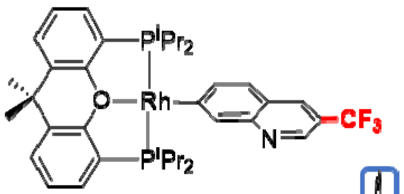

16

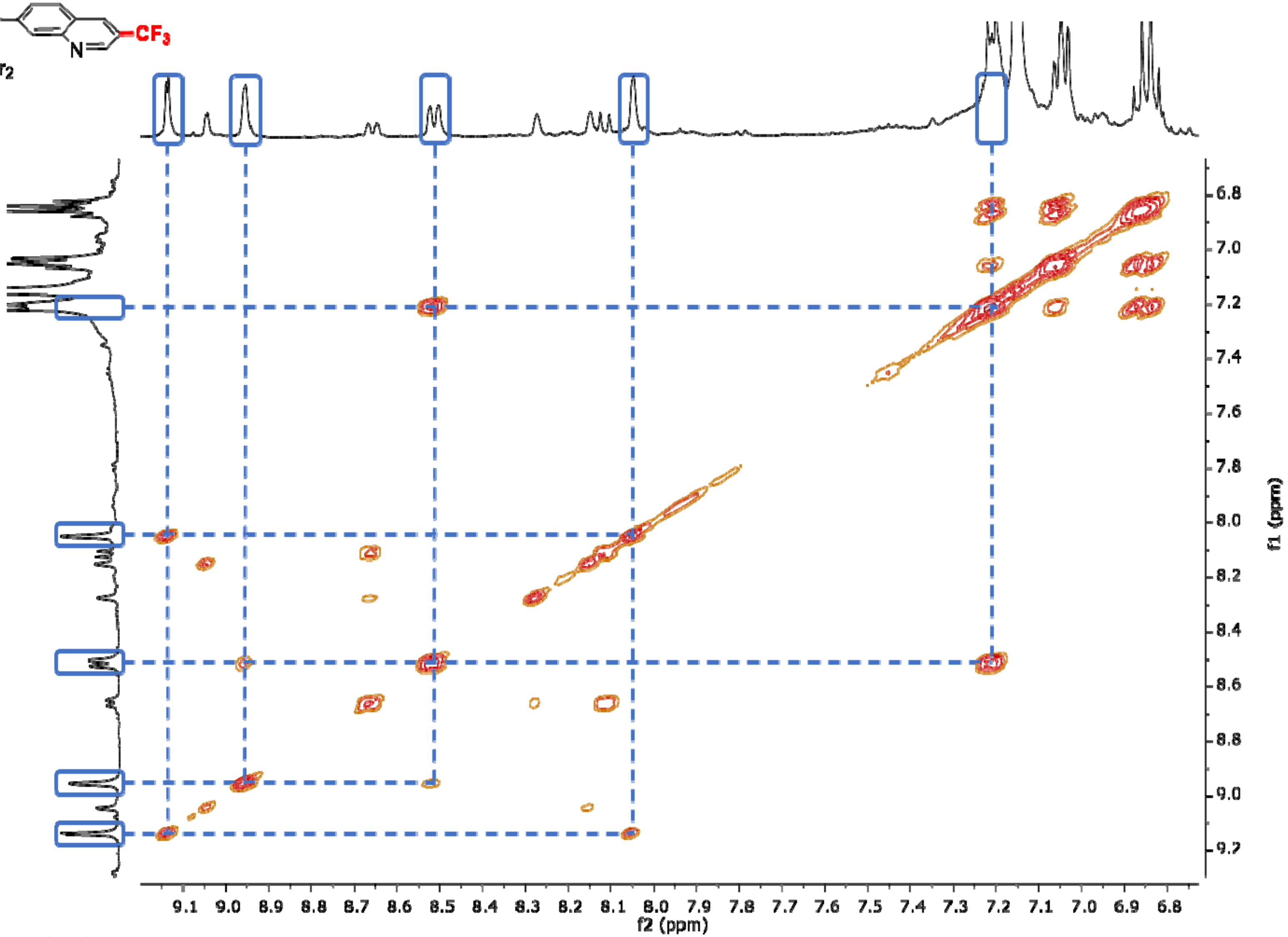

Figure S39. (^{1}H-^{1}H)-COSY NMR spectrum (400.13 MHz, benzene-d_6, 298 K) of a mixture of complexes **8** and **9**. Peaks corresponding to Rh(κ^1-C^7-quinolinyl-3-CF$_3$){κ^3-P,O,P-[xant(P^{i}Pr$_2$)$_2$]} (**16**) are highlighted in blue.

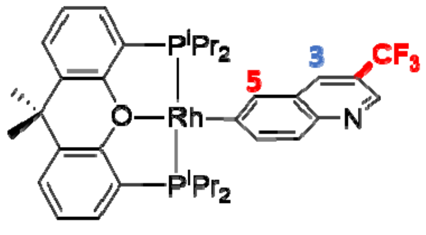

15

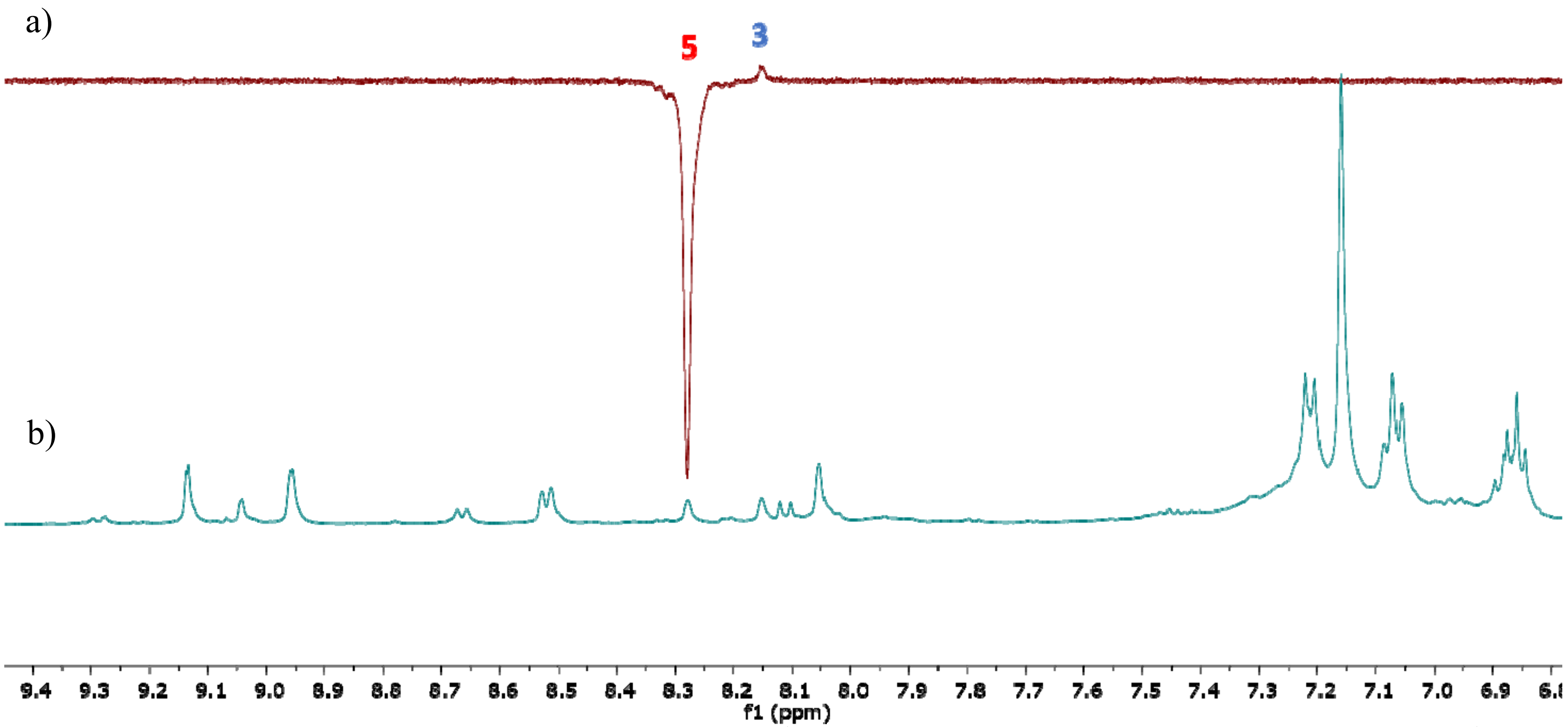

Figure S40. (a) Aromatic region of the one-dimensional ^{1}H selective NOESY experiment (d8 = 500 ms). (b) ^{1}H NMR spectrum (500.13 MHz, benzene-d_6, 298 K) of a mixture of complexes **15** and **16**.

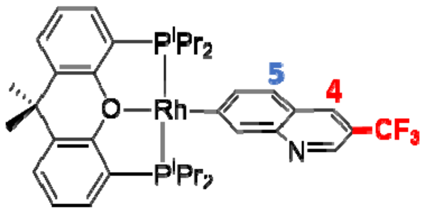

16

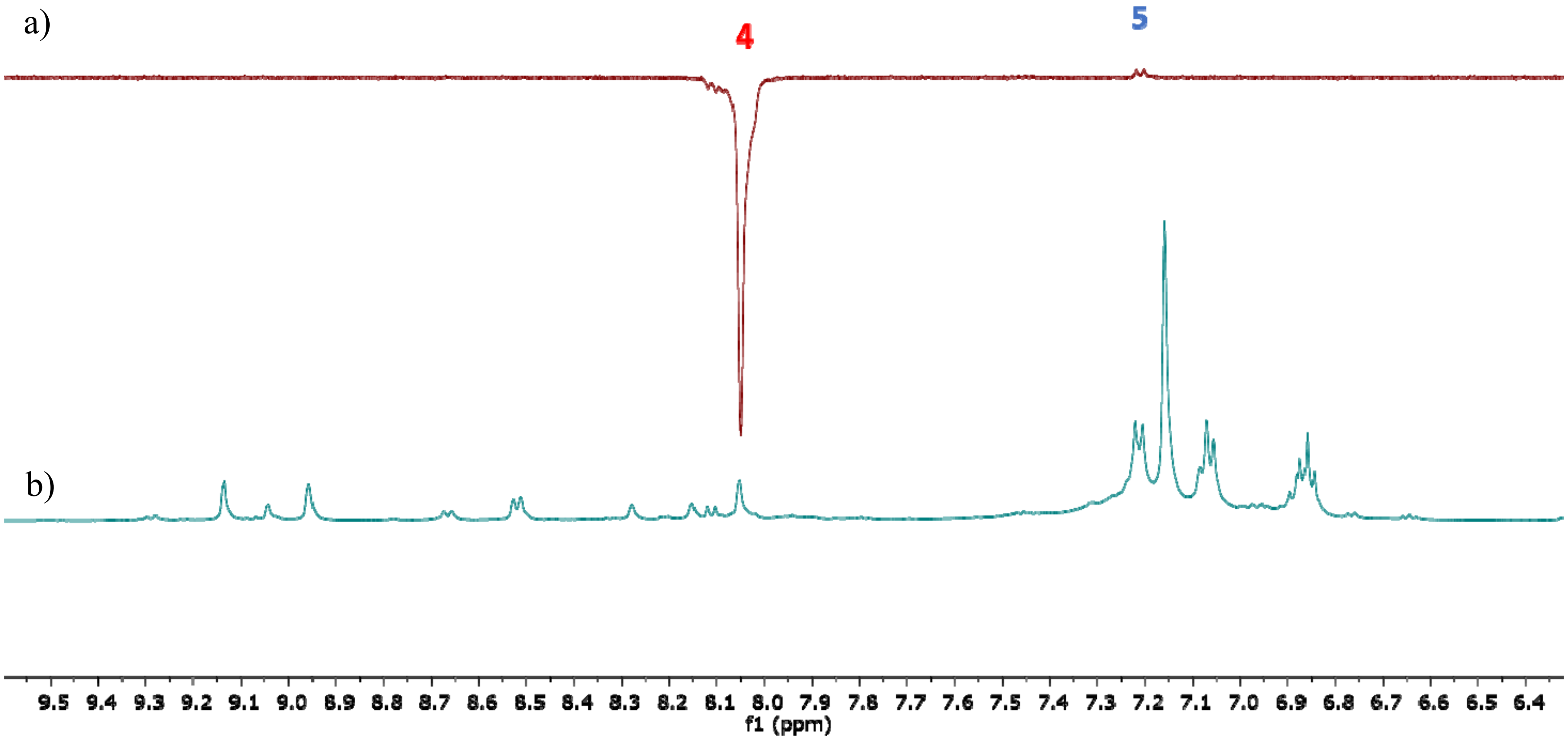

Figure S41. (a) Aromatic region of the one-dimensional ^{1}H selective NOESY experiment (d8 = 500 ms). (b) ^{1}H NMR spectrum (500.13 MHz, benzene-d_6, 298 K) of a mixture of complexes **15** and **16**.

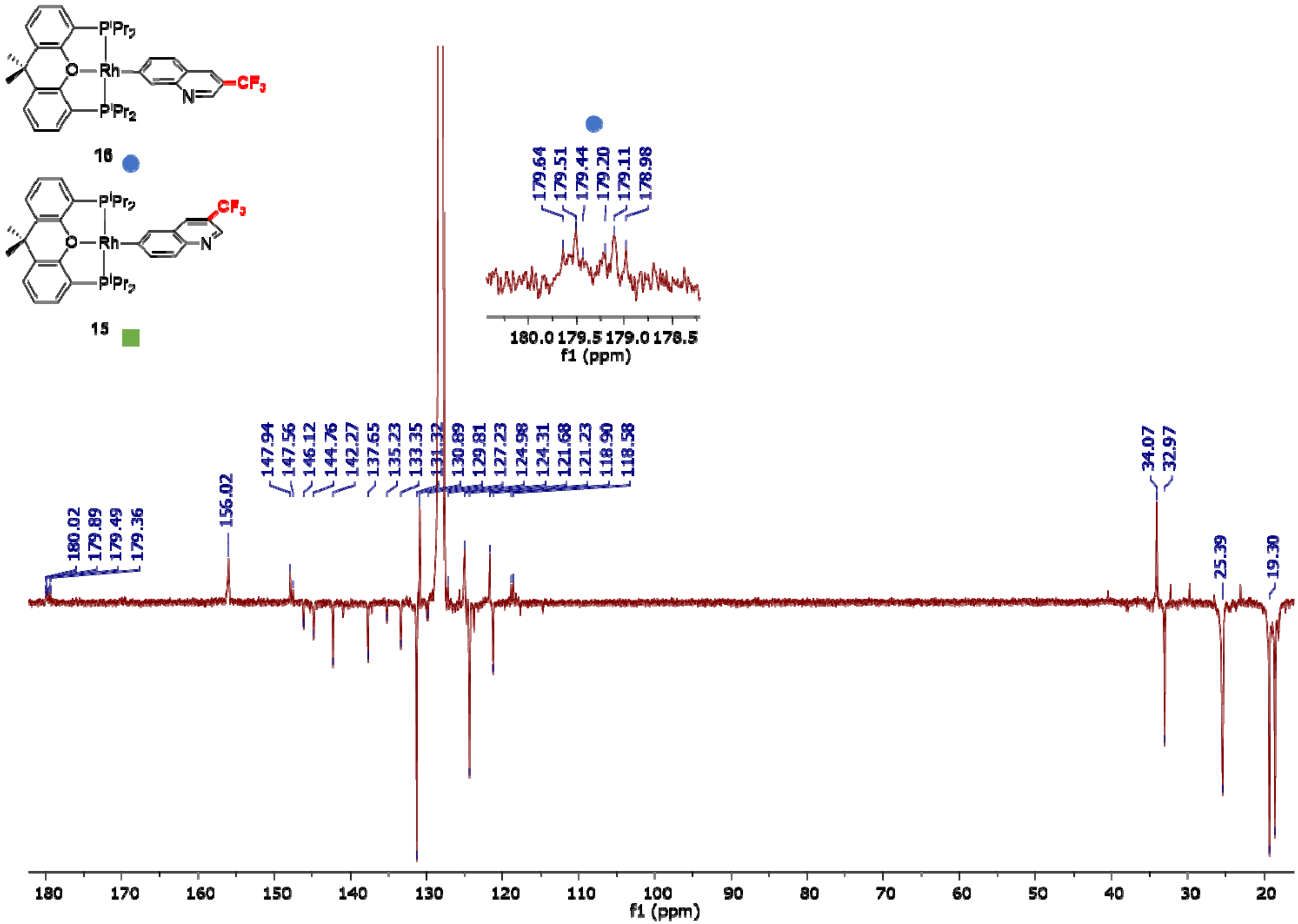

Figure S42. $^{13}C\{^1H\}$-apt NMR spectrum (100.62 MHz, benzene-d_6, 298 K) of a mixture of complexes Rh(κ^1-C^6-quinolinyl-3-CF_3)\{κ^3-P,O,P-[xant(P^iPr_2)$_2$]\} (**15**) and Rh(κ^1-C^7-quinolinyl-3-CF_3)\{κ^3-P,O,P-[xant(P^iPr_2)$_2$]\} (**16**).
